# Box Jellyfish (Cnidaria, Cubozoa) Extract Increases Neuron's Connection: A Possible Neuroprotector Effect

**DOI:** 10.1155/2021/8855248

**Published:** 2021-03-04

**Authors:** Gian Lucas M. Arruda, Hugo Vigerelli, Michelle C. Bufalo, Giovanna B. Longato, Rodinei V. Veloso, Vanessa O. Zambelli, Gisele Picolo, Yara Cury, André C. Morandini, Antonio Carlos Marques, Juliana Mozer Sciani

**Affiliations:** ^1^Laboratório Multidisciplinar de Pesquisa, Universidade São Francisco, Bragança Paulista 12916-900, Brazil; ^2^Laboratório de Genética, Instituto Butantan, São Paulo 05503-900, Brazil; ^3^Laboratório de Dor e Sinalização, Instituto Butantan, São Paulo 05503-900, Brazil; ^4^Laboratório de Pesquisa em Farmacologia Molecular e Compostos Bioativos, Universidade São Francisco, Bragança Paulista 12916-900, Brazil; ^5^Departamento de Zoologia, Instituto de Biociências, Universidade de São Paulo, São Paulo 05508-090, Brazil; ^6^Centro de Biologia Marinha, Universidade de São Paulo, São Sebastião 11612-109, Brazil

## Abstract

Neurodegenerative diseases are one of the major causes of death worldwide, characterized by neurite atrophy, neuron apoptosis, and synapse loss. No effective treatment has been indicated for such diseases so far, and the search for new drugs is being increased in the last years. Animal venoms' secretion/venom can be an alternative for the discovery of new molecules, which could be the prototype for a new treatment. Here, we present the biochemical characterization and activity of the extract from the box jellyfish *Chiropsalmus quadrumanus* (*Cq*) on neurites. The *Cq* methanolic extract was obtained and incubated to human SH-SY5Y neurons, and neurite parameters were evaluated. The extract was tested in other cell types to check its cytotoxicity and was submitted to biochemical analysis by mass spectrometry in order to check its composition. We could verify that the *Cq* extract increased neurite outgrowth length and branching junctions, amplifying the contact between SH-SY5Y neurons, without affecting cell body and viability. The extract action was selective for neurons, as it did not cause any effects on other cell types, such as tumor line, nontumor line, and red blood cells. Moreover, mass spectrometry analysis revealed that there are no proteins but several low molecular mass compounds and peptides. Three peptides, characterized as cryptides, and 14 low molecular mass compounds were found to be related to cytoskeleton reorganization, cell membrane expansion, and antioxidant/neuroprotective activity, which act together to increase neuritogenesis. After this evaluation, we conclude that the *Cq* extract is a promising tool for neuronal connection recovery, an essential condition for the treatment of neurodegenerative diseases.

## 1. Introduction

Neurodegenerative diseases are one of the major causes of death worldwide. The number of patients suffering from such diseases has been rising every year due to an increase in the life expectancy of the population. In 2015, 46.8 million people with dementia were reported worldwide, making it the 7th cause of death. The expectancy is that, in 2050, this number could reach 131 mi for Alzheimer solely [1–3].

The most common neurodegenerative diseases included Alzheimer's disease (AD), Parkinson's disease (PD), Huntington's disease (HD), and amyotrophic lateral sclerosis (ALS). All these pathologies are characterized by protein or peptide accumulation in certain regions of the brain, inside or outside neurons, after aggregation [2]. Key proteins involved in these processes are *β*-amyloid and tau for AD, *α*-synuclein for PD, huntingtin for HD, and ubiquitinated proteins for ALS [2].

Although the molecular and cellular mechanisms for neurodegeneration development have not been completely understood, it is known that precursor proteins are involved in the deposition of aggregates, which are not eliminated [4, 5]. Thus, it is suggested that the protein accumulation causes several damages to the brain, including an increase of reactive oxygen species, inflammation, and neuron apoptosis [6].

Neurite atrophy and synapse loss are frequent in such diseases, followed by an axonal degeneration, which probably initiates the neuronal death. The cell toxicity may cause impairment of cognition, memory, and muscle control, depending on the affected area of the brain [7, 8].

Molecules derived from natural products have been used as prototypes in the development of new drugs, including the treatment of neurological diseases [9]. Around 33% of the world's most selling drugs are derived from natural sources, both animals and plants, indicated for treating cancer, viral infections, pain, inflammation, and diseases of the central nervous system [10, 11].

Species from a marine habitat have provided several molecules of therapeutic interest; some of them already turned into commercial drugs and applied in the clinics [10, 12]. Examples are arabinose nucleosides from sea sponges for treating several types of cancer [13]. Bioactive molecules from marine animals are different from terrestrial ones in terms of structures—in the last years, 13,000 new molecules were identified from marine animals, with 3000 belonging to the phylum Cnidaria. Among them, there is no description of molecules from the class Cubozoa [14]. Nevertheless, these numbers reflect the ocean as an important source of new molecules, including terpenes, peptides, alkaloids, piperazines, lactones, nucleosides, and glycolipids, the most frequent ones [9, 13].

Several natural compounds, specially from plants, have proved to induce neurite length increase and regeneration. One example is platycodigenin, a terpenoid isolated from *Platycodon grandifloras* (Chinese bellflower), with known neuroprotective activity. Yang et al. [15] showed its ability in preventing A*β*25-35-induced neuronal death, besides promoting neurite regeneration. Another example is the alpha-toxin from the bacteria Clostridium perfringens that induces phosphorylation of TrkA through the phospholipid metabolism, and this pathway was related to the induction of neurite outgrowth in PC12 cells [16].

Compounds from marine animals have been poorly studied concerning neuron regeneration. A peptide fragment of thymosin *β*4, found in sea cucumbers and sea urchins, act as a regulator of neurogenesis, by facilitating the hippocampal neurogenesis and increasing spatial memory [17]. Moreover, a beta-thymosin from marine mollusks, and its peptide fragments, through the neuronal proliferation and the increase of neurite outgrowth [17, 18], supports the anchoring of neurons, besides increasing neurite regeneration (sprouting and total neurite outgrowth) in culture [18].

Glycosaminoglycans, isolated from sea squirts, octopuses, and sea urchins, display strong neuroregenerative activity, by promoting neurite outgrowth [19].

Jellyfishes (phylum Cnidaria) are abundant animals in many coastal areas around the world [20, 21]. They are known to cause unpleasant reactions on humans, due to its venom present in specialized intracellular organelles called nematocysts [22]. The venom of jellyfishes is known to cause several stings, mainly characterized by inflammation and pain [23]. The venom is constituted mainly by proteins: phospholipases A2, metallopeptidases, serinepeptidases, CRISPs, lectins, pore-forming toxins, and protease inhibitors [24–27]. Peptides have also been described; however, little is known about low molecular mass compounds from Cnidaria, regarding both structure and biological activity.

Moreover, there are some studies showing the abundance of neurotransmitters in cnidarians, such as acetylcholine, norepinephrine, serotonin, histamine, glutamate, and *γ*-aminobutyric acid (GABA), involved in the animal's physiology, including neurotransmission and neuromodulation [28].

Our goal was to verify the composition of the methanolic extract of the tentacle of the box jellyfish (*Chiropsalmus quadrumanus*), as well as its activity on human neurons, analyzing its potential for neurite and branch formation, which would be useful in diseases characterized by neuronal loss.

## 2. Materials and Methods

### 2.1. Preparation of Extract

**Figure 1:**
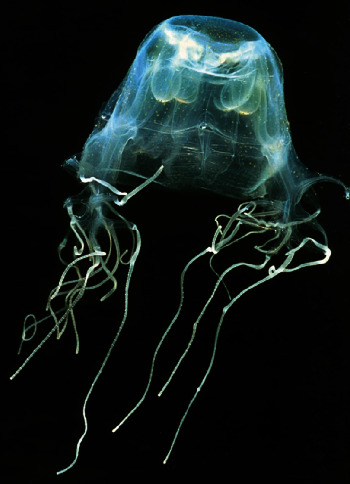
Box jellyfish *C. quadrumanus* collected for the study.

The box jellyfish *Chiropsalmus quadrumanus* (Cnidaria, Cubozoa, Chridropida, Figure 1—photo kindly provided by Dr. Alvaro E. Migotto and Cifonauta-CEBIMar) was collected in a marina at Ilhabela country, São Sebastião Island, São Paulo, Brazil (23° 46′ 23^″^ S, 45° 21′ 25^″^ W), under IBAMA license #16802-2. After collection, animals had their tentacles removed and placed in methanol containing 0.1% acetic acid for 48 hours (*Cq* extract). After that, the solution was centrifuged at 5,000 x g for 10 minutes and the supernatant was lyophilized. The content was resuspended in sterile phosphate buffer solution (PBS 50 mM, pH 7.4) for the cell experiments and in water methanol (1 : 1 vol : vol) for mass spec and SDS-PAGE analysis. Species identification and extra biological information may be found in Jarms et al. [29].

### 2.2. Cell Culture and Neuronal Differentiation Protocol

SH-SY5Y cells (ECACC, Sigma Aldrich, St. Louis, MO, USA) were cultured in a 1 : 1 mixture of Ham's F12 and Dulbeco's modified Eagle's medium (DMEM) (Gibco Life Technologies, Grand Island, NY, USA) supplemented with 10% heat-inactivated fetal bovine serum (FBS) (HyClone Labs., Logan, UT) and 100 U/mL of penicillin/streptomycin (Gibco Life Technologies, Grand Island, NY, USA) in a humidified atmosphere of 5% CO_2_ at 37°C. The medium was changed twice a week, and cells were split at about 80% confluence. For neuronal differentiation, 5 × 10^4^ cells/well was seeded in collagen-coated plates (100 *μ*g/mL, Corning, USA). After 24 hours (day 1), the medium was replaced by medium in which FBS concentration was reduced to 2% (differentiation medium) and supplemented with 10 *μ*M all-trans retinoic acid (RA, Sigma Aldrich, Saint Louis, MO). Cells were incubated for 5 days, with the medium replaced every day, except on the second day. At day 5 of differentiation, the medium was removed, and cultures were stimulated with serum-free medium supplemented with human brain derived neurotrophic factor (BDNF 50 ng/mL—R&D Systems, MN, USA). At day 7 of differentiation, neurons were used in the experiments.

### 2.3. Neurite Outgrowth Assay Using High-Content Screening (HCS) Platforms

Neurons were treated with the *Cq* extract for 24 hours with 2, 10, and 100 *μ*g/mL. After treatments, neurons were fixed for 30 min at room temperature in 3.7% paraformaldehyde (Sigma-Aldrich, Saint Louis, MO) in phosphate-buffered saline (PBS, Sigma-Aldrich, Saint Louis, MO) at pH 7.4. After washing in PBS, cells were permeabilized for 5 minutes in 0.5% Triton X-100 (Sigma-Aldrich, Saint Louis, MO) in PBS and washed 3 times for 10 minutes, also in PBS. Samples were blocked for 1 hour at room temperature with 3% bovine serum albumin (BSA) (Amresco, MA, USA) in PBS. Cultures were incubated overnight at 4°C with chicken anti neuron specific *β*-III tubulin primary antibody diluted in PBS and 3% BSA (1 : 500; Merck Millipore MA, USA). After this period, cells were incubated with secondary PE-conjugated goat anti-chicken antibodies 1 : 500 (Cell Signaling Technology, MA, USA) for 1 hour at room temperature. Nuclei were stained using nuclear dye DAPI 1 : 200 (Gibco Life Technologies, Grand Island, NY, USA), and neurite analysis was performed using HCS according to the following parameters:

- Total outgrowth: total amount of skeletonized outgrowth in *μ*m (corrected for diagonal lengths) associated with the cell
- Mean process length: total outgrowth (in *μ*m) divided by number of processes of the cell
- Process: number of outgrowths connected to the cell body
- Branches: number of branching junctions of all the processes connected to the cell
- Total cell body area: total *μ*m^2^ of the cell bodies in the image (excluding outgrowths)
- Straightness: ratio varying between 0 (not straight) and 1 (perfectly straight) defined as end-to-end Euclidean distance between segment junctions divided by corresponding actual neurite curve length (the sum of end-to-end lengths divided by the sum of curve lengths), averaged over all of the cells in the image

### 2.4. Cell Viability

The *Cq* extract, in a range of 1.6 to 100 *μ*g/mL, was tested in a cell panel of human cultured cells of neuroblastoma (SH-SY5Y), glioblastoma (U-251), breast (MCF7) and ovary (OVCAR-3) adenocarcinoma, multidrug-resistant ovary (NCI-ADR/RES), leukemia (K-562) and nontumoral keratinocytes (HaCaT), obtained and cultured according to the instructions of American Type Culture Collection (ATCC, Manassas, VA). Cells were maintained in a humidified 5% CO_2_ incubator at 37°C. After 48 hours of treatment, the cell viability was determined by 3-(4,5-dimethylthiazol-2-yl)-2,5-diphenyltetrazolium bromide (MTT) assay, where the medium was discarded, and the reagent was incubated for 4 hours in a concentration of 0.5 mg/mL. The blue formazan product was dissolved in dimethyl sulfoxide (DMSO), and the absorbance was measured in a microplate reader (EPOCH, BioTech Instrument Inc., USA) at 540 nm.

The results were plotted in a graph of % viable cells according to the extract concentration, and IC50 was calculated.

Alternatively, the *Cq* extract was incubated in human red blood cells (RBC; approved by the Research Ethics Committee from USF—CAAE 25441719.0.0000.5514). The total blood was collected in EDTA tubes from 5 volunteers and pooled for being centrifuged at 1000 x g for 10 minutes under room temperature. RBC were separated and washed with PBS (50 mM, pH 7.3). Then, RBC 4% suspension was obtained and 40 *μ*L were mixed with 100 *μ*L PBS and 100 *μ*g/mL *Cq* extract. The reaction was incubated by 60 minutes at 37°C and then centrifuged at 4000 x g for 5 minutes under room temperature. The supernatant was placed in a 96-well plate, and the absorbance was measured in a spectrophotometer at *λ* = 414 nm.

As a negative control, PBS was used instead the *Cq* extract, and for positive control, 0.1% Triton-X 100 was added.

### 2.5. Biochemical Analysis

#### 2.5.1. Mass Spectrometry

The *Cq* extract was submitted to a chromatography coupled to a mass spectrometry for peptides and low molecular compound analysis.

The extract was firstly analyzed in a reversed-phase ultraperformance liquid chromatography (RP-UPLC). Aliquots of the samples were loaded in a C18 column (ACE C18, 5 m, 100 Å, 2.0 mm × 50 mm) in a two-solvent system: (A) formic acid (FA)/H2O (1 : 1000) and (B) FA/acetonitrile/H_2_O (1 : 900 : 100). The content was eluted at a constant flow rate of 0.2 mL/min with a 0–100% gradient of solvent B over 40 min, after a 5 min isocratic elution with 0% B. The UPLC column eluates were monitored by a mass spectrometry (Q-ToF Xevo GS, Waters Co.), in a positive ionization mode, in a range of 200 to 1800 *m*/*z* and FWHM 40000 resolution at 500 *m*/*z*. For the MS/MS analysis, argon collision energy was applied. The instrument control and data acquisition were conducted by MassLinx 4.2.

The results were automatically processed by PEAKS®7.0 software (Bioinformatics Solution Inc.) and then manually verified. De novo sequences were considered when average local confidence (ALC %) was >50. Peptides sequenced were submitted to protein BLAST (Basic Local Alignment Search Tool), in order to find similar peptides already described. For this analysis, the database nonredundant protein sequences (nr) was used, with organism selection of Cnidaria (taxid: 6073) and blastp algorithm, with parameters of 10 expected threshold, matrix BLOSUM62, and gap existence 11. In parallel, de novo sequences were searched against peptide databank (PepBank) [30].

Alternatively, a fingerprinting of low molecular mass compounds was performed by the spectra analysis on Progenesis QI Software (Waters Co.). Molecules were identified by spectra similarity with the HMDB database, exact molecular mass, and *m*/*z*.

#### 2.5.2. SDS-PAGE

SDS-PAGE (12%) was performed to analyze proteins on the *Cq* extract under reducing and nonreducing conditions. An aliquot (10 *μ*g, determined by dry weight) was applied in the gel, and a constant voltage of 120 V was applied. After the run, the gel was stained with silver, according to the method described by Laemmli [31].

### 2.6. Statistical Analysis

All the cell experiments were performed in triplicate, and the results are shown as mean ± standard deviation. The treatment with compounds (3 groups) was compared to the negative control (same condition without treatment) by one-way ANOVA, followed by Tukey's posttest. Significance was considered if *p* < 0.05.

## 3. Results

### 3.1. Neurons Analysis

**Figure 2:**
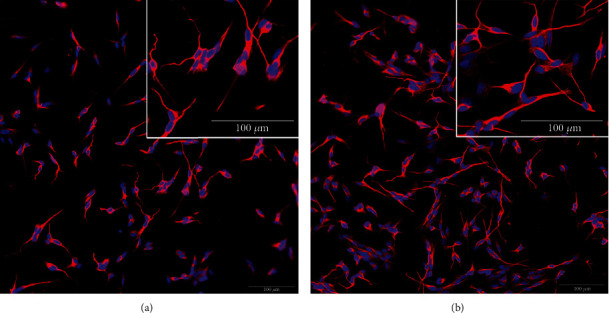
Neurons derived from the SH-SY5Y cell line. (a) Control, without treatment. (b) After 10 *μ*g/mL *C. quadrumanus* extract incubation. Inset: a zoom to show cell details.

Neurons derived from the SH-SY5Y cell line were analyzed after treatment with the *Cq* extract, in order to investigate its impact on neurite outgrowth. It was possible to verify neurites and branches without the treatment and after the neuron's differentiation (Figure 2(a)). However, 10 *μ*g/mL *Cq* extract clearly increased the neurites' length, with apparent enhanced contact between them (Figure 2(b)).

**Figure 3:**
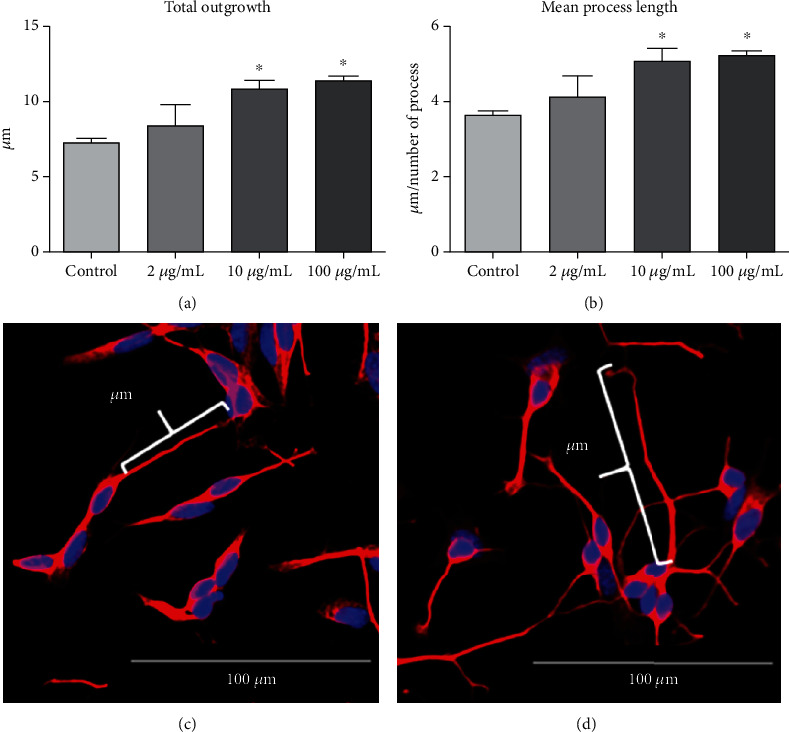
Parameters of outgrowth measured in the neurons derived from the SH-SY5Y cell line after the incubation of *C. quadrumanus* extract. (a) Quantification of total outgrowth, measured in *μ*m. (b) Mean of the process length, obtained after quantification of total outgrowth (*μ*m) divided by the number of processes. (c) Representative image of an outgrowth of control neurons. (d) Representative image of an outgrowth of neurons treated with 10 *μ*g/mL *Cq* extract. ^∗^Statistical difference *p* < 0.05.

The outgrowth (in *μ*m) was quantified, and 10 and 100 *μ*g/mL of extract significantly increased the total outgrowth length, as shown in the quantification of Figure 3(a).

When we calculated the ratio between length and number of outgrowths (mean process length), an increase after *Cq* extract treatment was observed, in both concentrations of 10 and 100 *μ*g/mL, confirming the previous result, but now analyzed by each cell (Figure 3(b)). The increase of neurite length is evident after the extract treatment, as depicted in Figure 3(d), in comparison to the control, without treatment, in Figure 3(c), analyzed in the same scale.

**Figure 4:**
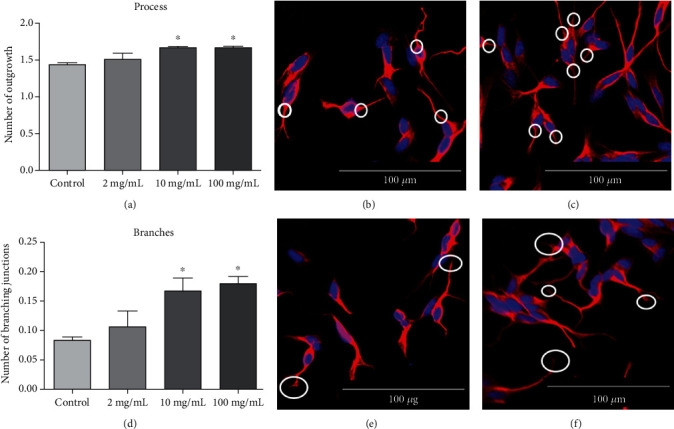
Processes and branches of neurons derived from the SH-SY5Y cell line after the incubation of the *C. quadrumanus* extract. (a) Quantification of number of outgrowths connected to the cell body (process). (b) Representative image of processes from control neurons. (c) Representative image of processes from neurons treated with 10 *μ*g/mL *C*. *quadrumanus* extract. (d) Number of branching junctions. (e) Representative image of branches from control neurons. (f) Representative image of branches from neurons treated with 10 *μ*g/mL C. quadrumanus extract. The white circles indicate the measured structures. ^∗^Statistical difference *p* < 0.05.

Besides the length, the number of outgrowths that connects to the cell body (process) was significantly increased as well, with the same extract concentrations (10 and 100 *μ*g/mL).  Figure 4(a) shows the quantification of processes and Figures 4(b) and 4(c) show the neuron with processes indicated by white circles from the control and treated neurons, respectively.

Moreover, the number of branching junctions of all the processes (connected to the cell) was also increased significantly with 10 and 100 *μ*g/mL *Cq* extract, as shown in the quantification graph (Figure 4(d)) and in the image in Figures 4(e) (control) and 4(f) (*Cq* treated).

**Figure 5:**
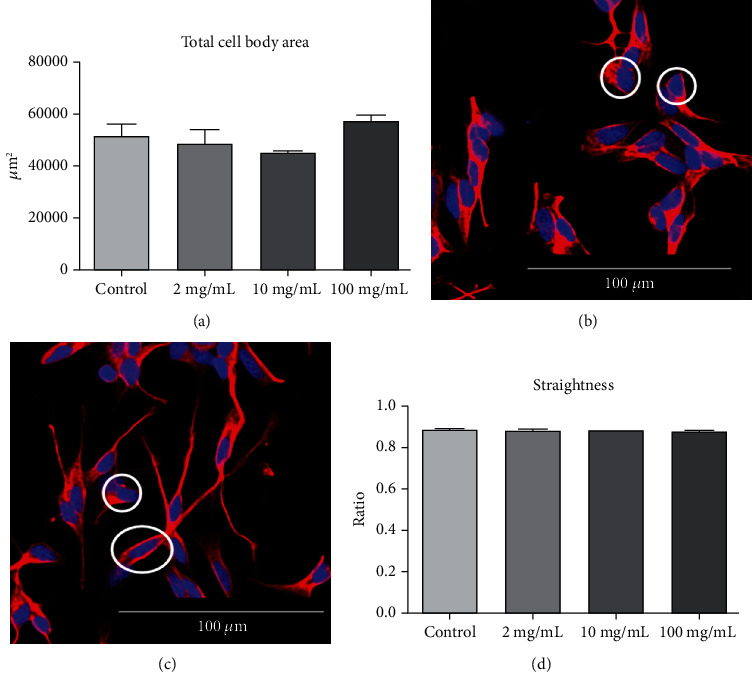
Body cell and straightness of neurons derived from the SH-SY5Y cell line after the incubation of *C. quadrumanus* extract. (a) Quantification of total body cell area (*μ*m2). (b) Representative image of the total body cell area from control neurons. (c) Representative image of the total body cell area from neurons treated with 10 *μ*g/mL *Cq* extract. (d) Ratio between not straight and perfectly straight. The white circles indicate the measure structures.

On the other hand, the cell body area was not affected with the treatment, indicating the Cq extract effect only on outgrowths.  Figure 5(a) shows the quantification, while neuron images are shown in Figures 5(b) (control) and 5(c) (*Cq* extract).

The straightness was measured in order to verify if the path of a neurite's growth would be deviated from a straight line. Even 100 *μ*g/mL *Cq* extract could not cause any effect on this parameter (Figure 5(d)).

### 3.2. Cell Viability Analysis

**Figure 6:**
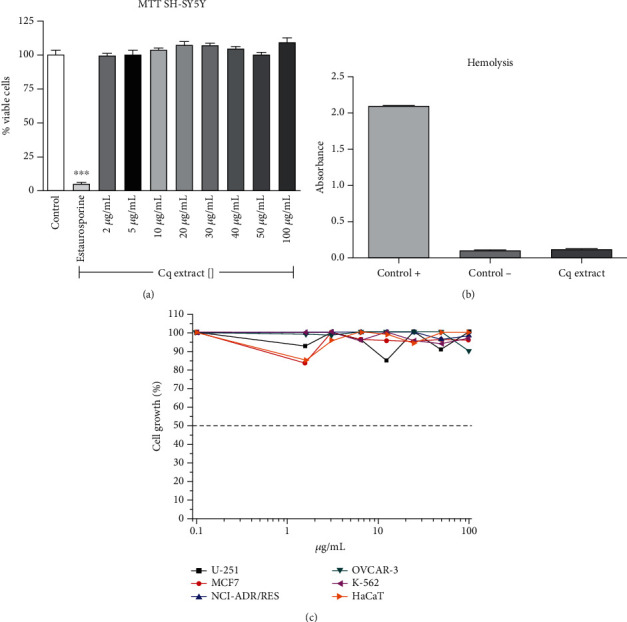
Cell viability after treatment with *C. quadrumanus* extract. (a) Cell viability assay by MTT after *Cq* extract treatment on SH-SY5Y. (b) Hemolysis rate of red blood cell after the treatment of *Cq* extract; control-: PBS incubation and control+: Triton-X 100 incubation. (c) Cell viability assay by MTT after *Cq* extract treatment on cell line panel (U-251, MCF7, NCI-ADR/RES, OVCAR-3, K-562, and HaCaT).

Although there is an apparent increase in number of cells when the images are analyzed (e.g., Figure 2(b)), an MTT assay was performed to check if the *Cq* extract could induce neuron's proliferation or even toxicity. As shown in Figure 6(a), the extract did not cause any SH-SY5Y proliferation. Importantly, the extract did not cause cytotoxic effect, even in the highest concentration evaluated (100 *μ*g/mL), what confirms its activity only on neurites and not in the cell body.

The extract did not interfere with red blood cell viability as well, by inducing only 1.3% hemolysis, compared to the positive control, Triton-X 100, and negative control saline solutions (Figure 6(b)). In order to confirm that the *Cq* extract would not induce alteration on viability of other lines, it was evaluated in a panel consisting of glioblastoma, breast and ovary adenocarcinoma, multidrug-resistant ovary, leukemia, and keratinocytes. In all the tested concentrations (0.1 to 100 *μ*g/mL), the extract did not cause any cytotoxic effect or cell proliferation (Figure 6(c)), reinforcing its activity only on neurites.

### 3.3. Biochemical Analysis

**Figure 7:**
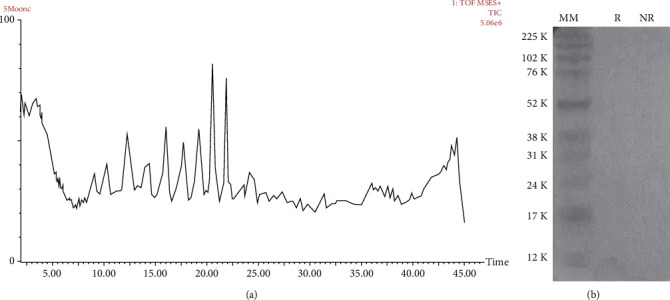
Profile of *C. quadrumanus* extract. (a) TIC chromatogram obtained after mass spectrometry analysis. (b) SDS-PAGE of reduced (R) and nonreduced (NR) conditions of the extract.

In order to analyze the chemical composition of the *Cq* extract and identify important molecules for neuron or neurite regeneration, the extract was submitted to a reversed-phase chromatography coupled to a mass spectrometry. This method was set to verify the presence of peptides and low molecular mass compounds. In a general profile, it was possible to see qualitatively high abundance of molecules, due to the presence of several peaks along the chromatographic gradient (Figure 7(a)). Through SDS-PAGE, we verified that the extract did not contain proteins: the extraction with methanol was able to remove all the proteins (secreted or constitutive), and only low molecular mass compounds were obtained for the cell's test (Figure 7(b)).

Regarding peptides, we could identify and sequence thirteen, shown in Table 1. These sequences were searched in the PepBank, and no description/activity was found for any of them. However, when searched in a protein database (BLAST), the peptides matched proteins with high score. The proteins are related somehow to neuron regeneration and with high score and identity (Table 2).

The low molecular mass compounds were identified as well, and the complete list of molecules is in the Supplementary Table 1. However, the molecules related to neuron regeneration could be identified and are shown in Table 3.

## 4. Discussion

Neurodegenerative diseases have no specific treatment, and the therapies currently available are applied for increasing the patient's life expectation in some years, not representing a cure [32]. Thus, the search for new drugs for such diseases is essential, as well as the understanding of the mechanisms behind their effects. In this context, the research involving natural products is increasing and bioactive molecules have been explored for drug discovery and development.

Considering that cnidarians from Cubozoa is rarely explored in terms of biotechnological application, we have obtained a methanolic extract in order to verify its composition and to study preliminary applications on neurite growth for the application of neuron network regeneration.

In the present work, we show, for the first time, that the molecular composition of the methanolic extract of *C. quadrumanus* is associated with a relevant biological effect, other than envenomation. We demonstrated that peptides and small molecules from the Cubozoa jellyfish extract act with synergy in different mechanisms to increase the neurite length, processes, and branches.

The human SH-SY5Y neuroblastoma cell line is frequently used for different neuronal cell culture models, including neurodegenerative disorders, and has been chosen for *C. quadrumanus* extract evaluation. Neurite evaluation has already been standardized for such cells, with similar methods applied in this work, regarding the incubation of natural products aiming neurite elongation, different measures into the cells, and time of analysis [33]. Therefore, we analyzed the neurons 24 hours after *Cq* extract incubation, time enough to promote neurite elongation, as observed here, and also enough to activate intracellular pathways that would cause changes in the cell body, not observed in any tested concentration. These data reinforce the selective action of the extract on neurites.

SH-SY5Y can be differentiated into neuron-like phenotype cells, essential for functional analyses in neurosciences [34]. The neuronal differentiation process was performed using retinoid acid and BDNF, in a method already standardized [35, 36]. Neurotrophic factors have been used for a neuritogenic effect and neuronal regeneration; however, they have presented several disadvantages, such as the difficulties to cross the blood-brain barrier and inactivation/cleavage by peptidases from blood and tissues. Thus, the small molecules and peptides obtained from natural products can overpass these issues, by presenting neuritogenic activity with good pharmacokinetics properties [33].

It is important to mention that the proper neuronal function depends on the maintenance of axons and dendrites (collectively known as neurites) contributing to the precise neuronal network, which are essential for the establishment of synapses [37]. Thus, the neurite length is essential for the entire cell recovery.

Thus, we have found molecules that act on neuron's differentiation and growth, such as folinic acid and Pteroyl-D-glutamic acid, two derivative molecules from folic acid, a known chemoprotectant that participates of the nerve injury repair, by acting on the proliferation and migration of Schwann cell, and secretion of nerve growth factor [38]. (6s)-5-methyltetrahydrofolic acid compounds were patented to treat diseases associated with nervous system injuries, for example, amyotrophic lateral sclerosis and Alzheimer's disease [39].

Indeed, it is important to mention that the *Cq* extract did not induce any cell death, in several cell lines tested, which shows that the *Cq* extract does not interfere with the neurons body, regarding plasmatic membrane, cell signaling for necrosis or apoptosis or even proliferation. It was clear that the extract alters only the neurite-related structures.

Here, the neurites were evaluated by the measurement of length of total skeletonized outgrowth associated with the cell, which has increased. Moreover, we observed increase in the average, which means that the effects on neurite length was not an isolated phenomenon, but it was applied to all cells present in the culture, reinforcing the activity of the extract.

By the identification of molecules present in the *Cq* extract, it was possible to correlate them to mechanisms for neuritogenesis: we could identify molecules that act on the organization of neuron's cytoskeleton that contributes to the neurite expansion. Moreover, we have identified molecules that act on the cell membrane formation, necessary to follow the neurite expansion. Antioxidants were found as well that contributes to the neuron regeneration, with mechanism still unknown, besides neurotransmitter-like molecules that act on neuronal plasticity and neuroprotective compounds. So, the action of several molecules in all those mechanisms of action contributes to the significantly neuritogenic effect observed here.

The neurite formation is the first step for the axon and dendrite synthesis, essential for the development of a functional neuronal network, and takes part of the regeneration process after a nervous system injury. For neurite formation, the cytoskeleton has an important role, where actin, microtubule networks, and neurofilaments are essential [40].

A cryptide from our extract derived from translin-like protein is one molecule that contribute to neurite formation, related to microtubules and motor proteins. When augmented, they are associated with learning and memory, locomotor activity, anxiety-related behavior, and sensorimotor gating [41]. Tyrosine-protein kinase ABL1-like is activated after stimuli related to cytoskeletal reorganization [42].

The hyccin, another cryptein found here, have importance in the neuron membrane, as it was demonstrated to regulate the synthesis of phosphatidylinositol 4-phosphate (PI4P), important for the plasma membrane identity and myelin development, as well as remyelination [43].

Besides peptides, low molecular mass compounds related to plasmatic membrane organization for neuritogenesis were identified in our *Cq* extract. Raloxifene is one of them, and its analogue increases the number of regenerating sciatic nerve fibers in mice. Moreover, authors could observe that the molecule increased the axonal transport [44].

A neuroprotective action of 17*β*-estradiol was identified by Ishihara et al. [45] on injured neurons induced by several pathological conditions and by toxic compounds, such as organometallics. This protection has been related to the action of 17*β*-estradiol promoting neurite extension ex vivo and protecting neurons from oxidative stress in vitro. Here, we have found an estradiol-like molecule, the 4-hydroxy-17beta-estradiol-2-S-glutathione that can act in this mechanism [46].

Regarding oxidative stress, antioxidant molecules were also found in the *Cq* extract—several compounds similar to flavonoidsa nd phenolic substances from plants with known antioxidant activity [47]. We have identified kuwanon J and 6-[(2-(Kumar and Khanum 2012)-7-hydroxy-4-oxo-3,4-dihydro-2H-1-benzopyran-5-yl)oxy]-3,4,5-trihydroxyoxane-2-carboxylic acid. These phytochemicals have been associated to neuroprotective activity [48].

Two derivatives of serotonin, (E, E)-4,4^″^-Bi (N-4-hydroxycinnamoylserotonin) and 5-hydroxyindoleacetaldehyde, were also observed. The hydroxycinnamoylserotonin belongs to the n-acylserotonin group. This molecule is frequently found in the central nervous system, synthesized in the mammalian pineal gland and retina, and has demonstrated antioxidant properties, with potential use for protection in neurological disorders, such as Alzheimer's disease, Parkinsonism, and age-related macular degeneration. Moreover, the acylserotonins seem to protect injured neurons against excitotoxic compounds by activating TrkB receptors [49].

Serotonin (5-HT) is an important neurotransmitter that regulates neuronal connectivity during mammalian development and has been associated with neuronal plasticity by promoting secondary neurite outgrowth through 5-HT1A and 5-HT7 receptors [50]. Here, we reported the increase in the number of outgrowths attached to the cell body and branches and junctions of all the processes connected to the cell, indicating primary and secondary neurite formation and elongation. So, the presence of molecules related to 5-HT may contribute to this kind of neuritogenesis.

Regarding a neurotransmitter, N-methyl-a-aminoisobutyric acid and 5-aminopentanoic acid are molecules that act as gamma-aminobutyric acid (GABA). This neurotransmitter is involved in the neuronal protection and survival, besides synapse recovery that contribute to the neuron regenerative process, although the mechanism is not fully understood [33].

Another important amino acid for the neurodegeneration is arginine, and norvaline is a noncompetitive arginase inhibitor that reduced the arginine loss in the brain. This molecule is considered a candidate for Alzheimer's disease treatment, as it has improved the memory and increased proteins related to neuroplasticity [51]. Moreover, the indoleacetic acid, also found in this study, was identified in the cerebrospinal fluid from epileptic patients, related to the tryptamine metabolism [52].

The use of N-acetylgalactosaminyl lactose has been suggested by Vankar and Schmidt [53] for the application on Parkinson's disease. This molecule is a carbohydrate present in the central nervous system, overall. Studies pointed out that these molecules are neuroprotective and increase the regeneration of neurons.

## 5. Conclusions

Considering that neuronal network regeneration is critical for the neuronal regeneration and studies have shown that it is fundamental for memory recovery on Alzheimer's disease and others [54], our study demonstrated that *Cq* extract presented important molecules that, acting with synergy, increased the neurites length and can be able to recover the neuronal connection, which is useful for neurodegenerative diseases.

## Figures and Tables

**Table 1:** Peptides identified in the *C. quadrumanus* methanolic extract.

Peptide sequence	*m*/*z*	*z*	Mass	ALC (%)
FCEHW	721.3490	1	720.2690	76
YQGFAGKSS	472.7750	2	943.4399	59
CPKKDEVP	458.2280	2	914.4531	59
PAYGARF	391.1820	2	780.3918	59
PVGAAPAASVLF	550.3570	2	1098.6072	54
DMYEFAQCA	539.1842	2	1076.3943	54
GSKTFTL	377.2400	2	752.4069	52
GAVVLKGAALKT	564.3934	2	1126.7073	53
KGPVTAASPLGHL	624.3920	2	1246.7034	51
VAFFYVNA	465.7850	2	929.4647	51
ELVHAASCK	479.2880	2	956.4749	51
KNKNKLVPPPLHLGHR	616.7383	3	1847.1006	50
ALVTGGFNAR	503.3231	2	1004.5403	50

**Table 2:** Peptides found peptides in the *C. quadrumanus* methanolic extract and related proteins, with neuronal function.

Sequence	Protein (organism)	Score	Identity (%)	GAPs (%)
FCEHW	Translin-like [(Pocillopora damicornis)	43	80	0
GAVVLKGAALKT	Hyccin-like protein	46	100	0
KNKNKLVPPPLHLGHR	Cyclin-dependent kinase 18-like	59	67	0
	Tyrosine-protein kinase ABL1-like	54	89	0

**Table 3:** LC-MS fingerprinting of low molecular mass compounds related to neuritogenesis, identified in the *C. quadrumanus* methanolic extract.

Compound name	HMDB ID	Formula	*m*/*z*	Score
4-Hydroxy-17beta-estradiol-2-S-glutathione	HMDB0060139	C28H39N3O9S	616.2241	31.4
5-Aminopentanoic acid	HMDB0003355	C5H11NO2	118.0890	34.3
5-Hydroxyindoleacetaldehyde	HMDB0004073	C10H9NO2	140.0572	30.8
5-Methyltetrahydrofolic acid	HMDB0001396	C20H25N7O6	482.1733	34.2
6-[(2-{2-[2-(3,3-Dimethyloxiran-2-yl)ethyl]-7-hydroxy-2-methyl-2H-chromen-6-yl}-7-hydroxy-4-oxo-3,4-dihydro-2H-1-benzopyran-5-yl)oxy]-3,4,5-trihydroxyoxane-2-carboxylic acid	HMDB0132837	C31H34O13	615.2183	30.2
(E,E)-4,4^″^-Bi(N-4-hydroxycinnamoylserotonin)	HMDB0041431	C38H34N4O6	643.2291	30.3
Folinic acid	HMDB0001562	C20H23N7O7	496.1463	31.7
Indoleacetic acid	HMDB0000197	C10H9NO2	140.0572	30.8
Kuwanon J	HMDB0030112	C40H38O10	643.2291	35.5
N-Acetylgalactosaminyl lactose	HMDB0041622	C20H35NO16	510.1827	37.5
N-Methyl-a-aminoisobutyric acid	HMDB0002141	C5H11NO2	118.0890	34.3
Norvaline	HMDB0013716	C5H11NO2	118.0890	34.3
Pteroyl-D-glutamic acid	HMDB0002140	C20H23N7O7	496.1463	31.7
Raloxifene	HMDB0014624	C28H27NO4S	496.1463	30.1

## Data Availability

Supplementary files are available in this submission.

## Supplementary Materials

Supplementary materials Supplementary table: complete list of low molecular mass compounds identified in the *C. quadrumanus* methanolic extract.
