# Supplementary material for: Box Jellyfish (Cnidaria, Cubozoa) Extract Increases Neuron's Connection: A Possible Neuroprotector Effect
Source: Biomed Res Int. 2021 Mar 4;2021:8855248. doi: 10.1155/2021/8855248 (PMC7954621; doi:10.1155/2021/8855248)
Supplement: Supplementary materials — Supplementary table: complete list of low molecular mass compounds identified in the C. quadrumanus methanolic extract. [file 8855248.f1.pdf]

Compound	Compound ID	Adducts	Formula	Score
31.82_510.1827m/z	HMDB0038329	M+H	C20H31NO14	37.6
31.82_510.1827m/z	HMDB0038329	M+H	C20H31NO14	37.6
31.82_510.1827m/z	HMDB0006592	M+H-2H2O	C20H35NO16	37.5
31.82_510.1827m/z	HMDB0039750	M+H-2H2O	C20H35NO16	37.5
31.82_510.1827m/z	HMDB0041622	M+H-2H2O	C20H35NO16	37.5
31.82_510.1827m/z	HMDB0006592	M+H-2H2O	C20H35NO16	37.5
31.82_510.1827m/z	HMDB0039750	M+H-2H2O	C20H35NO16	37.5
31.82_510.1827m/z	HMDB0041622	M+H-2H2O	C20H35NO16	37.5
7.83_437.0684m/z	HMDB0128725	M+Na	C17H18O12	35.8
7.83_437.0684m/z	HMDB0128726	M+Na	C17H18O12	35.8
7.83_437.0684m/z	HMDB0128727	M+Na	C17H18O12	35.8
7.83_437.0684m/z	HMDB0128728	M+Na	C17H18O12	35.8
7.83_437.0684m/z	HMDB0128725	M+Na	C17H18O12	35.8
7.83_437.0684m/z	HMDB0128726	M+Na	C17H18O12	35.8
7.83_437.0684m/z	HMDB0128727	M+Na	C17H18O12	35.8
7.83_437.0684m/z	HMDB0128728	M+Na	C17H18O12	35.8
7.83_437.0684m/z	HMDB0000797	M+H-H2O	C13H19N4O12P	35.6
7.83_437.0684m/z	HMDB0000797	M+H-H2O	C13H19N4O12P	35.6
42.79_643.2291m/z	HMDB0030112	M+H-2H2O	C40H38O10	35.5
42.79_643.2291m/z	HMDB0035964	M+H-2H2O	C40H38O10	35.5
42.79_643.2291m/z	HMDB0126375	M+H-2H2O	C40H38O10	35.5
38.04_341.1982m/z	HMDB0015075	M+H	C21H26NO3+	35.5
38.04_341.1982m/z	HMDB0015591	M+H	C21H26NO3+	35.5
42.79_643.2291m/z	HMDB0030112	M+H-2H2O	C40H38O10	35.5
42.79_643.2291m/z	HMDB0035964	M+H-2H2O	C40H38O10	35.5
42.79_643.2291m/z	HMDB0126375	M+H-2H2O	C40H38O10	35.5
38.04_341.1982m/z	HMDB0015075	M+H	C21H26NO3+	35.5
38.04_341.1982m/z	HMDB0015591	M+H	C21H26NO3+	35.5
39.00_615.2230m/z	HMDB0006607	M+H-H2O	C23H40N2O18	35.4
39.00_615.2230m/z	HMDB0041621	M+H-H2O	C23H40N2O18	35.4
39.00_615.2230m/z	HMDB0006607	M+H-H2O	C23H40N2O18	35.4
39.00_615.2230m/z	HMDB0041621	M+H-H2O	C23H40N2O18	35.4
38.04_341.1982m/z	HMDB0014876	M+H	C19H24N4O2	35.3
38.04_341.1982m/z	HMDB0014876	M+H	C19H24N4O2	35.3
38.04_341.1982m/z	HMDB0015121	M+Na	C19H28NO3+	34.6
38.04_341.1982m/z	HMDB0015121	M+Na	C19H28NO3+	34.6
2.33_118.0890m/z	HMDB0000043	M+H	C5H11NO2	34.3
2.33_118.0890m/z	HMDB0000883	M+H	C5H11NO2	34.3
2.33_118.0890m/z	HMDB0001382	M+H	C5H11NO2	34.3
2.33_118.0890m/z	HMDB0002141	M+H	C5H11NO2	34.3
2.33_118.0890m/z	HMDB0003355	M+H	C5H11NO2	34.3
2.33_118.0890m/z	HMDB0013716	M+H	C5H11NO2	34.3
2.33_118.0890m/z	HMDB0015550	M+H	C5H11NO2	34.3
2.33_118.0890m/z	HMDB0000043	M+H	C5H11NO2	34.3
2.33_118.0890m/z	HMDB0000883	M+H	C5H11NO2	34.3
2.33_118.0890m/z	HMDB0001382	M+H	C5H11NO2	34.3
28.39_482.1733m/z	HMDB0001396	M+Na	C20H25N7O6	34.2
31.82_510.1827m/z	HMDB0014832	M+H-H2O	C27H29NO10	34.2

31.82_510.1827m/z	HMDB0041884	M+H-2H2O	C27H31NO11	34.2
31.82_510.1827m/z	HMDB0060823	M+H-2H2O	C27H31NO11	34.2
39.04_490.2726m/z	HMDB0011496	M+H-2H2O	C27H44NO7P	34.1
39.04_490.2726m/z	HMDB0011526	M+H-2H2O	C27H44NO7P	34.1
39.04_490.2726m/z	HMDB0011496	M+H-2H2O	C27H44NO7P	34.1
39.04_490.2726m/z	HMDB0011526	M+H-2H2O	C27H44NO7P	34.1
42.79_643.2291m/z	HMDB0130483	M+H-2H2O	C36H38O13	33.8
42.79_643.2291m/z	HMDB0130484	M+H-2H2O	C36H38O13	33.8
42.79_643.2291m/z	HMDB0130485	M+H-2H2O	C36H38O13	33.8
27.53_496.1463m/z	HMDB0029776	M+Na	C20H27NO12	33.8
42.79_643.2291m/z	HMDB0130483	M+H-2H2O	C36H38O13	33.8
42.79_643.2291m/z	HMDB0130484	M+H-2H2O	C36H38O13	33.8
42.79_643.2291m/z	HMDB0130485	M+H-2H2O	C36H38O13	33.8
27.53_496.1463m/z	HMDB0029776	M+Na	C20H27NO12	33.8
31.82_510.1827m/z	HMDB0132953	M+H-H2O	C23H33N3O9S	33.6
31.82_510.1827m/z	HMDB0132954	M+H-H2O	C23H33N3O9S	33.6
31.82_510.1827m/z	HMDB0132953	M+H-H2O	C23H33N3O9S	33.6
31.82_510.1827m/z	HMDB0132954	M+H-H2O	C23H33N3O9S	33.6
28.39_482.1733m/z	HMDB0015230	M+H	C22H28FN3O6S	33.5
38.04_341.1982m/z	HMDB0029850	M+Na	C16H30O6	33.5
38.04_341.1982m/z	HMDB0032368	M+Na	C16H30O6	33.5
43.72_752.1608m/z	HMDB0001197	M+H-2H2O	C27H35N9O15P2	33.3
40.74_629.2282m/z	HMDB0032114	M+H-H2O	C38H34N2O8	32.9
38.94_615.2186m/z	HMDB0006607	M+H-H2O	C23H40N2O18	32.8
38.94_615.2186m/z	HMDB0041621	M+H-H2O	C23H40N2O18	32.8
44.45_779.1615m/z	HMDB0126885	M+Na	C32H36O21	32.6
44.45_779.1615m/z	HMDB0126886	M+Na	C32H36O21	32.6
44.45_779.1615m/z	HMDB0126887	M+Na	C32H36O21	32.6
44.45_779.1615m/z	HMDB0126888	M+Na	C32H36O21	32.6
44.45_779.1615m/z	HMDB0127450	M+Na	C32H36O21	32.6
44.45_779.1615m/z	HMDB0127451	M+Na	C32H36O21	32.6
44.45_779.1615m/z	HMDB0126885	M+Na	C32H36O21	32.6
44.45_779.1615m/z	HMDB0126886	M+Na	C32H36O21	32.6
44.45_779.1615m/z	HMDB0126887	M+Na	C32H36O21	32.6
44.45_779.1615m/z	HMDB0126888	M+Na	C32H36O21	32.6
44.45_779.1615m/z	HMDB0127450	M+Na	C32H36O21	32.6
44.45_779.1615m/z	HMDB0127451	M+Na	C32H36O21	32.6
44.45_779.1615m/z	HMDB0127452	M+Na	C32H36O21	32.6
7.83_437.0684m/z	HMDB0060316	M+Na	C16H18N2O9S	32.5
39.04_615.2607m/z	HMDB0129666	M+H-2H2O	C36H42O11	32.5
39.04_615.2607m/z	HMDB0129667	M+H-2H2O	C36H42O11	32.5
39.04_615.2607m/z	HMDB0129668	M+H-2H2O	C36H42O11	32.5
7.83_437.0684m/z	HMDB0060316	M+Na	C16H18N2O9S	32.5
39.04_615.2607m/z	HMDB0129666	M+H-2H2O	C36H42O11	32.5
39.04_615.2607m/z	HMDB0129667	M+H-2H2O	C36H42O11	32.5
39.04_615.2607m/z	HMDB0129668	M+H-2H2O	C36H42O11	32.5
35.38_538.2029m/z	HMDB0006595	M+Na	C20H37NO14	32.4
40.66_629.2270m/z	HMDB0032114	M+H-H2O	C38H34N2O8	32.4
40.66_629.2270m/z	HMDB0034634	M+H-H2O	C32H38O14	32.2
43.72_752.1608m/z	HMDB0125188	M+H-2H2O	C36H35O20+	32.1

43.72_752.1608m/z	HMDB0125189	M+H-2H2O	C36H35O20+	32.1
43.72_752.1608m/z	HMDB0125190	M+H-2H2O	C36H35O20+	32.1
43.72_752.1608m/z	HMDB0125191	M+H-2H2O	C36H35O20+	32.1
43.72_752.1608m/z	HMDB0125192	M+H-2H2O	C36H35O20+	32.1
43.72_752.1608m/z	HMDB0125193	M+H-2H2O	C36H35O20+	32.1
43.72_752.1608m/z	HMDB0125188	M+H-2H2O	C36H35O20+	32.1
43.72_752.1608m/z	HMDB0125189	M+H-2H2O	C36H35O20+	32.1
43.72_752.1608m/z	HMDB0125190	M+H-2H2O	C36H35O20+	32.1
43.72_752.1608m/z	HMDB0125191	M+H-2H2O	C36H35O20+	32.1
43.72_752.1608m/z	HMDB0125192	M+H-2H2O	C36H35O20+	32.1
43.72_752.1608m/z	HMDB0125193	M+H-2H2O	C36H35O20+	32.1
40.74_629.2282m/z	HMDB0034634	M+H-H2O	C32H38O14	31.8
38.04_341.1982m/z	HMDB0014469	M+H-2H2O	C22H29FO4	31.8
38.04_341.1982m/z	HMDB0014687	M+H-2H2O	C22H29FO4	31.8
0.29_156.0199m/z	HMDB0033843	M+H-H2O	C6H9NOP2	31.8
40.74_629.2282m/z	HMDB0034634	M+H-H2O	C32H38O14	31.8
44.45_779.1615m/z	HMDB0041730	M+H-H2O	C34H36O22	31.7
27.53_496.1463m/z	HMDB0000972	M+Na	C20H23N7O7	31.7
27.53_496.1463m/z	HMDB0001562	M+Na	C20H23N7O7	31.7
27.53_496.1463m/z	HMDB0002140	M+Na	C20H23N7O7	31.7
27.53_496.1463m/z	HMDB0062757	M+Na	C20H23N7O7	31.7
44.45_779.1615m/z	HMDB0037373	M+Na	C39H32O16	31.6
27.53_496.1463m/z	HMDB0128696	M+H-2H2O	C21H29N3O11S	31.5
38.98_616.2241m/z	HMDB0060139	M+Na	C28H39N3O9S	31.4
38.04_341.1982m/z	HMDB0060574	M+H	C17H28N2O5	31.4
27.53_496.1463m/z	HMDB0031112	M+H-2H2O	C29H25NO9	31.4
27.53_496.1463m/z	HMDB0034788	M+H-2H2O	C29H25NO9	31.4
27.53_496.1463m/z	HMDB0040769	M+H-H2O	C29H23NO8	31.3
27.53_496.1463m/z	HMDB0040769	M+H-H2O	C29H23NO8	31.3
39.28_566.2113m/z	HMDB0040387	M+H	C34H31NO7	31.2
0.29_156.0199m/z	HMDB0000191	M+Na	C4H7NO4	31.2
0.29_156.0199m/z	HMDB0006483	M+Na	C4H7NO4	31.2
0.29_156.0199m/z	HMDB0011753	M+Na	C4H7NO4	31.2
0.29_156.0199m/z	HMDB0062186	M+Na	C4H7NO4	31.2
0.29_156.0199m/z	HMDB0062501	M+Na	C4H7NO4	31.2
39.28_566.2113m/z	HMDB0040387	M+H	C34H31NO7	31.2
28.39_482.1733m/z	HMDB0015481	M+H	C27H23N5O4	30.8
0.28_140.0572m/z	HMDB0000197	M+H-2H2O	C10H9NO2	30.8
0.28_140.0572m/z	HMDB0004073	M+H-2H2O	C10H9NO2	30.8
0.28_140.0572m/z	HMDB0038628	M+H-2H2O	C10H9NO2	30.8
0.28_140.0572m/z	HMDB0040972	M+H-2H2O	C10H9NO2	30.8
28.39_482.1733m/z	HMDB0015481	M+H	C27H23N5O4	30.8
0.28_140.0572m/z	HMDB0000197	M+H-2H2O	C10H9NO2	30.8
0.28_140.0572m/z	HMDB0004073	M+H-2H2O	C10H9NO2	30.8
0.28_140.0572m/z	HMDB0038628	M+H-2H2O	C10H9NO2	30.8
0.28_140.0572m/z	HMDB0040972	M+H-2H2O	C10H9NO2	30.8
35.38_538.2029m/z	HMDB0034773	M+H	C25H31NO12	30.7
35.38_538.2029m/z	HMDB0034774	M+H	C25H31NO12	30.7
43.01_643.2387m/z	HMDB0030112	M+H-2H2O	C40H38O10	30.7
43.01_643.2387m/z	HMDB0035964	M+H-2H2O	C40H38O10	30.7

43.01_643.2387m/z	HMDB0126375	M+H-2H2O	C40H38O10	30.7
27.53_496.1463m/z	HMDB0134078	M+Na	C19H27N3O9S	30.7
35.38_538.2029m/z	HMDB0034773	M+H	C25H31NO12	30.7
35.38_538.2029m/z	HMDB0034774	M+H	C25H31NO12	30.7
43.01_643.2387m/z	HMDB0030112	M+H-2H2O	C40H38O10	30.7
43.01_643.2387m/z	HMDB0035964	M+H-2H2O	C40H38O10	30.7
43.01_643.2387m/z	HMDB0126375	M+H-2H2O	C40H38O10	30.7
27.53_496.1463m/z	HMDB0134078	M+Na	C19H27N3O9S	30.7
38.73_615.2183m/z	HMDB0030627	M+H-2H2O	C31H38O15	30.3
38.73_615.2183m/z	HMDB0034281	M+H-2H2O	C31H38O15	30.3
39.28_566.2113m/z	HMDB0135503	M+H	C27H35NO12	30.3
39.28_566.2113m/z	HMDB0135505	M+H	C27H35NO12	30.3
39.28_566.2113m/z	HMDB0135507	M+H	C27H35NO12	30.3
42.79_643.2291m/z	HMDB0041431	M+H	C38H34N4O6	30.3
0.28_140.0572m/z	HMDB0062189	M+H-H2O	C10H7NO	30.3
38.73_615.2183m/z	HMDB0030627	M+H-2H2O	C31H38O15	30.3
38.73_615.2183m/z	HMDB0034281	M+H-2H2O	C31H38O15	30.3
39.28_566.2113m/z	HMDB0135503	M+H	C27H35NO12	30.3
39.28_566.2113m/z	HMDB0135505	M+H	C27H35NO12	30.3
39.28_566.2113m/z	HMDB0135507	M+H	C27H35NO12	30.3
42.79_643.2291m/z	HMDB0041431	M+H	C38H34N4O6	30.3
0.28_140.0572m/z	HMDB0062189	M+H-H2O	C10H7NO	30.3
38.73_615.2183m/z	HMDB0129415	M+H	C31H34O13	30.2
38.73_615.2183m/z	HMDB0129416	M+H	C31H34O13	30.2
38.73_615.2183m/z	HMDB0129417	M+H	C31H34O13	30.2
38.73_615.2183m/z	HMDB0132767	M+H	C31H34O13	30.2
38.73_615.2183m/z	HMDB0132768	M+H	C31H34O13	30.2
38.73_615.2183m/z	HMDB0132770	M+H	C31H34O13	30.2
38.73_615.2183m/z	HMDB0132771	M+H	C31H34O13	30.2
38.73_615.2183m/z	HMDB0132772	M+H	C31H34O13	30.2
38.73_615.2183m/z	HMDB0132774	M+H	C31H34O13	30.2
38.73_615.2183m/z	HMDB0132775	M+H	C31H34O13	30.2
38.73_615.2183m/z	HMDB0132776	M+H	C31H34O13	30.2
38.73_615.2183m/z	HMDB0132777	M+H	C31H34O13	30.2
38.73_615.2183m/z	HMDB0132778	M+H	C31H34O13	30.2
38.73_615.2183m/z	HMDB0132779	M+H	C31H34O13	30.2
38.73_615.2183m/z	HMDB0132780	M+H	C31H34O13	30.2
38.73_615.2183m/z	HMDB0132782	M+H	C31H34O13	30.2
38.73_615.2183m/z	HMDB0132783	M+H	C31H34O13	30.2
38.73_615.2183m/z	HMDB0132784	M+H	C31H34O13	30.2
38.73_615.2183m/z	HMDB0132786	M+H	C31H34O13	30.2
38.73_615.2183m/z	HMDB0132787	M+H	C31H34O13	30.2
38.73_615.2183m/z	HMDB0132788	M+H	C31H34O13	30.2
38.73_615.2183m/z	HMDB0132789	M+H	C31H34O13	30.2
38.73_615.2183m/z	HMDB0132791	M+H	C31H34O13	30.2
38.73_615.2183m/z	HMDB0132792	M+H	C31H34O13	30.2
38.73_615.2183m/z	HMDB0132793	M+H	C31H34O13	30.2
38.73_615.2183m/z	HMDB0132811	M+H	C31H34O13	30.2
38.73_615.2183m/z	HMDB0132812	M+H	C31H34O13	30.2
38.73_615.2183m/z	HMDB0132813	M+H	C31H34O13	30.2

38.73_615.2183m/z	HMDB0132815	M+H	C31H34O13	30.2
38.73_615.2183m/z	HMDB0132816	M+H	C31H34O13	30.2
38.73_615.2183m/z	HMDB0132817	M+H	C31H34O13	30.2
38.73_615.2183m/z	HMDB0132819	M+H	C31H34O13	30.2
38.73_615.2183m/z	HMDB0132820	M+H	C31H34O13	30.2
38.73_615.2183m/z	HMDB0132821	M+H	C31H34O13	30.2
38.73_615.2183m/z	HMDB0132822	M+H	C31H34O13	30.2
38.73_615.2183m/z	HMDB0132823	M+H	C31H34O13	30.2
38.73_615.2183m/z	HMDB0132824	M+H	C31H34O13	30.2
38.73_615.2183m/z	HMDB0132825	M+H	C31H34O13	30.2
38.73_615.2183m/z	HMDB0132828	M+H	C31H34O13	30.2
38.73_615.2183m/z	HMDB0132831	M+H	C31H34O13	30.2
38.73_615.2183m/z	HMDB0132832	M+H	C31H34O13	30.2
38.73_615.2183m/z	HMDB0132833	M+H	C31H34O13	30.2
38.73_615.2183m/z	HMDB0132835	M+H	C31H34O13	30.2
38.73_615.2183m/z	HMDB0132836	M+H	C31H34O13	30.2
38.73_615.2183m/z	HMDB0132837	M+H	C31H34O13	30.2
38.73_615.2183m/z	HMDB0132838	M+H	C31H34O13	30.2
38.73_615.2183m/z	HMDB0132839	M+H	C31H34O13	30.2
38.73_615.2183m/z	HMDB0132840	M+H	C31H34O13	30.2
38.73_615.2183m/z	HMDB0132841	M+H	C31H34O13	30.2
38.73_615.2183m/z	HMDB0132842	M+H	C31H34O13	30.2
38.73_615.2183m/z	HMDB0132843	M+H	C31H34O13	30.2
38.73_615.2183m/z	HMDB0132844	M+H	C31H34O13	30.2
38.73_615.2183m/z	HMDB0132845	M+H	C31H34O13	30.2
38.73_615.2183m/z	HMDB0132846	M+H	C31H34O13	30.2
38.73_615.2183m/z	HMDB0132847	M+H	C31H34O13	30.2
38.73_615.2183m/z	HMDB0132848	M+H	C31H34O13	30.2
40.74_629.2282m/z	HMDB0031149	M+Na	C35H34N4O6	30.2
39.00_615.2230m/z	HMDB0030627	M+H-2H2O	C31H38O15	30.2
39.00_615.2230m/z	HMDB0034281	M+H-2H2O	C31H38O15	30.2
38.94_615.2186m/z	HMDB0030627	M+H-2H2O	C31H38O15	30.2
38.94_615.2186m/z	HMDB0034281	M+H-2H2O	C31H38O15	30.2
38.04_341.1982m/z	HMDB0001337	M+Na	C20H30O3	30.2
38.04_341.1982m/z	HMDB0005081	M+Na	C20H30O3	30.2
38.04_341.1982m/z	HMDB0006027	M+Na	C20H30O3	30.2
38.04_341.1982m/z	HMDB0010202	M+Na	C20H30O3	30.2
38.04_341.1982m/z	HMDB0010205	M+Na	C20H30O3	30.2
38.04_341.1982m/z	HMDB0010209	M+Na	C20H30O3	30.2
38.04_341.1982m/z	HMDB0010210	M+Na	C20H30O3	30.2
38.04_341.1982m/z	HMDB0010212	M+Na	C20H30O3	30.2
38.04_341.1982m/z	HMDB0010217	M+Na	C20H30O3	30.2
38.04_341.1982m/z	HMDB0012534	M+Na	C20H30O3	30.2
38.04_341.1982m/z	HMDB0012611	M+Na	C20H30O3	30.2
38.04_341.1982m/z	HMDB0013633	M+Na	C20H30O3	30.2
38.04_341.1982m/z	HMDB0030101	M+Na	C20H30O3	30.2
38.04_341.1982m/z	HMDB0035116	M+Na	C20H30O3	30.2
38.04_341.1982m/z	HMDB0035311	M+Na	C20H30O3	30.2
38.04_341.1982m/z	HMDB0036698	M+Na	C20H30O3	30.2
38.04_341.1982m/z	HMDB0036706	M+Na	C20H30O3	30.2

38.04_341.1982m/z	HMDB0036712	M+Na	C20H30O3	30.2
38.04_341.1982m/z	HMDB0036713	M+Na	C20H30O3	30.2
38.04_341.1982m/z	HMDB0036724	M+Na	C20H30O3	30.2
38.04_341.1982m/z	HMDB0036752	M+Na	C20H30O3	30.2
38.04_341.1982m/z	HMDB0036776	M+Na	C20H30O3	30.2
38.04_341.1982m/z	HMDB0036806	M+Na	C20H30O3	30.2
38.04_341.1982m/z	HMDB0036839	M+Na	C20H30O3	30.2
38.04_341.1982m/z	HMDB0036930	M+Na	C20H30O3	30.2
38.04_341.1982m/z	HMDB0037523	M+Na	C20H30O3	30.2
38.04_341.1982m/z	HMDB0038521	M+Na	C20H30O3	30.2
38.04_341.1982m/z	HMDB0039012	M+Na	C20H30O3	30.2
38.04_341.1982m/z	HMDB0039716	M+Na	C20H30O3	30.2
38.04_341.1982m/z	HMDB0041058	M+Na	C20H30O3	30.2
38.04_341.1982m/z	HMDB0060053	M+Na	C20H30O3	30.2
38.04_341.1982m/z	HMDB0061114	M+Na	C20H30O3	30.2
38.04_341.1982m/z	HMDB0062222	M+Na	C20H30O3	30.2
38.04_341.1982m/z	HMDB0062293	M+Na	C20H30O3	30.2
38.04_341.1982m/z	HMDB0062296	M+Na	C20H30O3	30.2
38.04_341.1982m/z	HMDB0062408	M+Na	C20H30O3	30.2
38.04_341.1982m/z	HMDB0137419	M+Na	C20H30O3	30.2
38.73_615.2183m/z	HMDB0129415	M+H	C31H34O13	30.2
38.73_615.2183m/z	HMDB0129416	M+H	C31H34O13	30.2
38.73_615.2183m/z	HMDB0129417	M+H	C31H34O13	30.2
38.73_615.2183m/z	HMDB0132767	M+H	C31H34O13	30.2
38.73_615.2183m/z	HMDB0132768	M+H	C31H34O13	30.2
38.73_615.2183m/z	HMDB0132770	M+H	C31H34O13	30.2
38.73_615.2183m/z	HMDB0132771	M+H	C31H34O13	30.2
38.73_615.2183m/z	HMDB0132772	M+H	C31H34O13	30.2
38.73_615.2183m/z	HMDB0132774	M+H	C31H34O13	30.2
38.73_615.2183m/z	HMDB0132775	M+H	C31H34O13	30.2
38.73_615.2183m/z	HMDB0132776	M+H	C31H34O13	30.2
38.73_615.2183m/z	HMDB0132777	M+H	C31H34O13	30.2
38.73_615.2183m/z	HMDB0132778	M+H	C31H34O13	30.2
38.73_615.2183m/z	HMDB0132779	M+H	C31H34O13	30.2
38.73_615.2183m/z	HMDB0132780	M+H	C31H34O13	30.2
38.73_615.2183m/z	HMDB0132782	M+H	C31H34O13	30.2
38.73_615.2183m/z	HMDB0132783	M+H	C31H34O13	30.2
38.73_615.2183m/z	HMDB0132784	M+H	C31H34O13	30.2
38.73_615.2183m/z	HMDB0132786	M+H	C31H34O13	30.2
38.73_615.2183m/z	HMDB0132787	M+H	C31H34O13	30.2
38.73_615.2183m/z	HMDB0132788	M+H	C31H34O13	30.2
38.73_615.2183m/z	HMDB0132789	M+H	C31H34O13	30.2
38.73_615.2183m/z	HMDB0132791	M+H	C31H34O13	30.2
38.73_615.2183m/z	HMDB0132792	M+H	C31H34O13	30.2
38.73_615.2183m/z	HMDB0132793	M+H	C31H34O13	30.2
38.73_615.2183m/z	HMDB0132811	M+H	C31H34O13	30.2
38.73_615.2183m/z	HMDB0132812	M+H	C31H34O13	30.2
38.73_615.2183m/z	HMDB0132813	M+H	C31H34O13	30.2
38.73_615.2183m/z	HMDB0132815	M+H	C31H34O13	30.2
38.73_615.2183m/z	HMDB0132816	M+H	C31H34O13	30.2

38.73_615.2183m/z	HMDB0132817	M+H	C31H34O13	30.2
38.73_615.2183m/z	HMDB0132819	M+H	C31H34O13	30.2
38.73_615.2183m/z	HMDB0132820	M+H	C31H34O13	30.2
38.73_615.2183m/z	HMDB0132821	M+H	C31H34O13	30.2
38.73_615.2183m/z	HMDB0132822	M+H	C31H34O13	30.2
38.73_615.2183m/z	HMDB0132823	M+H	C31H34O13	30.2
38.73_615.2183m/z	HMDB0132824	M+H	C31H34O13	30.2
38.73_615.2183m/z	HMDB0132825	M+H	C31H34O13	30.2
38.73_615.2183m/z	HMDB0132828	M+H	C31H34O13	30.2
38.73_615.2183m/z	HMDB0132831	M+H	C31H34O13	30.2
38.73_615.2183m/z	HMDB0132832	M+H	C31H34O13	30.2
38.73_615.2183m/z	HMDB0132833	M+H	C31H34O13	30.2
38.73_615.2183m/z	HMDB0132835	M+H	C31H34O13	30.2
38.73_615.2183m/z	HMDB0132836	M+H	C31H34O13	30.2
38.73_615.2183m/z	HMDB0132837	M+H	C31H34O13	30.2
38.73_615.2183m/z	HMDB0132838	M+H	C31H34O13	30.2
38.73_615.2183m/z	HMDB0132839	M+H	C31H34O13	30.2
38.73_615.2183m/z	HMDB0132840	M+H	C31H34O13	30.2
38.73_615.2183m/z	HMDB0132841	M+H	C31H34O13	30.2
38.73_615.2183m/z	HMDB0132842	M+H	C31H34O13	30.2
38.73_615.2183m/z	HMDB0132843	M+H	C31H34O13	30.2
38.73_615.2183m/z	HMDB0132844	M+H	C31H34O13	30.2
38.73_615.2183m/z	HMDB0132845	M+H	C31H34O13	30.2
38.73_615.2183m/z	HMDB0132846	M+H	C31H34O13	30.2
38.73_615.2183m/z	HMDB0132847	M+H	C31H34O13	30.2
38.73_615.2183m/z	HMDB0132848	M+H	C31H34O13	30.2
40.74_629.2282m/z	HMDB0031149	M+Na	C35H34N4O6	30.2
39.00_615.2230m/z	HMDB0030627	M+H-2H2O	C31H38O15	30.2
39.00_615.2230m/z	HMDB0034281	M+H-2H2O	C31H38O15	30.2
38.94_615.2186m/z	HMDB0030627	M+H-2H2O	C31H38O15	30.2
38.94_615.2186m/z	HMDB0034281	M+H-2H2O	C31H38O15	30.2
38.04_341.1982m/z	HMDB0001337	M+Na	C20H30O3	30.2
38.04_341.1982m/z	HMDB0005081	M+Na	C20H30O3	30.2
38.04_341.1982m/z	HMDB0006027	M+Na	C20H30O3	30.2
38.04_341.1982m/z	HMDB0010202	M+Na	C20H30O3	30.2
38.04_341.1982m/z	HMDB0010205	M+Na	C20H30O3	30.2
38.04_341.1982m/z	HMDB0010209	M+Na	C20H30O3	30.2
38.04_341.1982m/z	HMDB0010210	M+Na	C20H30O3	30.2
38.04_341.1982m/z	HMDB0010212	M+Na	C20H30O3	30.2
38.04_341.1982m/z	HMDB0010217	M+Na	C20H30O3	30.2
38.04_341.1982m/z	HMDB0012534	M+Na	C20H30O3	30.2
38.04_341.1982m/z	HMDB0012611	M+Na	C20H30O3	30.2
38.04_341.1982m/z	HMDB0013633	M+Na	C20H30O3	30.2
38.04_341.1982m/z	HMDB0030101	M+Na	C20H30O3	30.2
38.04_341.1982m/z	HMDB0035116	M+Na	C20H30O3	30.2
38.04_341.1982m/z	HMDB0035311	M+Na	C20H30O3	30.2
38.04_341.1982m/z	HMDB0036698	M+Na	C20H30O3	30.2
38.04_341.1982m/z	HMDB0036706	M+Na	C20H30O3	30.2
38.04_341.1982m/z	HMDB0036712	M+Na	C20H30O3	30.2
38.04_341.1982m/z	HMDB0036713	M+Na	C20H30O3	30.2

38.04_341.1982m/z	HMDB0036724	M+Na	C20H30O3	30.2
38.04_341.1982m/z	HMDB0036752	M+Na	C20H30O3	30.2
38.04_341.1982m/z	HMDB0036776	M+Na	C20H30O3	30.2
38.04_341.1982m/z	HMDB0036806	M+Na	C20H30O3	30.2
38.04_341.1982m/z	HMDB0036839	M+Na	C20H30O3	30.2
38.04_341.1982m/z	HMDB0036930	M+Na	C20H30O3	30.2
38.04_341.1982m/z	HMDB0037523	M+Na	C20H30O3	30.2
38.04_341.1982m/z	HMDB0038521	M+Na	C20H30O3	30.2
38.04_341.1982m/z	HMDB0039012	M+Na	C20H30O3	30.2
38.04_341.1982m/z	HMDB0039716	M+Na	C20H30O3	30.2
38.04_341.1982m/z	HMDB0041058	M+Na	C20H30O3	30.2
38.04_341.1982m/z	HMDB0060053	M+Na	C20H30O3	30.2
38.04_341.1982m/z	HMDB0061114	M+Na	C20H30O3	30.2
38.04_341.1982m/z	HMDB0062222	M+Na	C20H30O3	30.2
38.04_341.1982m/z	HMDB0062293	M+Na	C20H30O3	30.2
38.04_341.1982m/z	HMDB0062296	M+Na	C20H30O3	30.2
38.04_341.1982m/z	HMDB0062408	M+Na	C20H30O3	30.2
38.04_341.1982m/z	HMDB0137419	M+Na	C20H30O3	30.2
38.98_616.2241m/z	HMDB0000825	M+H-H2O	C23H39NO19	30.1
38.98_616.2241m/z	HMDB0006569	M+H-H2O	C23H39NO19	30.1
39.00_615.2230m/z	HMDB0129415	M+H	C31H34O13	30.1
39.00_615.2230m/z	HMDB0129416	M+H	C31H34O13	30.1
39.00_615.2230m/z	HMDB0129417	M+H	C31H34O13	30.1
39.00_615.2230m/z	HMDB0132767	M+H	C31H34O13	30.1
39.00_615.2230m/z	HMDB0132768	M+H	C31H34O13	30.1
39.00_615.2230m/z	HMDB0132770	M+H	C31H34O13	30.1
39.00_615.2230m/z	HMDB0132771	M+H	C31H34O13	30.1
39.00_615.2230m/z	HMDB0132772	M+H	C31H34O13	30.1
39.00_615.2230m/z	HMDB0132774	M+H	C31H34O13	30.1
39.00_615.2230m/z	HMDB0132775	M+H	C31H34O13	30.1
39.00_615.2230m/z	HMDB0132776	M+H	C31H34O13	30.1
39.00_615.2230m/z	HMDB0132777	M+H	C31H34O13	30.1
39.00_615.2230m/z	HMDB0132778	M+H	C31H34O13	30.1
39.00_615.2230m/z	HMDB0132779	M+H	C31H34O13	30.1
39.00_615.2230m/z	HMDB0132780	M+H	C31H34O13	30.1
39.00_615.2230m/z	HMDB0132782	M+H	C31H34O13	30.1
39.00_615.2230m/z	HMDB0132783	M+H	C31H34O13	30.1
39.00_615.2230m/z	HMDB0132784	M+H	C31H34O13	30.1
39.00_615.2230m/z	HMDB0132786	M+H	C31H34O13	30.1
39.00_615.2230m/z	HMDB0132787	M+H	C31H34O13	30.1
39.00_615.2230m/z	HMDB0132788	M+H	C31H34O13	30.1
39.00_615.2230m/z	HMDB0132789	M+H	C31H34O13	30.1
39.00_615.2230m/z	HMDB0132791	M+H	C31H34O13	30.1
39.00_615.2230m/z	HMDB0132792	M+H	C31H34O13	30.1
39.00_615.2230m/z	HMDB0132793	M+H	C31H34O13	30.1
39.00_615.2230m/z	HMDB0132811	M+H	C31H34O13	30.1
39.00_615.2230m/z	HMDB0132812	M+H	C31H34O13	30.1
39.00_615.2230m/z	HMDB0132813	M+H	C31H34O13	30.1
39.00_615.2230m/z	HMDB0132815	M+H	C31H34O13	30.1
39.00_615.2230m/z	HMDB0132816	M+H	C31H34O13	30.1

39.00_615.2230m/z	HMDB0132817	M+H	C31H34O13	30.1
39.00_615.2230m/z	HMDB0132819	M+H	C31H34O13	30.1
39.00_615.2230m/z	HMDB0132820	M+H	C31H34O13	30.1
39.00_615.2230m/z	HMDB0132821	M+H	C31H34O13	30.1
39.00_615.2230m/z	HMDB0132822	M+H	C31H34O13	30.1
39.00_615.2230m/z	HMDB0132823	M+H	C31H34O13	30.1
39.00_615.2230m/z	HMDB0132824	M+H	C31H34O13	30.1
39.00_615.2230m/z	HMDB0132825	M+H	C31H34O13	30.1
39.00_615.2230m/z	HMDB0132828	M+H	C31H34O13	30.1
39.00_615.2230m/z	HMDB0132831	M+H	C31H34O13	30.1
39.00_615.2230m/z	HMDB0132832	M+H	C31H34O13	30.1
39.00_615.2230m/z	HMDB0132833	M+H	C31H34O13	30.1
39.00_615.2230m/z	HMDB0132835	M+H	C31H34O13	30.1
39.00_615.2230m/z	HMDB0132836	M+H	C31H34O13	30.1
39.00_615.2230m/z	HMDB0132837	M+H	C31H34O13	30.1
39.00_615.2230m/z	HMDB0132838	M+H	C31H34O13	30.1
39.00_615.2230m/z	HMDB0132839	M+H	C31H34O13	30.1
39.00_615.2230m/z	HMDB0132840	M+H	C31H34O13	30.1
39.00_615.2230m/z	HMDB0132841	M+H	C31H34O13	30.1
39.00_615.2230m/z	HMDB0132842	M+H	C31H34O13	30.1
39.00_615.2230m/z	HMDB0132843	M+H	C31H34O13	30.1
39.00_615.2230m/z	HMDB0132844	M+H	C31H34O13	30.1
39.00_615.2230m/z	HMDB0132845	M+H	C31H34O13	30.1
39.00_615.2230m/z	HMDB0132846	M+H	C31H34O13	30.1
39.00_615.2230m/z	HMDB0132847	M+H	C31H34O13	30.1
39.00_615.2230m/z	HMDB0132848	M+H	C31H34O13	30.1
38.94_615.2186m/z	HMDB0129415	M+H	C31H34O13	30.1
38.94_615.2186m/z	HMDB0129416	M+H	C31H34O13	30.1
38.94_615.2186m/z	HMDB0129417	M+H	C31H34O13	30.1
38.94_615.2186m/z	HMDB0132767	M+H	C31H34O13	30.1
38.94_615.2186m/z	HMDB0132768	M+H	C31H34O13	30.1
38.94_615.2186m/z	HMDB0132770	M+H	C31H34O13	30.1
38.94_615.2186m/z	HMDB0132771	M+H	C31H34O13	30.1
38.94_615.2186m/z	HMDB0132772	M+H	C31H34O13	30.1
38.94_615.2186m/z	HMDB0132774	M+H	C31H34O13	30.1
38.94_615.2186m/z	HMDB0132775	M+H	C31H34O13	30.1
38.94_615.2186m/z	HMDB0132776	M+H	C31H34O13	30.1
38.94_615.2186m/z	HMDB0132777	M+H	C31H34O13	30.1
38.94_615.2186m/z	HMDB0132778	M+H	C31H34O13	30.1
38.94_615.2186m/z	HMDB0132779	M+H	C31H34O13	30.1
38.94_615.2186m/z	HMDB0132780	M+H	C31H34O13	30.1
38.94_615.2186m/z	HMDB0132782	M+H	C31H34O13	30.1
38.94_615.2186m/z	HMDB0132783	M+H	C31H34O13	30.1
38.94_615.2186m/z	HMDB0132784	M+H	C31H34O13	30.1
38.94_615.2186m/z	HMDB0132786	M+H	C31H34O13	30.1
38.94_615.2186m/z	HMDB0132787	M+H	C31H34O13	30.1
38.94_615.2186m/z	HMDB0132788	M+H	C31H34O13	30.1
38.94_615.2186m/z	HMDB0132789	M+H	C31H34O13	30.1
38.94_615.2186m/z	HMDB0132791	M+H	C31H34O13	30.1
38.94_615.2186m/z	HMDB0132792	M+H	C31H34O13	30.1

38.94_615.2186m/z	HMDB0132793	M+H	C31H34O13	30.1
38.94_615.2186m/z	HMDB0132811	M+H	C31H34O13	30.1
38.94_615.2186m/z	HMDB0132812	M+H	C31H34O13	30.1
38.94_615.2186m/z	HMDB0132813	M+H	C31H34O13	30.1
38.94_615.2186m/z	HMDB0132815	M+H	C31H34O13	30.1
38.94_615.2186m/z	HMDB0132816	M+H	C31H34O13	30.1
38.94_615.2186m/z	HMDB0132817	M+H	C31H34O13	30.1
38.94_615.2186m/z	HMDB0132819	M+H	C31H34O13	30.1
38.94_615.2186m/z	HMDB0132820	M+H	C31H34O13	30.1
38.94_615.2186m/z	HMDB0132821	M+H	C31H34O13	30.1
38.94_615.2186m/z	HMDB0132822	M+H	C31H34O13	30.1
38.94_615.2186m/z	HMDB0132823	M+H	C31H34O13	30.1
38.94_615.2186m/z	HMDB0132824	M+H	C31H34O13	30.1
38.94_615.2186m/z	HMDB0132825	M+H	C31H34O13	30.1
38.94_615.2186m/z	HMDB0132828	M+H	C31H34O13	30.1
38.94_615.2186m/z	HMDB0132831	M+H	C31H34O13	30.1
38.94_615.2186m/z	HMDB0132832	M+H	C31H34O13	30.1
38.94_615.2186m/z	HMDB0132833	M+H	C31H34O13	30.1
38.94_615.2186m/z	HMDB0132835	M+H	C31H34O13	30.1
38.94_615.2186m/z	HMDB0132836	M+H	C31H34O13	30.1
38.94_615.2186m/z	HMDB0132837	M+H	C31H34O13	30.1
38.94_615.2186m/z	HMDB0132838	M+H	C31H34O13	30.1
38.94_615.2186m/z	HMDB0132839	M+H	C31H34O13	30.1
38.94_615.2186m/z	HMDB0132840	M+H	C31H34O13	30.1
38.94_615.2186m/z	HMDB0132841	M+H	C31H34O13	30.1
38.94_615.2186m/z	HMDB0132842	M+H	C31H34O13	30.1
38.94_615.2186m/z	HMDB0132843	M+H	C31H34O13	30.1
38.94_615.2186m/z	HMDB0132844	M+H	C31H34O13	30.1
38.94_615.2186m/z	HMDB0132845	M+H	C31H34O13	30.1
38.94_615.2186m/z	HMDB0132846	M+H	C31H34O13	30.1
38.94_615.2186m/z	HMDB0132847	M+H	C31H34O13	30.1
38.94_615.2186m/z	HMDB0132848	M+H	C31H34O13	30.1
38.04_341.1982m/z	HMDB0014722	M+H-2H2O	C20H28N2O5	30.1
38.04_341.1982m/z	HMDB0015036	M+H-2H2O	C20H28N2O5	30.1
27.53_496.1463m/z	HMDB0014624	M+Na	C28H27NO4S	30.1
0.29_156.0199m/z	HMDB0002916	M+H	C6H5NO4	30.1
0.29_156.0199m/z	HMDB0006200	M+H	C6H5NO4	30.1
0.29_156.0199m/z	HMDB0062694	M+H	C6H5NO4	30.1
38.98_616.2241m/z	HMDB0000825	M+H-H2O	C23H39NO19	30.1
38.98_616.2241m/z	HMDB0006569	M+H-H2O	C23H39NO19	30.1
39.00_615.2230m/z	HMDB0129415	M+H	C31H34O13	30.1
39.00_615.2230m/z	HMDB0129416	M+H	C31H34O13	30.1
39.00_615.2230m/z	HMDB0129417	M+H	C31H34O13	30.1
39.00_615.2230m/z	HMDB0132767	M+H	C31H34O13	30.1
39.00_615.2230m/z	HMDB0132768	M+H	C31H34O13	30.1
39.00_615.2230m/z	HMDB0132770	M+H	C31H34O13	30.1
39.00_615.2230m/z	HMDB0132771	M+H	C31H34O13	30.1
39.00_615.2230m/z	HMDB0132772	M+H	C31H34O13	30.1
39.00_615.2230m/z	HMDB0132774	M+H	C31H34O13	30.1
39.00_615.2230m/z	HMDB0132775	M+H	C31H34O13	30.1

39.00_615.2230m/z	HMDB0132776	M+H	C31H34O13	30.1
39.00_615.2230m/z	HMDB0132777	M+H	C31H34O13	30.1
39.00_615.2230m/z	HMDB0132778	M+H	C31H34O13	30.1
39.00_615.2230m/z	HMDB0132779	M+H	C31H34O13	30.1
39.00_615.2230m/z	HMDB0132780	M+H	C31H34O13	30.1
39.00_615.2230m/z	HMDB0132782	M+H	C31H34O13	30.1
39.00_615.2230m/z	HMDB0132783	M+H	C31H34O13	30.1
39.00_615.2230m/z	HMDB0132784	M+H	C31H34O13	30.1
39.00_615.2230m/z	HMDB0132786	M+H	C31H34O13	30.1
39.00_615.2230m/z	HMDB0132787	M+H	C31H34O13	30.1
39.00_615.2230m/z	HMDB0132788	M+H	C31H34O13	30.1
39.00_615.2230m/z	HMDB0132789	M+H	C31H34O13	30.1
39.00_615.2230m/z	HMDB0132791	M+H	C31H34O13	30.1
39.00_615.2230m/z	HMDB0132792	M+H	C31H34O13	30.1
39.00_615.2230m/z	HMDB0132793	M+H	C31H34O13	30.1
39.00_615.2230m/z	HMDB0132811	M+H	C31H34O13	30.1
39.00_615.2230m/z	HMDB0132812	M+H	C31H34O13	30.1
39.00_615.2230m/z	HMDB0132813	M+H	C31H34O13	30.1
39.00_615.2230m/z	HMDB0132815	M+H	C31H34O13	30.1
39.00_615.2230m/z	HMDB0132816	M+H	C31H34O13	30.1
39.00_615.2230m/z	HMDB0132817	M+H	C31H34O13	30.1
39.00_615.2230m/z	HMDB0132819	M+H	C31H34O13	30.1
39.00_615.2230m/z	HMDB0132820	M+H	C31H34O13	30.1
39.00_615.2230m/z	HMDB0132821	M+H	C31H34O13	30.1
39.00_615.2230m/z	HMDB0132822	M+H	C31H34O13	30.1
39.00_615.2230m/z	HMDB0132823	M+H	C31H34O13	30.1
39.00_615.2230m/z	HMDB0132824	M+H	C31H34O13	30.1
39.00_615.2230m/z	HMDB0132825	M+H	C31H34O13	30.1
39.00_615.2230m/z	HMDB0132828	M+H	C31H34O13	30.1
39.00_615.2230m/z	HMDB0132831	M+H	C31H34O13	30.1
39.00_615.2230m/z	HMDB0132832	M+H	C31H34O13	30.1
39.00_615.2230m/z	HMDB0132833	M+H	C31H34O13	30.1
39.00_615.2230m/z	HMDB0132835	M+H	C31H34O13	30.1
39.00_615.2230m/z	HMDB0132836	M+H	C31H34O13	30.1
39.00_615.2230m/z	HMDB0132837	M+H	C31H34O13	30.1
39.00_615.2230m/z	HMDB0132838	M+H	C31H34O13	30.1
39.00_615.2230m/z	HMDB0132839	M+H	C31H34O13	30.1
39.00_615.2230m/z	HMDB0132840	M+H	C31H34O13	30.1
39.00_615.2230m/z	HMDB0132841	M+H	C31H34O13	30.1
39.00_615.2230m/z	HMDB0132842	M+H	C31H34O13	30.1
39.00_615.2230m/z	HMDB0132843	M+H	C31H34O13	30.1
39.00_615.2230m/z	HMDB0132844	M+H	C31H34O13	30.1
39.00_615.2230m/z	HMDB0132845	M+H	C31H34O13	30.1
39.00_615.2230m/z	HMDB0132846	M+H	C31H34O13	30.1
39.00_615.2230m/z	HMDB0132847	M+H	C31H34O13	30.1
39.00_615.2230m/z	HMDB0132848	M+H	C31H34O13	30.1
38.94_615.2186m/z	HMDB0129415	M+H	C31H34O13	30.1
38.94_615.2186m/z	HMDB0129416	M+H	C31H34O13	30.1
38.94_615.2186m/z	HMDB0129417	M+H	C31H34O13	30.1
38.94_615.2186m/z	HMDB0132767	M+H	C31H34O13	30.1

38.94_615.2186m/z	HMDB0132768	M+H	C31H34O13	30.1
38.94_615.2186m/z	HMDB0132770	M+H	C31H34O13	30.1
38.94_615.2186m/z	HMDB0132771	M+H	C31H34O13	30.1
38.94_615.2186m/z	HMDB0132772	M+H	C31H34O13	30.1
38.94_615.2186m/z	HMDB0132774	M+H	C31H34O13	30.1
38.94_615.2186m/z	HMDB0132775	M+H	C31H34O13	30.1
38.94_615.2186m/z	HMDB0132776	M+H	C31H34O13	30.1
38.94_615.2186m/z	HMDB0132777	M+H	C31H34O13	30.1
38.94_615.2186m/z	HMDB0132778	M+H	C31H34O13	30.1
38.94_615.2186m/z	HMDB0132779	M+H	C31H34O13	30.1
38.94_615.2186m/z	HMDB0132780	M+H	C31H34O13	30.1
38.94_615.2186m/z	HMDB0132782	M+H	C31H34O13	30.1
38.94_615.2186m/z	HMDB0132783	M+H	C31H34O13	30.1
38.94_615.2186m/z	HMDB0132784	M+H	C31H34O13	30.1
38.94_615.2186m/z	HMDB0132786	M+H	C31H34O13	30.1
38.94_615.2186m/z	HMDB0132787	M+H	C31H34O13	30.1
38.94_615.2186m/z	HMDB0132788	M+H	C31H34O13	30.1
38.94_615.2186m/z	HMDB0132789	M+H	C31H34O13	30.1
38.94_615.2186m/z	HMDB0132791	M+H	C31H34O13	30.1
38.94_615.2186m/z	HMDB0132792	M+H	C31H34O13	30.1
38.94_615.2186m/z	HMDB0132793	M+H	C31H34O13	30.1
38.94_615.2186m/z	HMDB0132811	M+H	C31H34O13	30.1
38.94_615.2186m/z	HMDB0132812	M+H	C31H34O13	30.1
38.94_615.2186m/z	HMDB0132813	M+H	C31H34O13	30.1
38.94_615.2186m/z	HMDB0132815	M+H	C31H34O13	30.1
38.94_615.2186m/z	HMDB0132816	M+H	C31H34O13	30.1
38.94_615.2186m/z	HMDB0132817	M+H	C31H34O13	30.1
38.94_615.2186m/z	HMDB0132819	M+H	C31H34O13	30.1
38.94_615.2186m/z	HMDB0132820	M+H	C31H34O13	30.1
38.94_615.2186m/z	HMDB0132821	M+H	C31H34O13	30.1
38.94_615.2186m/z	HMDB0132822	M+H	C31H34O13	30.1
38.94_615.2186m/z	HMDB0132823	M+H	C31H34O13	30.1
38.94_615.2186m/z	HMDB0132824	M+H	C31H34O13	30.1
38.94_615.2186m/z	HMDB0132825	M+H	C31H34O13	30.1
38.94_615.2186m/z	HMDB0132828	M+H	C31H34O13	30.1
38.94_615.2186m/z	HMDB0132831	M+H	C31H34O13	30.1
38.94_615.2186m/z	HMDB0132832	M+H	C31H34O13	30.1
38.94_615.2186m/z	HMDB0132833	M+H	C31H34O13	30.1
38.94_615.2186m/z	HMDB0132835	M+H	C31H34O13	30.1
38.94_615.2186m/z	HMDB0132836	M+H	C31H34O13	30.1
38.94_615.2186m/z	HMDB0132837	M+H	C31H34O13	30.1
38.94_615.2186m/z	HMDB0132838	M+H	C31H34O13	30.1
38.94_615.2186m/z	HMDB0132839	M+H	C31H34O13	30.1
38.94_615.2186m/z	HMDB0132840	M+H	C31H34O13	30.1
38.94_615.2186m/z	HMDB0132841	M+H	C31H34O13	30.1
38.94_615.2186m/z	HMDB0132842	M+H	C31H34O13	30.1
38.94_615.2186m/z	HMDB0132843	M+H	C31H34O13	30.1
38.94_615.2186m/z	HMDB0132844	M+H	C31H34O13	30.1
38.94_615.2186m/z	HMDB0132845	M+H	C31H34O13	30.1
38.94_615.2186m/z	HMDB0132846	M+H	C31H34O13	30.1

38.94_615.2186m/z	HMDB0132847	M+H	C31H34O13	30.1
38.94_615.2186m/z	HMDB0132848	M+H	C31H34O13	30.1
38.04_341.1982m/z	HMDB0014722	M+H-2H2O	C20H28N2O5	30.1
38.04_341.1982m/z	HMDB0015036	M+H-2H2O	C20H28N2O5	30.1
27.53_496.1463m/z	HMDB0014624	M+Na	C28H27NO4S	30.1

Mass Error (ppm)	Isotope Similarity	Theoretical Isotope Distribution
1.816.731.948	9.020.664.073	100 - 22.9 - 5.38 - 0.831 - 0.119
1.816.731.948	9.020.664.073	100 - 22.9 - 5.38 - 0.831 - 0.119
1.696.708.715	8.973.038.008	100 - 23 - 5.82 - 0.932 - 0.142
1.696.708.715	8.973.038.008	100 - 23 - 5.82 - 0.932 - 0.142
1.696.708.715	8.973.038.008	100 - 23 - 5.82 - 0.932 - 0.142
1.696.708.715	8.973.038.008	100 - 23 - 5.82 - 0.932 - 0.142
1.696.708.715	8.973.038.008	100 - 23 - 5.82 - 0.932 - 0.142
1.696.708.715	8.973.038.008	100 - 23 - 5.82 - 0.932 - 0.142
-1.565.071.067	8.077.684.174	100 - 19.1 - 4.18 - 0.566
-1.565.071.067	8.077.684.174	100 - 19.1 - 4.18 - 0.566
-1.565.071.067	8.077.684.174	100 - 19.1 - 4.18 - 0.566
-1.565.071.067	8.077.684.174	100 - 19.1 - 4.18 - 0.566
-1.565.071.067	8.077.684.174	100 - 19.1 - 4.18 - 0.566
-1.565.071.067	8.077.684.174	100 - 19.1 - 4.18 - 0.566
-1.565.071.067	8.077.684.174	100 - 19.1 - 4.18 - 0.566
-1.565.071.067	8.077.684.174	100 - 19.1 - 4.18 - 0.566
-4.452.615.482	8.308.858.912	100 - 16.2 - 3.7 - 0.457
-4.452.615.482	8.308.858.912	100 - 16.2 - 3.7 - 0.457
-5.228.828.358	8.327.628.913	100 - 44.1 - 11.5 - 2.23 - 0.349
-5.228.828.358	8.327.628.913	100 - 44.1 - 11.5 - 2.23 - 0.349
-5.228.828.358	8.327.628.913	100 - 44.1 - 11.5 - 2.23 - 0.349
-1.051.492.245	7.869.278.511	100 - 23.5 - 3.25 - 0.333
-1.051.492.245	7.869.278.511	100 - 23.5 - 3.25 - 0.333
-5.228.828.358	8.327.628.913	100 - 44.1 - 11.5 - 2.23 - 0.349
-5.228.828.358	8.327.628.913	100 - 44.1 - 11.5 - 2.23 - 0.349
-5.228.828.358	8.327.628.913	100 - 44.1 - 11.5 - 2.23 - 0.349
-1.051.492.245	7.869.278.511	100 - 23.5 - 3.25 - 0.333
-1.051.492.245	7.869.278.511	100 - 23.5 - 3.25 - 0.333
-2.084.679.526	7.937.090.878	100 - 26.8 - 7.14 - 1.27 - 0.208
-2.084.679.527	7.937.090.878	100 - 26.8 - 7.14 - 1.27 - 0.208
-2.084.679.526	7.937.090.878	100 - 26.8 - 7.14 - 1.27 - 0.208
-2.084.679.527	7.937.090.878	100 - 26.8 - 7.14 - 1.27 - 0.208
2.895.326.239	7.973.545.924	100 - 22.4 - 2.8 - 0.254
2.895.326.239	7.973.545.924	100 - 22.4 - 2.8 - 0.254
64.359.989	8.038.185.957	100 - 21.4 - 2.78 - 0.271
64.359.989	8.038.185.957	100 - 21.4 - 2.78 - 0.271
2.378.515.589	9.386.541.213	100 - 5.98 - 0.56
2.378.515.589	9.386.541.213	100 - 5.98 - 0.56
2.378.515.589	9.386.541.213	100 - 5.98 - 0.56
2.378.515.589	9.386.541.213	100 - 5.98 - 0.56
2.378.515.589	9.386.541.213	100 - 5.98 - 0.56
2.378.515.589	9.386.541.213	100 - 5.98 - 0.56
2.378.515.589	9.386.541.213	100 - 5.98 - 0.56
2.378.515.589	9.386.541.213	100 - 5.98 - 0.56
2.378.515.589	9.386.541.213	100 - 5.98 - 0.56
2.378.515.589	9.386.541.213	100 - 5.98 - 0.56
-5.564.849.437	7.728.332.677	100 - 24.7 - 4.16 - 0.526
1.289.553.642	8.450.122.254	100 - 30.3 - 6.48 - 1.04 - 0.138

1.246.953.867	8.427.802.309	100 - 30.3 - 6.71 - 1.1 - 0.152
1.246.953.867	8.427.802.309	100 - 30.3 - 6.71 - 1.1 - 0.152
1.806.025.151	728.976.974	100 - 30.3 - 5.88 - 0.854 - 0.101
1.806.025.151	728.976.974	100 - 30.3 - 5.88 - 0.854 - 0.101
1.806.025.151	728.976.974	100 - 30.3 - 5.88 - 0.854 - 0.101
1.806.025.151	728.976.974	100 - 30.3 - 5.88 - 0.854 - 0.101
1.726.506.549	8.630.633.261	100 - 39.9 - 10.4 - 2.04 - 0.329
1.726.506.549	8.630.633.261	100 - 39.9 - 10.4 - 2.04 - 0.329
1.726.506.549	8.630.633.261	100 - 39.9 - 10.4 - 2.04 - 0.329
8.022.239.675	7.786.048.389	100 - 22.8 - 4.94 - 0.731
1.726.506.549	8.630.633.261	100 - 39.9 - 10.4 - 2.04 - 0.329
1.726.506.549	8.630.633.261	100 - 39.9 - 10.4 - 2.04 - 0.329
1.726.506.549	8.630.633.261	100 - 39.9 - 10.4 - 2.04 - 0.329
8.022.239.675	7.786.048.389	100 - 22.8 - 4.94 - 0.731
-1.480.719.457	8.332.409.642	100 - 27.5 - 10 - 2.02 - 0.361
-1.480.719.457	8.332.409.642	100 - 27.5 - 10 - 2.02 - 0.361
-1.480.719.457	8.332.409.642	100 - 27.5 - 10 - 2.02 - 0.361
-1.480.719.457	8.332.409.642	100 - 27.5 - 10 - 2.02 - 0.361
-4.705.949.144	728.212.774	100 - 26.2 - 9.06 - 1.74 - 0.28
1.485.857.441	8.270.237.267	100 - 17.9 - 2.74 - 0.299
1.485.857.441	8.270.237.267	100 - 17.9 - 2.74 - 0.299
2.384.745.986	6.952.247.037	100 - 33.5 - 8.52 - 1.6 - 0.255
-0.118742392	6.445.408.393	100 - 42.5 - 10.5 - 1.89 - 0.273
-9.130.519.889	7.386.928.233	100 - 26.8 - 7.14 - 1.27 - 0.208
-9.130.519.889	7.386.928.233	100 - 26.8 - 7.14 - 1.27 - 0.208
-3.453.916.598	6.710.130.002	100 - 35.8 - 10.5 - 2.24 - 0.414
-3.453.916.598	6.710.130.002	100 - 35.8 - 10.5 - 2.24 - 0.414
-3.453.916.598	6.710.130.002	100 - 35.8 - 10.5 - 2.24 - 0.414
-3.453.916.598	6.710.130.002	100 - 35.8 - 10.5 - 2.24 - 0.414
-3.453.916.598	6.710.130.002	100 - 35.8 - 10.5 - 2.24 - 0.414
-3.453.916.598	6.710.130.002	100 - 35.8 - 10.5 - 2.24 - 0.414
-3.453.916.598	6.710.130.002	100 - 35.8 - 10.5 - 2.24 - 0.414
-3.453.916.598	6.710.130.002	100 - 35.8 - 10.5 - 2.24 - 0.414
-3.453.916.598	6.710.130.002	100 - 35.8 - 10.5 - 2.24 - 0.414
-3.453.916.598	6.710.130.002	100 - 35.8 - 10.5 - 2.24 - 0.414
-3.453.916.598	6.710.130.002	100 - 35.8 - 10.5 - 2.24 - 0.414
-3.453.916.598	6.710.130.002	100 - 35.8 - 10.5 - 2.24 - 0.414
-3.453.916.598	6.710.130.002	100 - 35.8 - 10.5 - 2.24 - 0.414
141.926.193	7.747.896.643	100 - 19.4 - 8.15 - 1.3 - 0.231
276.353.559	6.579.736.483	100 - 39.8 - 9.99 - 1.87 - 0.287
276.353.559	6.579.736.483	100 - 39.8 - 9.99 - 1.87 - 0.287
276.353.559	6.579.736.483	100 - 39.8 - 9.99 - 1.87 - 0.287
141.926.193	7.747.896.643	100 - 19.4 - 8.15 - 1.3 - 0.231
276.353.559	6.579.736.483	100 - 39.8 - 9.99 - 1.87 - 0.287
276.353.559	6.579.736.483	100 - 39.8 - 9.99 - 1.87 - 0.287
276.353.559	6.579.736.483	100 - 39.8 - 9.99 - 1.87 - 0.287
-1.491.648.852	7.733.729.493	100 - 23 - 5.39 - 0.835 - 0.119
-1.905.963.047	6.445.408.393	100 - 42.5 - 10.5 - 1.89 - 0.273
6.388.584.759	6.822.276.909	100 - 35.6 - 9.02 - 1.71 - 0.27
3.167.556.779	6.444.456.546	100 - 40.1 - 11.9 - 2.64 - 0.493

3.167.556.779	6.444.456.546	100 - 40.1 - 11.9 - 2.64 - 0.493
3.167.556.779	6.444.456.546	100 - 40.1 - 11.9 - 2.64 - 0.493
3.167.556.779	6.444.456.546	100 - 40.1 - 11.9 - 2.64 - 0.493
3.167.556.779	6.444.456.546	100 - 40.1 - 11.9 - 2.64 - 0.493
3.167.556.779	6.444.456.546	100 - 40.1 - 11.9 - 2.64 - 0.493
3.167.556.779	6.444.456.546	100 - 40.1 - 11.9 - 2.64 - 0.493
3.167.556.779	6.444.456.546	100 - 40.1 - 11.9 - 2.64 - 0.493
3.167.556.779	6.444.456.546	100 - 40.1 - 11.9 - 2.64 - 0.493
3.167.556.779	6.444.456.546	100 - 40.1 - 11.9 - 2.64 - 0.493
3.167.556.779	6.444.456.546	100 - 40.1 - 11.9 - 2.64 - 0.493
3.167.556.779	6.444.456.546	100 - 40.1 - 11.9 - 2.64 - 0.493
8.175.820.239	6.822.276.909	100 - 35.6 - 9.02 - 1.71 - 0.27
1.874.704.691	7.792.469.931	100 - 24.3 - 3.64 - 0.408
1.874.704.691	7.792.469.931	100 - 24.3 - 3.64 - 0.408
4.186.559.156	9.310.047.107	100 - 7 - 0.414
8.175.820.239	6.822.276.909	100 - 35.6 - 9.02 - 1.71 - 0.27
-6.301.990.458	6.552.008.835	100 - 38 - 11.6 - 2.56 - 0.488
-1.854.540.535	771.190.327	100 - 24.7 - 4.37 - 0.578
-1.854.540.535	771.190.327	100 - 24.7 - 4.37 - 0.578
-1.854.540.535	771.190.327	100 - 24.7 - 4.37 - 0.578
-1.854.540.535	771.190.327	100 - 24.7 - 4.37 - 0.578
4.313.137.491	630.254.915	100 - 43.2 - 12.4 - 2.66 - 0.473
1.490.140.493	727.205.638	100 - 25.4 - 9.87 - 1.92 - 0.36
-9.839.207.154	6.770.611.539	100 - 33 - 11.6 - 2.61 - 0.484
-2.619.347.796	8.143.883.674	100 - 19.6 - 2.85 - 0.308
1.367.457.802	7.115.846.309	100 - 32.4 - 6.92 - 1.11 - 0.146
1.367.457.802	7.115.846.309	100 - 32.4 - 6.92 - 1.11 - 0.146
1.415.452.391	7.134.694.529	100 - 32.3 - 6.69 - 1.04 - 0.132
1.415.452.391	7.134.694.529	100 - 32.3 - 6.69 - 1.04 - 0.132
-1.061.683.198	6.771.559.477	100 - 37.8 - 8.37 - 1.37 - 0.18
-5.138.438.099	9.447.897.499	100 - 4.92 - 0.919
-5.138.438.099	9.447.897.499	100 - 4.92 - 0.919
-5.138.438.099	9.447.897.499	100 - 4.92 - 0.919
-5.138.438.099	9.447.897.499	100 - 4.92 - 0.919
-5.138.438.099	9.447.897.499	100 - 4.92 - 0.919
-1.061.683.198	6.771.559.477	100 - 37.8 - 8.37 - 1.37 - 0.18
-186.704.287	7.258.062.165	100 - 31.4 - 5.6 - 0.727
4.421.005.008	8.900.129.962	100 - 11.4 - 0.997
4.421.005.008	8.900.129.962	100 - 11.4 - 0.997
4.421.005.008	8.900.129.962	100 - 11.4 - 0.997
4.421.005.008	8.900.129.962	100 - 11.4 - 0.997
-186.704.287	7.258.062.165	100 - 31.4 - 5.6 - 0.727
4.421.005.008	8.900.129.962	100 - 11.4 - 0.997
4.421.005.008	8.900.129.962	100 - 11.4 - 0.997
4.421.005.008	8.900.129.962	100 - 11.4 - 0.997
4.421.005.008	8.900.129.962	100 - 11.4 - 0.997
2.054.858.767	7.369.698.317	100 - 28.2 - 6.3 - 1.03 - 0.143
2.054.858.767	7.369.698.317	100 - 28.2 - 6.3 - 1.03 - 0.143
8.856.224.752	632.120.445	100 - 44.1 - 11.5 - 2.23 - 0.349
8.856.224.752	632.120.445	100 - 44.1 - 11.5 - 2.23 - 0.349

8.856.224.752	632.120.445	100 - 44.1 - 11.5 - 2.23 - 0.349
2.181.267.727	7.467.324.053	100 - 23.1 - 8.92 - 1.61 - 0.283
2.054.858.767	7.369.698.317	100 - 28.2 - 6.3 - 1.03 - 0.143
2.054.858.767	7.369.698.317	100 - 28.2 - 6.3 - 1.03 - 0.143
8.856.224.752	632.120.445	100 - 44.1 - 11.5 - 2.23 - 0.349
8.856.224.752	632.120.445	100 - 44.1 - 11.5 - 2.23 - 0.349
8.856.224.752	632.120.445	100 - 44.1 - 11.5 - 2.23 - 0.349
2.181.267.727	7.467.324.053	100 - 23.1 - 8.92 - 1.61 - 0.283
1.697.426.934	6.879.365.173	100 - 34.5 - 8.87 - 1.69 - 0.271
1.697.426.934	6.879.365.173	100 - 34.5 - 8.87 - 1.69 - 0.271
-210.078.409	7.209.685.301	100 - 30.4 - 6.94 - 1.17 - 0.167
-210.078.409	7.209.685.301	100 - 30.4 - 6.94 - 1.17 - 0.167
-210.078.409	7.209.685.301	100 - 30.4 - 6.94 - 1.17 - 0.167
-4.050.339.727	8.470.306.022	100 - 43.2 - 10.3 - 1.78 - 0.243
4.927.994.919	8.921.848.048	100 - 11.3 - 0.785
1.697.426.934	6.879.365.173	100 - 34.5 - 8.87 - 1.69 - 0.271
1.697.426.934	6.879.365.173	100 - 34.5 - 8.87 - 1.69 - 0.271
-210.078.409	7.209.685.301	100 - 30.4 - 6.94 - 1.17 - 0.167
-210.078.409	7.209.685.301	100 - 30.4 - 6.94 - 1.17 - 0.167
-210.078.409	7.209.685.301	100 - 30.4 - 6.94 - 1.17 - 0.167
-4.050.339.727	8.470.306.022	100 - 43.2 - 10.3 - 1.78 - 0.243
4.927.994.919	8.921.848.048	100 - 11.3 - 0.785
1.796.975.584	6.915.731.428	100 - 34.4 - 8.41 - 1.54 - 0.234
1.796.975.584	6.915.731.428	100 - 34.4 - 8.41 - 1.54 - 0.234
1.796.975.584	6.915.731.428	100 - 34.4 - 8.41 - 1.54 - 0.234
1.796.975.584	6.915.731.428	100 - 34.4 - 8.41 - 1.54 - 0.234
1.796.975.584	6.915.731.428	100 - 34.4 - 8.41 - 1.54 - 0.234
1.796.975.584	6.915.731.428	100 - 34.4 - 8.41 - 1.54 - 0.234
1.796.975.584	6.915.731.428	100 - 34.4 - 8.41 - 1.54 - 0.234
1.796.975.584	6.915.731.428	100 - 34.4 - 8.41 - 1.54 - 0.234
1.796.975.584	6.915.731.428	100 - 34.4 - 8.41 - 1.54 - 0.234
1.796.975.584	6.915.731.428	100 - 34.4 - 8.41 - 1.54 - 0.234
1.796.975.584	6.915.731.428	100 - 34.4 - 8.41 - 1.54 - 0.234
1.796.975.584	6.915.731.428	100 - 34.4 - 8.41 - 1.54 - 0.234
1.796.975.584	6.915.731.428	100 - 34.4 - 8.41 - 1.54 - 0.234
1.796.975.584	6.915.731.428	100 - 34.4 - 8.41 - 1.54 - 0.234
1.796.975.584	6.915.731.428	100 - 34.4 - 8.41 - 1.54 - 0.234
1.796.975.584	6.915.731.428	100 - 34.4 - 8.41 - 1.54 - 0.234
1.796.975.584	6.915.731.428	100 - 34.4 - 8.41 - 1.54 - 0.234
1.796.975.584	6.915.731.428	100 - 34.4 - 8.41 - 1.54 - 0.234
1.796.975.584	6.915.731.428	100 - 34.4 - 8.41 - 1.54 - 0.234
1.796.975.584	6.915.731.428	100 - 34.4 - 8.41 - 1.54 - 0.234
1.796.975.584	6.915.731.428	100 - 34.4 - 8.41 - 1.54 - 0.234
1.796.975.584	6.915.731.428	100 - 34.4 - 8.41 - 1.54 - 0.234
1.796.975.584	6.915.731.428	100 - 34.4 - 8.41 - 1.54 - 0.234
1.796.975.584	6.915.731.428	100 - 34.4 - 8.41 - 1.54 - 0.234
1.796.975.584	6.915.731.428	100 - 34.4 - 8.41 - 1.54 - 0.234
1.796.975.584	6.915.731.428	100 - 34.4 - 8.41 - 1.54 - 0.234
1.796.975.584	6.915.731.428	100 - 34.4 - 8.41 - 1.54 - 0.234
1.796.975.584	6.915.731.428	100 - 34.4 - 8.41 - 1.54 - 0.234

1.796.975.584	6.915.731.428	100 - 34.4 - 8.41 - 1.54 - 0.234
1.796.975.584	6.915.731.428	100 - 34.4 - 8.41 - 1.54 - 0.234
1.796.975.584	6.915.731.428	100 - 34.4 - 8.41 - 1.54 - 0.234
1.796.975.584	6.915.731.428	100 - 34.4 - 8.41 - 1.54 - 0.234
1.796.975.584	6.915.731.428	100 - 34.4 - 8.41 - 1.54 - 0.234
1.796.975.584	6.915.731.428	100 - 34.4 - 8.41 - 1.54 - 0.234
1.796.975.584	6.915.731.428	100 - 34.4 - 8.41 - 1.54 - 0.234
1.796.975.584	6.915.731.428	100 - 34.4 - 8.41 - 1.54 - 0.234
1.796.975.584	6.915.731.428	100 - 34.4 - 8.41 - 1.54 - 0.234
1.796.975.584	6.915.731.428	100 - 34.4 - 8.41 - 1.54 - 0.234
1.796.975.584	6.915.731.428	100 - 34.4 - 8.41 - 1.54 - 0.234
1.796.975.584	6.915.731.428	100 - 34.4 - 8.41 - 1.54 - 0.234
1.796.975.584	6.915.731.428	100 - 34.4 - 8.41 - 1.54 - 0.234
1.796.975.584	6.915.731.428	100 - 34.4 - 8.41 - 1.54 - 0.234
1.796.975.584	6.915.731.428	100 - 34.4 - 8.41 - 1.54 - 0.234
1.796.975.584	6.915.731.428	100 - 34.4 - 8.41 - 1.54 - 0.234
1.796.975.584	6.915.731.428	100 - 34.4 - 8.41 - 1.54 - 0.234
1.796.975.584	6.915.731.428	100 - 34.4 - 8.41 - 1.54 - 0.234
1.796.975.584	6.915.731.428	100 - 34.4 - 8.41 - 1.54 - 0.234
1.796.975.584	6.915.731.428	100 - 34.4 - 8.41 - 1.54 - 0.234
1.796.975.584	6.915.731.428	100 - 34.4 - 8.41 - 1.54 - 0.234
1.796.975.584	6.915.731.428	100 - 34.4 - 8.41 - 1.54 - 0.234
1.796.975.584	6.915.731.428	100 - 34.4 - 8.41 - 1.54 - 0.234
1.796.975.584	6.915.731.428	100 - 34.4 - 8.41 - 1.54 - 0.234
1.796.975.584	6.915.731.428	100 - 34.4 - 8.41 - 1.54 - 0.234
1.796.975.584	6.915.731.428	100 - 34.4 - 8.41 - 1.54 - 0.234
1.796.975.584	6.915.731.428	100 - 34.4 - 8.41 - 1.54 - 0.234
1.796.975.584	6.915.731.428	100 - 34.4 - 8.41 - 1.54 - 0.234
-1.468.778.121	6.640.097.707	100 - 39.9 - 9 - 1.47 - 0.192
2.428.194.221	7.391.725.605	100 - 34.5 - 8.87 - 1.69 - 0.271
2.428.194.221	7.391.725.605	100 - 34.5 - 8.87 - 1.69 - 0.271
174.310.802	6.879.365.173	100 - 34.5 - 8.87 - 1.69 - 0.271
174.310.802	6.879.365.173	100 - 34.5 - 8.87 - 1.69 - 0.271
-3.308.433.371	7.979.516.181	100 - 22.1 - 2.94 - 0.291
-3.308.433.371	7.979.516.181	100 - 22.1 - 2.94 - 0.291
-3.308.433.371	7.979.516.181	100 - 22.1 - 2.94 - 0.291
-3.308.433.371	7.979.516.181	100 - 22.1 - 2.94 - 0.291
-3.308.433.371	7.979.516.181	100 - 22.1 - 2.94 - 0.291
-3.308.433.371	7.979.516.181	100 - 22.1 - 2.94 - 0.291
-3.308.433.371	7.979.516.181	100 - 22.1 - 2.94 - 0.291
-3.308.433.371	7.979.516.181	100 - 22.1 - 2.94 - 0.291
-3.308.433.371	7.979.516.181	100 - 22.1 - 2.94 - 0.291
-3.308.433.371	7.979.516.181	100 - 22.1 - 2.94 - 0.291
-3.308.433.371	7.979.516.181	100 - 22.1 - 2.94 - 0.291
-3.308.433.371	7.979.516.181	100 - 22.1 - 2.94 - 0.291
-3.308.433.371	7.979.516.181	100 - 22.1 - 2.94 - 0.291
-3.308.433.371	7.979.516.181	100 - 22.1 - 2.94 - 0.291
-3.308.433.371	7.979.516.181	100 - 22.1 - 2.94 - 0.291
-3.308.433.371	7.979.516.181	100 - 22.1 - 2.94 - 0.291
-3.308.433.371	7.979.516.181	100 - 22.1 - 2.94 - 0.291

-3.308.433.371	7.979.516.181	100 - 22.1 - 2.94 - 0.291
-3.308.433.371	7.979.516.181	100 - 22.1 - 2.94 - 0.291
-3.308.433.371	7.979.516.181	100 - 22.1 - 2.94 - 0.291
-3.308.433.371	7.979.516.181	100 - 22.1 - 2.94 - 0.291
-3.308.433.371	7.979.516.181	100 - 22.1 - 2.94 - 0.291
-3.308.433.371	7.979.516.181	100 - 22.1 - 2.94 - 0.291
-3.308.433.371	7.979.516.181	100 - 22.1 - 2.94 - 0.291
-3.308.433.371	7.979.516.181	100 - 22.1 - 2.94 - 0.291
-3.308.433.371	7.979.516.181	100 - 22.1 - 2.94 - 0.291
-3.308.433.371	7.979.516.181	100 - 22.1 - 2.94 - 0.291
-3.308.433.371	7.979.516.181	100 - 22.1 - 2.94 - 0.291
-3.308.433.371	7.979.516.181	100 - 22.1 - 2.94 - 0.291
-3.308.433.371	7.979.516.181	100 - 22.1 - 2.94 - 0.291
-3.308.433.371	7.979.516.181	100 - 22.1 - 2.94 - 0.291
-3.308.433.371	7.979.516.181	100 - 22.1 - 2.94 - 0.291
-3.308.433.371	7.979.516.181	100 - 22.1 - 2.94 - 0.291
-3.308.433.371	7.979.516.181	100 - 22.1 - 2.94 - 0.291
-3.308.433.371	7.979.516.181	100 - 22.1 - 2.94 - 0.291
-3.308.433.371	7.979.516.181	100 - 22.1 - 2.94 - 0.291
-3.308.433.371	7.979.516.181	100 - 22.1 - 2.94 - 0.291
1.796.975.584	6.915.731.428	100 - 34.4 - 8.41 - 1.54 - 0.234
1.796.975.584	6.915.731.428	100 - 34.4 - 8.41 - 1.54 - 0.234
1.796.975.584	6.915.731.428	100 - 34.4 - 8.41 - 1.54 - 0.234
1.796.975.584	6.915.731.428	100 - 34.4 - 8.41 - 1.54 - 0.234
1.796.975.584	6.915.731.428	100 - 34.4 - 8.41 - 1.54 - 0.234
1.796.975.584	6.915.731.428	100 - 34.4 - 8.41 - 1.54 - 0.234
1.796.975.584	6.915.731.428	100 - 34.4 - 8.41 - 1.54 - 0.234
1.796.975.584	6.915.731.428	100 - 34.4 - 8.41 - 1.54 - 0.234
1.796.975.584	6.915.731.428	100 - 34.4 - 8.41 - 1.54 - 0.234
1.796.975.584	6.915.731.428	100 - 34.4 - 8.41 - 1.54 - 0.234
1.796.975.584	6.915.731.428	100 - 34.4 - 8.41 - 1.54 - 0.234
1.796.975.584	6.915.731.428	100 - 34.4 - 8.41 - 1.54 - 0.234
1.796.975.584	6.915.731.428	100 - 34.4 - 8.41 - 1.54 - 0.234
1.796.975.584	6.915.731.428	100 - 34.4 - 8.41 - 1.54 - 0.234
1.796.975.584	6.915.731.428	100 - 34.4 - 8.41 - 1.54 - 0.234
1.796.975.584	6.915.731.428	100 - 34.4 - 8.41 - 1.54 - 0.234
1.796.975.584	6.915.731.428	100 - 34.4 - 8.41 - 1.54 - 0.234
1.796.975.584	6.915.731.428	100 - 34.4 - 8.41 - 1.54 - 0.234
1.796.975.584	6.915.731.428	100 - 34.4 - 8.41 - 1.54 - 0.234
1.796.975.584	6.915.731.428	100 - 34.4 - 8.41 - 1.54 - 0.234
1.796.975.584	6.915.731.428	100 - 34.4 - 8.41 - 1.54 - 0.234
1.796.975.584	6.915.731.428	100 - 34.4 - 8.41 - 1.54 - 0.234
1.796.975.584	6.915.731.428	100 - 34.4 - 8.41 - 1.54 - 0.234
1.796.975.584	6.915.731.428	100 - 34.4 - 8.41 - 1.54 - 0.234
1.796.975.584	6.915.731.428	100 - 34.4 - 8.41 - 1.54 - 0.234
1.796.975.584	6.915.731.428	100 - 34.4 - 8.41 - 1.54 - 0.234
1.796.975.584	6.915.731.428	100 - 34.4 - 8.41 - 1.54 - 0.234
1.796.975.584	6.915.731.428	100 - 34.4 - 8.41 - 1.54 - 0.234
1.796.975.584	6.915.731.428	100 - 34.4 - 8.41 - 1.54 - 0.234
1.796.975.584	6.915.731.428	100 - 34.4 - 8.41 - 1.54 - 0.234

1.796.975.584	6.915.731.428	100 - 34.4 - 8.41 - 1.54 - 0.234
1.796.975.584	6.915.731.428	100 - 34.4 - 8.41 - 1.54 - 0.234
1.796.975.584	6.915.731.428	100 - 34.4 - 8.41 - 1.54 - 0.234
1.796.975.584	6.915.731.428	100 - 34.4 - 8.41 - 1.54 - 0.234
1.796.975.584	6.915.731.428	100 - 34.4 - 8.41 - 1.54 - 0.234
1.796.975.584	6.915.731.428	100 - 34.4 - 8.41 - 1.54 - 0.234
1.796.975.584	6.915.731.428	100 - 34.4 - 8.41 - 1.54 - 0.234
1.796.975.584	6.915.731.428	100 - 34.4 - 8.41 - 1.54 - 0.234
1.796.975.584	6.915.731.428	100 - 34.4 - 8.41 - 1.54 - 0.234
1.796.975.584	6.915.731.428	100 - 34.4 - 8.41 - 1.54 - 0.234
1.796.975.584	6.915.731.428	100 - 34.4 - 8.41 - 1.54 - 0.234
1.796.975.584	6.915.731.428	100 - 34.4 - 8.41 - 1.54 - 0.234
1.796.975.584	6.915.731.428	100 - 34.4 - 8.41 - 1.54 - 0.234
1.796.975.584	6.915.731.428	100 - 34.4 - 8.41 - 1.54 - 0.234
1.796.975.584	6.915.731.428	100 - 34.4 - 8.41 - 1.54 - 0.234
1.796.975.584	6.915.731.428	100 - 34.4 - 8.41 - 1.54 - 0.234
1.796.975.584	6.915.731.428	100 - 34.4 - 8.41 - 1.54 - 0.234
1.796.975.584	6.915.731.428	100 - 34.4 - 8.41 - 1.54 - 0.234
1.796.975.584	6.915.731.428	100 - 34.4 - 8.41 - 1.54 - 0.234
1.796.975.584	6.915.731.428	100 - 34.4 - 8.41 - 1.54 - 0.234
1.796.975.584	6.915.731.428	100 - 34.4 - 8.41 - 1.54 - 0.234
1.796.975.584	6.915.731.428	100 - 34.4 - 8.41 - 1.54 - 0.234
1.796.975.584	6.915.731.428	100 - 34.4 - 8.41 - 1.54 - 0.234
1.796.975.584	6.915.731.428	100 - 34.4 - 8.41 - 1.54 - 0.234
1.796.975.584	6.915.731.428	100 - 34.4 - 8.41 - 1.54 - 0.234
1.796.975.584	6.915.731.428	100 - 34.4 - 8.41 - 1.54 - 0.234
-1.468.778.121	6.640.097.707	100 - 39.9 - 9 - 1.47 - 0.192
2.428.194.221	7.391.725.605	100 - 34.5 - 8.87 - 1.69 - 0.271
2.428.194.221	7.391.725.605	100 - 34.5 - 8.87 - 1.69 - 0.271
174.310.802	6.879.365.173	100 - 34.5 - 8.87 - 1.69 - 0.271
174.310.802	6.879.365.173	100 - 34.5 - 8.87 - 1.69 - 0.271
-3.308.433.371	7.979.516.181	100 - 22.1 - 2.94 - 0.291
-3.308.433.371	7.979.516.181	100 - 22.1 - 2.94 - 0.291
-3.308.433.371	7.979.516.181	100 - 22.1 - 2.94 - 0.291
-3.308.433.371	7.979.516.181	100 - 22.1 - 2.94 - 0.291
-3.308.433.371	7.979.516.181	100 - 22.1 - 2.94 - 0.291
-3.308.433.371	7.979.516.181	100 - 22.1 - 2.94 - 0.291
-3.308.433.371	7.979.516.181	100 - 22.1 - 2.94 - 0.291
-3.308.433.371	7.979.516.181	100 - 22.1 - 2.94 - 0.291
-3.308.433.371	7.979.516.181	100 - 22.1 - 2.94 - 0.291
-3.308.433.371	7.979.516.181	100 - 22.1 - 2.94 - 0.291
-3.308.433.371	7.979.516.181	100 - 22.1 - 2.94 - 0.291
-3.308.433.371	7.979.516.181	100 - 22.1 - 2.94 - 0.291
-3.308.433.371	7.979.516.181	100 - 22.1 - 2.94 - 0.291
-3.308.433.371	7.979.516.181	100 - 22.1 - 2.94 - 0.291
-3.308.433.371	7.979.516.181	100 - 22.1 - 2.94 - 0.291
-3.308.433.371	7.979.516.181	100 - 22.1 - 2.94 - 0.291
-3.308.433.371	7.979.516.181	100 - 22.1 - 2.94 - 0.291
-3.308.433.371	7.979.516.181	100 - 22.1 - 2.94 - 0.291
-3.308.433.371	7.979.516.181	100 - 22.1 - 2.94 - 0.291

-3.308.433.371	7.979.516.181	100 - 22.1 - 2.94 - 0.291
-3.308.433.371	7.979.516.181	100 - 22.1 - 2.94 - 0.291
-3.308.433.371	7.979.516.181	100 - 22.1 - 2.94 - 0.291
-3.308.433.371	7.979.516.181	100 - 22.1 - 2.94 - 0.291
-3.308.433.371	7.979.516.181	100 - 22.1 - 2.94 - 0.291
-3.308.433.371	7.979.516.181	100 - 22.1 - 2.94 - 0.291
-3.308.433.371	7.979.516.181	100 - 22.1 - 2.94 - 0.291
-3.308.433.371	7.979.516.181	100 - 22.1 - 2.94 - 0.291
-3.308.433.371	7.979.516.181	100 - 22.1 - 2.94 - 0.291
-3.308.433.371	7.979.516.181	100 - 22.1 - 2.94 - 0.291
-3.308.433.371	7.979.516.181	100 - 22.1 - 2.94 - 0.291
-3.308.433.371	7.979.516.181	100 - 22.1 - 2.94 - 0.291
-3.308.433.371	7.979.516.181	100 - 22.1 - 2.94 - 0.291
-3.308.433.371	7.979.516.181	100 - 22.1 - 2.94 - 0.291
-3.308.433.371	7.979.516.181	100 - 22.1 - 2.94 - 0.291
-3.308.433.371	7.979.516.181	100 - 22.1 - 2.94 - 0.291
-3.308.433.371	7.979.516.181	100 - 22.1 - 2.94 - 0.291
-3.308.433.371	7.979.516.181	100 - 22.1 - 2.94 - 0.291
2.486.487.164	7.396.874.886	100 - 26.4 - 7.26 - 1.3 - 0.219
2.486.487.164	7.396.874.886	100 - 26.4 - 7.26 - 1.3 - 0.219
2.570.600.354	7.430.800.341	100 - 34.4 - 8.41 - 1.54 - 0.234
2.570.600.354	7.430.800.341	100 - 34.4 - 8.41 - 1.54 - 0.234
2.570.600.354	7.430.800.341	100 - 34.4 - 8.41 - 1.54 - 0.234
2.570.600.354	7.430.800.341	100 - 34.4 - 8.41 - 1.54 - 0.234
2.570.600.354	7.430.800.341	100 - 34.4 - 8.41 - 1.54 - 0.234
2.570.600.354	7.430.800.341	100 - 34.4 - 8.41 - 1.54 - 0.234
2.570.600.354	7.430.800.341	100 - 34.4 - 8.41 - 1.54 - 0.234
2.570.600.354	7.430.800.341	100 - 34.4 - 8.41 - 1.54 - 0.234
2.570.600.354	7.430.800.341	100 - 34.4 - 8.41 - 1.54 - 0.234
2.570.600.354	7.430.800.341	100 - 34.4 - 8.41 - 1.54 - 0.234
2.570.600.354	7.430.800.341	100 - 34.4 - 8.41 - 1.54 - 0.234
2.570.600.354	7.430.800.341	100 - 34.4 - 8.41 - 1.54 - 0.234
2.570.600.354	7.430.800.341	100 - 34.4 - 8.41 - 1.54 - 0.234
2.570.600.354	7.430.800.341	100 - 34.4 - 8.41 - 1.54 - 0.234
2.570.600.354	7.430.800.341	100 - 34.4 - 8.41 - 1.54 - 0.234
2.570.600.354	7.430.800.341	100 - 34.4 - 8.41 - 1.54 - 0.234
2.570.600.354	7.430.800.341	100 - 34.4 - 8.41 - 1.54 - 0.234
2.570.600.354	7.430.800.341	100 - 34.4 - 8.41 - 1.54 - 0.234
2.570.600.354	7.430.800.341	100 - 34.4 - 8.41 - 1.54 - 0.234
2.570.600.354	7.430.800.341	100 - 34.4 - 8.41 - 1.54 - 0.234
2.570.600.354	7.430.800.341	100 - 34.4 - 8.41 - 1.54 - 0.234
2.570.600.354	7.430.800.341	100 - 34.4 - 8.41 - 1.54 - 0.234
2.570.600.354	7.430.800.341	100 - 34.4 - 8.41 - 1.54 - 0.234
2.570.600.354	7.430.800.341	100 - 34.4 - 8.41 - 1.54 - 0.234
2.570.600.354	7.430.800.341	100 - 34.4 - 8.41 - 1.54 - 0.234
2.570.600.354	7.430.800.341	100 - 34.4 - 8.41 - 1.54 - 0.234
2.570.600.354	7.430.800.341	100 - 34.4 - 8.41 - 1.54 - 0.234
2.570.600.354	7.430.800.341	100 - 34.4 - 8.41 - 1.54 - 0.234
2.570.600.354	7.430.800.341	100 - 34.4 - 8.41 - 1.54 - 0.234
2.570.600.354	7.430.800.341	100 - 34.4 - 8.41 - 1.54 - 0.234

2.570.600.354	7.430.800.341	100 - 34.4 - 8.41 - 1.54 - 0.234
2.570.600.354	7.430.800.341	100 - 34.4 - 8.41 - 1.54 - 0.234
2.570.600.354	7.430.800.341	100 - 34.4 - 8.41 - 1.54 - 0.234
2.570.600.354	7.430.800.341	100 - 34.4 - 8.41 - 1.54 - 0.234
2.570.600.354	7.430.800.341	100 - 34.4 - 8.41 - 1.54 - 0.234
2.570.600.354	7.430.800.341	100 - 34.4 - 8.41 - 1.54 - 0.234
2.570.600.354	7.430.800.341	100 - 34.4 - 8.41 - 1.54 - 0.234
2.570.600.354	7.430.800.341	100 - 34.4 - 8.41 - 1.54 - 0.234
2.570.600.354	7.430.800.341	100 - 34.4 - 8.41 - 1.54 - 0.234
2.570.600.354	7.430.800.341	100 - 34.4 - 8.41 - 1.54 - 0.234
2.570.600.354	7.430.800.341	100 - 34.4 - 8.41 - 1.54 - 0.234
2.570.600.354	7.430.800.341	100 - 34.4 - 8.41 - 1.54 - 0.234
2.570.600.354	7.430.800.341	100 - 34.4 - 8.41 - 1.54 - 0.234
2.570.600.354	7.430.800.341	100 - 34.4 - 8.41 - 1.54 - 0.234
2.570.600.354	7.430.800.341	100 - 34.4 - 8.41 - 1.54 - 0.234
2.570.600.354	7.430.800.341	100 - 34.4 - 8.41 - 1.54 - 0.234
2.570.600.354	7.430.800.341	100 - 34.4 - 8.41 - 1.54 - 0.234
2.570.600.354	7.430.800.341	100 - 34.4 - 8.41 - 1.54 - 0.234
2.570.600.354	7.430.800.341	100 - 34.4 - 8.41 - 1.54 - 0.234
2.570.600.354	7.430.800.341	100 - 34.4 - 8.41 - 1.54 - 0.234
2.570.600.354	7.430.800.341	100 - 34.4 - 8.41 - 1.54 - 0.234
2.570.600.354	7.430.800.341	100 - 34.4 - 8.41 - 1.54 - 0.234
2.570.600.354	7.430.800.341	100 - 34.4 - 8.41 - 1.54 - 0.234
2.570.600.354	7.430.800.341	100 - 34.4 - 8.41 - 1.54 - 0.234
2.570.600.354	7.430.800.341	100 - 34.4 - 8.41 - 1.54 - 0.234
2.570.600.354	7.430.800.341	100 - 34.4 - 8.41 - 1.54 - 0.234
184.533.574	6.915.731.428	100 - 34.4 - 8.41 - 1.54 - 0.234
184.533.574	6.915.731.428	100 - 34.4 - 8.41 - 1.54 - 0.234
184.533.574	6.915.731.428	100 - 34.4 - 8.41 - 1.54 - 0.234
184.533.574	6.915.731.428	100 - 34.4 - 8.41 - 1.54 - 0.234
184.533.574	6.915.731.428	100 - 34.4 - 8.41 - 1.54 - 0.234
184.533.574	6.915.731.428	100 - 34.4 - 8.41 - 1.54 - 0.234
184.533.574	6.915.731.428	100 - 34.4 - 8.41 - 1.54 - 0.234
184.533.574	6.915.731.428	100 - 34.4 - 8.41 - 1.54 - 0.234
184.533.574	6.915.731.428	100 - 34.4 - 8.41 - 1.54 - 0.234
184.533.574	6.915.731.428	100 - 34.4 - 8.41 - 1.54 - 0.234
184.533.574	6.915.731.428	100 - 34.4 - 8.41 - 1.54 - 0.234
184.533.574	6.915.731.428	100 - 34.4 - 8.41 - 1.54 - 0.234
184.533.574	6.915.731.428	100 - 34.4 - 8.41 - 1.54 - 0.234
184.533.574	6.915.731.428	100 - 34.4 - 8.41 - 1.54 - 0.234
184.533.574	6.915.731.428	100 - 34.4 - 8.41 - 1.54 - 0.234
184.533.574	6.915.731.428	100 - 34.4 - 8.41 - 1.54 - 0.234
184.533.574	6.915.731.428	100 - 34.4 - 8.41 - 1.54 - 0.234
184.533.574	6.915.731.428	100 - 34.4 - 8.41 - 1.54 - 0.234
184.533.574	6.915.731.428	100 - 34.4 - 8.41 - 1.54 - 0.234
184.533.574	6.915.731.428	100 - 34.4 - 8.41 - 1.54 - 0.234
184.533.574	6.915.731.428	100 - 34.4 - 8.41 - 1.54 - 0.234
184.533.574	6.915.731.428	100 - 34.4 - 8.41 - 1.54 - 0.234
184.533.574	6.915.731.428	100 - 34.4 - 8.41 - 1.54 - 0.234
184.533.574	6.915.731.428	100 - 34.4 - 8.41 - 1.54 - 0.234

184.533.574	6.915.731.428	100 - 34.4 - 8.41 - 1.54 - 0.234
184.533.574	6.915.731.428	100 - 34.4 - 8.41 - 1.54 - 0.234
184.533.574	6.915.731.428	100 - 34.4 - 8.41 - 1.54 - 0.234
184.533.574	6.915.731.428	100 - 34.4 - 8.41 - 1.54 - 0.234
184.533.574	6.915.731.428	100 - 34.4 - 8.41 - 1.54 - 0.234
184.533.574	6.915.731.428	100 - 34.4 - 8.41 - 1.54 - 0.234
184.533.574	6.915.731.428	100 - 34.4 - 8.41 - 1.54 - 0.234
184.533.574	6.915.731.428	100 - 34.4 - 8.41 - 1.54 - 0.234
184.533.574	6.915.731.428	100 - 34.4 - 8.41 - 1.54 - 0.234
184.533.574	6.915.731.428	100 - 34.4 - 8.41 - 1.54 - 0.234
184.533.574	6.915.731.428	100 - 34.4 - 8.41 - 1.54 - 0.234
184.533.574	6.915.731.428	100 - 34.4 - 8.41 - 1.54 - 0.234
184.533.574	6.915.731.428	100 - 34.4 - 8.41 - 1.54 - 0.234
184.533.574	6.915.731.428	100 - 34.4 - 8.41 - 1.54 - 0.234
184.533.574	6.915.731.428	100 - 34.4 - 8.41 - 1.54 - 0.234
184.533.574	6.915.731.428	100 - 34.4 - 8.41 - 1.54 - 0.234
184.533.574	6.915.731.428	100 - 34.4 - 8.41 - 1.54 - 0.234
184.533.574	6.915.731.428	100 - 34.4 - 8.41 - 1.54 - 0.234
184.533.574	6.915.731.428	100 - 34.4 - 8.41 - 1.54 - 0.234
184.533.574	6.915.731.428	100 - 34.4 - 8.41 - 1.54 - 0.234
184.533.574	6.915.731.428	100 - 34.4 - 8.41 - 1.54 - 0.234
184.533.574	6.915.731.428	100 - 34.4 - 8.41 - 1.54 - 0.234
184.533.574	6.915.731.428	100 - 34.4 - 8.41 - 1.54 - 0.234
184.533.574	6.915.731.428	100 - 34.4 - 8.41 - 1.54 - 0.234
184.533.574	6.915.731.428	100 - 34.4 - 8.41 - 1.54 - 0.234
184.533.574	6.915.731.428	100 - 34.4 - 8.41 - 1.54 - 0.234
184.533.574	6.915.731.428	100 - 34.4 - 8.41 - 1.54 - 0.234
184.533.574	6.915.731.428	100 - 34.4 - 8.41 - 1.54 - 0.234
184.533.574	6.915.731.428	100 - 34.4 - 8.41 - 1.54 - 0.234
184.533.574	6.915.731.428	100 - 34.4 - 8.41 - 1.54 - 0.234
184.533.574	6.915.731.428	100 - 34.4 - 8.41 - 1.54 - 0.234
184.533.574	6.915.731.428	100 - 34.4 - 8.41 - 1.54 - 0.234
3.247.836.587	7.885.945.044	100 - 22.9 - 3.53 - 0.408
3.247.836.587	7.885.945.044	100 - 22.9 - 3.53 - 0.408
-1.893.266.346	6.911.641.802	100 - 31.9 - 10.3 - 2.16 - 0.348
-5.961.570.828	9.250.660.137	100 - 7.06 - 1.04
-5.961.570.828	9.250.660.137	100 - 7.06 - 1.04
-5.961.570.828	9.250.660.137	100 - 7.06 - 1.04
2.486.487.164	7.396.874.886	100 - 26.4 - 7.26 - 1.3 - 0.219
2.486.487.164	7.396.874.886	100 - 26.4 - 7.26 - 1.3 - 0.219
2.570.600.354	7.430.800.341	100 - 34.4 - 8.41 - 1.54 - 0.234
2.570.600.354	7.430.800.341	100 - 34.4 - 8.41 - 1.54 - 0.234
2.570.600.354	7.430.800.341	100 - 34.4 - 8.41 - 1.54 - 0.234
2.570.600.354	7.430.800.341	100 - 34.4 - 8.41 - 1.54 - 0.234
2.570.600.354	7.430.800.341	100 - 34.4 - 8.41 - 1.54 - 0.234
2.570.600.354	7.430.800.341	100 - 34.4 - 8.41 - 1.54 - 0.234
2.570.600.354	7.430.800.341	100 - 34.4 - 8.41 - 1.54 - 0.234
2.570.600.354	7.430.800.341	100 - 34.4 - 8.41 - 1.54 - 0.234
2.570.600.354	7.430.800.341	100 - 34.4 - 8.41 - 1.54 - 0.234
2.570.600.354	7.430.800.341	100 - 34.4 - 8.41 - 1.54 - 0.234

2.570.600.354	7.430.800.341	100 - 34.4 - 8.41 - 1.54 - 0.234
2.570.600.354	7.430.800.341	100 - 34.4 - 8.41 - 1.54 - 0.234
2.570.600.354	7.430.800.341	100 - 34.4 - 8.41 - 1.54 - 0.234
2.570.600.354	7.430.800.341	100 - 34.4 - 8.41 - 1.54 - 0.234
2.570.600.354	7.430.800.341	100 - 34.4 - 8.41 - 1.54 - 0.234
2.570.600.354	7.430.800.341	100 - 34.4 - 8.41 - 1.54 - 0.234
2.570.600.354	7.430.800.341	100 - 34.4 - 8.41 - 1.54 - 0.234
2.570.600.354	7.430.800.341	100 - 34.4 - 8.41 - 1.54 - 0.234
2.570.600.354	7.430.800.341	100 - 34.4 - 8.41 - 1.54 - 0.234
2.570.600.354	7.430.800.341	100 - 34.4 - 8.41 - 1.54 - 0.234
2.570.600.354	7.430.800.341	100 - 34.4 - 8.41 - 1.54 - 0.234
2.570.600.354	7.430.800.341	100 - 34.4 - 8.41 - 1.54 - 0.234
2.570.600.354	7.430.800.341	100 - 34.4 - 8.41 - 1.54 - 0.234
2.570.600.354	7.430.800.341	100 - 34.4 - 8.41 - 1.54 - 0.234
2.570.600.354	7.430.800.341	100 - 34.4 - 8.41 - 1.54 - 0.234
2.570.600.354	7.430.800.341	100 - 34.4 - 8.41 - 1.54 - 0.234
2.570.600.354	7.430.800.341	100 - 34.4 - 8.41 - 1.54 - 0.234
2.570.600.354	7.430.800.341	100 - 34.4 - 8.41 - 1.54 - 0.234
2.570.600.354	7.430.800.341	100 - 34.4 - 8.41 - 1.54 - 0.234
2.570.600.354	7.430.800.341	100 - 34.4 - 8.41 - 1.54 - 0.234
2.570.600.354	7.430.800.341	100 - 34.4 - 8.41 - 1.54 - 0.234
2.570.600.354	7.430.800.341	100 - 34.4 - 8.41 - 1.54 - 0.234
2.570.600.354	7.430.800.341	100 - 34.4 - 8.41 - 1.54 - 0.234
2.570.600.354	7.430.800.341	100 - 34.4 - 8.41 - 1.54 - 0.234
2.570.600.354	7.430.800.341	100 - 34.4 - 8.41 - 1.54 - 0.234
2.570.600.354	7.430.800.341	100 - 34.4 - 8.41 - 1.54 - 0.234
2.570.600.354	7.430.800.341	100 - 34.4 - 8.41 - 1.54 - 0.234
2.570.600.354	7.430.800.341	100 - 34.4 - 8.41 - 1.54 - 0.234
2.570.600.354	7.430.800.341	100 - 34.4 - 8.41 - 1.54 - 0.234
2.570.600.354	7.430.800.341	100 - 34.4 - 8.41 - 1.54 - 0.234
2.570.600.354	7.430.800.341	100 - 34.4 - 8.41 - 1.54 - 0.234
2.570.600.354	7.430.800.341	100 - 34.4 - 8.41 - 1.54 - 0.234
2.570.600.354	7.430.800.341	100 - 34.4 - 8.41 - 1.54 - 0.234
2.570.600.354	7.430.800.341	100 - 34.4 - 8.41 - 1.54 - 0.234
2.570.600.354	7.430.800.341	100 - 34.4 - 8.41 - 1.54 - 0.234
2.570.600.354	7.430.800.341	100 - 34.4 - 8.41 - 1.54 - 0.234
2.570.600.354	7.430.800.341	100 - 34.4 - 8.41 - 1.54 - 0.234
2.570.600.354	7.430.800.341	100 - 34.4 - 8.41 - 1.54 - 0.234
2.570.600.354	7.430.800.341	100 - 34.4 - 8.41 - 1.54 - 0.234
2.570.600.354	7.430.800.341	100 - 34.4 - 8.41 - 1.54 - 0.234
2.570.600.354	7.430.800.341	100 - 34.4 - 8.41 - 1.54 - 0.234
2.570.600.354	7.430.800.341	100 - 34.4 - 8.41 - 1.54 - 0.234
2.570.600.354	7.430.800.341	100 - 34.4 - 8.41 - 1.54 - 0.234
2.570.600.354	7.430.800.341	100 - 34.4 - 8.41 - 1.54 - 0.234
2.570.600.354	7.430.800.341	100 - 34.4 - 8.41 - 1.54 - 0.234
2.570.600.354	7.430.800.341	100 - 34.4 - 8.41 - 1.54 - 0.234
184.533.574	6.915.731.428	100 - 34.4 - 8.41 - 1.54 - 0.234
184.533.574	6.915.731.428	100 - 34.4 - 8.41 - 1.54 - 0.234
184.533.574	6.915.731.428	100 - 34.4 - 8.41 - 1.54 - 0.234
184.533.574	6.915.731.428	100 - 34.4 - 8.41 - 1.54 - 0.234

184.533.574	6.915.731.428	100 - 34.4 - 8.41 - 1.54 - 0.234
184.533.574	6.915.731.428	100 - 34.4 - 8.41 - 1.54 - 0.234
184.533.574	6.915.731.428	100 - 34.4 - 8.41 - 1.54 - 0.234
184.533.574	6.915.731.428	100 - 34.4 - 8.41 - 1.54 - 0.234
184.533.574	6.915.731.428	100 - 34.4 - 8.41 - 1.54 - 0.234
184.533.574	6.915.731.428	100 - 34.4 - 8.41 - 1.54 - 0.234
184.533.574	6.915.731.428	100 - 34.4 - 8.41 - 1.54 - 0.234
184.533.574	6.915.731.428	100 - 34.4 - 8.41 - 1.54 - 0.234
184.533.574	6.915.731.428	100 - 34.4 - 8.41 - 1.54 - 0.234
184.533.574	6.915.731.428	100 - 34.4 - 8.41 - 1.54 - 0.234
184.533.574	6.915.731.428	100 - 34.4 - 8.41 - 1.54 - 0.234
184.533.574	6.915.731.428	100 - 34.4 - 8.41 - 1.54 - 0.234
184.533.574	6.915.731.428	100 - 34.4 - 8.41 - 1.54 - 0.234
184.533.574	6.915.731.428	100 - 34.4 - 8.41 - 1.54 - 0.234
184.533.574	6.915.731.428	100 - 34.4 - 8.41 - 1.54 - 0.234
184.533.574	6.915.731.428	100 - 34.4 - 8.41 - 1.54 - 0.234
184.533.574	6.915.731.428	100 - 34.4 - 8.41 - 1.54 - 0.234
184.533.574	6.915.731.428	100 - 34.4 - 8.41 - 1.54 - 0.234
184.533.574	6.915.731.428	100 - 34.4 - 8.41 - 1.54 - 0.234
184.533.574	6.915.731.428	100 - 34.4 - 8.41 - 1.54 - 0.234
184.533.574	6.915.731.428	100 - 34.4 - 8.41 - 1.54 - 0.234
184.533.574	6.915.731.428	100 - 34.4 - 8.41 - 1.54 - 0.234
184.533.574	6.915.731.428	100 - 34.4 - 8.41 - 1.54 - 0.234
184.533.574	6.915.731.428	100 - 34.4 - 8.41 - 1.54 - 0.234
184.533.574	6.915.731.428	100 - 34.4 - 8.41 - 1.54 - 0.234
184.533.574	6.915.731.428	100 - 34.4 - 8.41 - 1.54 - 0.234
184.533.574	6.915.731.428	100 - 34.4 - 8.41 - 1.54 - 0.234
184.533.574	6.915.731.428	100 - 34.4 - 8.41 - 1.54 - 0.234
184.533.574	6.915.731.428	100 - 34.4 - 8.41 - 1.54 - 0.234
184.533.574	6.915.731.428	100 - 34.4 - 8.41 - 1.54 - 0.234
184.533.574	6.915.731.428	100 - 34.4 - 8.41 - 1.54 - 0.234
184.533.574	6.915.731.428	100 - 34.4 - 8.41 - 1.54 - 0.234
184.533.574	6.915.731.428	100 - 34.4 - 8.41 - 1.54 - 0.234
184.533.574	6.915.731.428	100 - 34.4 - 8.41 - 1.54 - 0.234
184.533.574	6.915.731.428	100 - 34.4 - 8.41 - 1.54 - 0.234
184.533.574	6.915.731.428	100 - 34.4 - 8.41 - 1.54 - 0.234
184.533.574	6.915.731.428	100 - 34.4 - 8.41 - 1.54 - 0.234
184.533.574	6.915.731.428	100 - 34.4 - 8.41 - 1.54 - 0.234
184.533.574	6.915.731.428	100 - 34.4 - 8.41 - 1.54 - 0.234
184.533.574	6.915.731.428	100 - 34.4 - 8.41 - 1.54 - 0.234
184.533.574	6.915.731.428	100 - 34.4 - 8.41 - 1.54 - 0.234
184.533.574	6.915.731.428	100 - 34.4 - 8.41 - 1.54 - 0.234
184.533.574	6.915.731.428	100 - 34.4 - 8.41 - 1.54 - 0.234
184.533.574	6.915.731.428	100 - 34.4 - 8.41 - 1.54 - 0.234
184.533.574	6.915.731.428	100 - 34.4 - 8.41 - 1.54 - 0.234
184.533.574	6.915.731.428	100 - 34.4 - 8.41 - 1.54 - 0.234
184.533.574	6.915.731.428	100 - 34.4 - 8.41 - 1.54 - 0.234
184.533.574	6.915.731.428	100 - 34.4 - 8.41 - 1.54 - 0.234
184.533.574	6.915.731.428	100 - 34.4 - 8.41 - 1.54 - 0.234
184.533.574	6.915.731.428	100 - 34.4 - 8.41 - 1.54 - 0.234

184.533.574	6.915.731.428	100 - 34.4 - 8.41 - 1.54 - 0.234
184.533.574	6.915.731.428	100 - 34.4 - 8.41 - 1.54 - 0.234
3.247.836.587	7.885.945.044	100 - 22.9 - 3.53 - 0.408
3.247.836.587	7.885.945.044	100 - 22.9 - 3.53 - 0.408
-1.893.266.346	6.911.641.802	100 - 31.9 - 10.3 - 2.16 - 0.348

Link
http://nonlinear.com/redirect/outbound?p=hmdb&param=HMDB0038329
http://nonlinear.com/redirect/outbound?p=hmdb&param=HMDB0038329
http://nonlinear.com/redirect/outbound?p=hmdb&param=HMDB0006592
http://nonlinear.com/redirect/outbound?p=hmdb&param=HMDB0039750
http://nonlinear.com/redirect/outbound?p=hmdb&param=HMDB0041622
http://nonlinear.com/redirect/outbound?p=hmdb&param=HMDB0006592
http://nonlinear.com/redirect/outbound?p=hmdb&param=HMDB0039750
http://nonlinear.com/redirect/outbound?p=hmdb&param=HMDB0041622
http://nonlinear.com/redirect/outbound?p=hmdb&param=HMDB0128725
http://nonlinear.com/redirect/outbound?p=hmdb&param=HMDB0128726
http://nonlinear.com/redirect/outbound?p=hmdb&param=HMDB0128727
http://nonlinear.com/redirect/outbound?p=hmdb&param=HMDB0128728
http://nonlinear.com/redirect/outbound?p=hmdb&param=HMDB0128725
http://nonlinear.com/redirect/outbound?p=hmdb&param=HMDB0128726
http://nonlinear.com/redirect/outbound?p=hmdb&param=HMDB0128727
http://nonlinear.com/redirect/outbound?p=hmdb&param=HMDB0128728
http://nonlinear.com/redirect/outbound?p=hmdb&param=HMDB0000797
http://nonlinear.com/redirect/outbound?p=hmdb&param=HMDB0000797
http://nonlinear.com/redirect/outbound?p=hmdb&param=HMDB0030112
http://nonlinear.com/redirect/outbound?p=hmdb&param=HMDB0035964
http://nonlinear.com/redirect/outbound?p=hmdb&param=HMDB0126375
http://nonlinear.com/redirect/outbound?p=hmdb&param=HMDB0015075
http://nonlinear.com/redirect/outbound?p=hmdb&param=HMDB0015591
http://nonlinear.com/redirect/outbound?p=hmdb&param=HMDB0030112
http://nonlinear.com/redirect/outbound?p=hmdb&param=HMDB0035964
http://nonlinear.com/redirect/outbound?p=hmdb&param=HMDB0126375
http://nonlinear.com/redirect/outbound?p=hmdb&param=HMDB0015075
http://nonlinear.com/redirect/outbound?p=hmdb&param=HMDB0015591
http://nonlinear.com/redirect/outbound?p=hmdb&param=HMDB0006607
http://nonlinear.com/redirect/outbound?p=hmdb&param=HMDB0041621
http://nonlinear.com/redirect/outbound?p=hmdb&param=HMDB0006607
http://nonlinear.com/redirect/outbound?p=hmdb&param=HMDB0041621
http://nonlinear.com/redirect/outbound?p=hmdb&param=HMDB0014876
http://nonlinear.com/redirect/outbound?p=hmdb&param=HMDB0014876
http://nonlinear.com/redirect/outbound?p=hmdb&param=HMDB0015121
http://nonlinear.com/redirect/outbound?p=hmdb&param=HMDB0015121
http://nonlinear.com/redirect/outbound?p=hmdb&param=HMDB0000043
http://nonlinear.com/redirect/outbound?p=hmdb&param=HMDB0000883
http://nonlinear.com/redirect/outbound?p=hmdb&param=HMDB0001382
http://nonlinear.com/redirect/outbound?p=hmdb&param=HMDB0002141
http://nonlinear.com/redirect/outbound?p=hmdb&param=HMDB0003355
http://nonlinear.com/redirect/outbound?p=hmdb&param=HMDB0013716
http://nonlinear.com/redirect/outbound?p=hmdb&param=HMDB0015550
http://nonlinear.com/redirect/outbound?p=hmdb&param=HMDB0000043
http://nonlinear.com/redirect/outbound?p=hmdb&param=HMDB0000883
http://nonlinear.com/redirect/outbound?p=hmdb&param=HMDB0001382
http://nonlinear.com/redirect/outbound?p=hmdb&param=HMDB0001396
http://nonlinear.com/redirect/outbound?p=hmdb&param=HMDB0014832

http://nonlinear.com/redirect/outbound?p=hmdb&param=HMDB0041884
http://nonlinear.com/redirect/outbound?p=hmdb&param=HMDB0060823
http://nonlinear.com/redirect/outbound?p=hmdb&param=HMDB0011496
http://nonlinear.com/redirect/outbound?p=hmdb&param=HMDB0011526
http://nonlinear.com/redirect/outbound?p=hmdb&param=HMDB0011496
http://nonlinear.com/redirect/outbound?p=hmdb&param=HMDB0011526
http://nonlinear.com/redirect/outbound?p=hmdb&param=HMDB0130483
http://nonlinear.com/redirect/outbound?p=hmdb&param=HMDB0130484
http://nonlinear.com/redirect/outbound?p=hmdb&param=HMDB0130485
http://nonlinear.com/redirect/outbound?p=hmdb&param=HMDB0029776
http://nonlinear.com/redirect/outbound?p=hmdb&param=HMDB0130483
http://nonlinear.com/redirect/outbound?p=hmdb&param=HMDB0130484
http://nonlinear.com/redirect/outbound?p=hmdb&param=HMDB0130485
http://nonlinear.com/redirect/outbound?p=hmdb&param=HMDB0029776
http://nonlinear.com/redirect/outbound?p=hmdb&param=HMDB0132953
http://nonlinear.com/redirect/outbound?p=hmdb&param=HMDB0132954
http://nonlinear.com/redirect/outbound?p=hmdb&param=HMDB0132953
http://nonlinear.com/redirect/outbound?p=hmdb&param=HMDB0132954
http://nonlinear.com/redirect/outbound?p=hmdb&param=HMDB0015230
http://nonlinear.com/redirect/outbound?p=hmdb&param=HMDB0029850
http://nonlinear.com/redirect/outbound?p=hmdb&param=HMDB0032368
http://nonlinear.com/redirect/outbound?p=hmdb&param=HMDB0001197
http://nonlinear.com/redirect/outbound?p=hmdb&param=HMDB0032114
http://nonlinear.com/redirect/outbound?p=hmdb&param=HMDB0006607
http://nonlinear.com/redirect/outbound?p=hmdb&param=HMDB0041621
http://nonlinear.com/redirect/outbound?p=hmdb&param=HMDB0126885
http://nonlinear.com/redirect/outbound?p=hmdb&param=HMDB0126886
http://nonlinear.com/redirect/outbound?p=hmdb&param=HMDB0126887
http://nonlinear.com/redirect/outbound?p=hmdb&param=HMDB0126888
http://nonlinear.com/redirect/outbound?p=hmdb&param=HMDB0127450
http://nonlinear.com/redirect/outbound?p=hmdb&param=HMDB0127451
http://nonlinear.com/redirect/outbound?p=hmdb&param=HMDB0126885
http://nonlinear.com/redirect/outbound?p=hmdb&param=HMDB0126886
http://nonlinear.com/redirect/outbound?p=hmdb&param=HMDB0126887
http://nonlinear.com/redirect/outbound?p=hmdb&param=HMDB0126888
http://nonlinear.com/redirect/outbound?p=hmdb&param=HMDB0127450
http://nonlinear.com/redirect/outbound?p=hmdb&param=HMDB0127451
http://nonlinear.com/redirect/outbound?p=hmdb&param=HMDB0127452
http://nonlinear.com/redirect/outbound?p=hmdb&param=HMDB0060316
http://nonlinear.com/redirect/outbound?p=hmdb&param=HMDB0129666
http://nonlinear.com/redirect/outbound?p=hmdb&param=HMDB0129667
http://nonlinear.com/redirect/outbound?p=hmdb&param=HMDB0129668
http://nonlinear.com/redirect/outbound?p=hmdb&param=HMDB0060316
http://nonlinear.com/redirect/outbound?p=hmdb&param=HMDB0129666
http://nonlinear.com/redirect/outbound?p=hmdb&param=HMDB0129667
http://nonlinear.com/redirect/outbound?p=hmdb&param=HMDB0129668
http://nonlinear.com/redirect/outbound?p=hmdb&param=HMDB0006595
http://nonlinear.com/redirect/outbound?p=hmdb&param=HMDB0032114
http://nonlinear.com/redirect/outbound?p=hmdb&param=HMDB0034634
http://nonlinear.com/redirect/outbound?p=hmdb&param=HMDB0125188

http://nonlinear.com/redirect/outbound?p=hmdb&param=HMDB0125189
http://nonlinear.com/redirect/outbound?p=hmdb&param=HMDB0125190
http://nonlinear.com/redirect/outbound?p=hmdb&param=HMDB0125191
http://nonlinear.com/redirect/outbound?p=hmdb&param=HMDB0125192
http://nonlinear.com/redirect/outbound?p=hmdb&param=HMDB0125193
http://nonlinear.com/redirect/outbound?p=hmdb&param=HMDB0125188
http://nonlinear.com/redirect/outbound?p=hmdb&param=HMDB0125189
http://nonlinear.com/redirect/outbound?p=hmdb&param=HMDB0125190
http://nonlinear.com/redirect/outbound?p=hmdb&param=HMDB0125191
http://nonlinear.com/redirect/outbound?p=hmdb&param=HMDB0125192
http://nonlinear.com/redirect/outbound?p=hmdb&param=HMDB0125193
http://nonlinear.com/redirect/outbound?p=hmdb&param=HMDB0034634
http://nonlinear.com/redirect/outbound?p=hmdb&param=HMDB0014469
http://nonlinear.com/redirect/outbound?p=hmdb&param=HMDB0014687
http://nonlinear.com/redirect/outbound?p=hmdb&param=HMDB0033843
http://nonlinear.com/redirect/outbound?p=hmdb&param=HMDB0034634
http://nonlinear.com/redirect/outbound?p=hmdb&param=HMDB0041730
http://nonlinear.com/redirect/outbound?p=hmdb&param=HMDB0000972
http://nonlinear.com/redirect/outbound?p=hmdb&param=HMDB0001562
http://nonlinear.com/redirect/outbound?p=hmdb&param=HMDB0002140
http://nonlinear.com/redirect/outbound?p=hmdb&param=HMDB0062757
http://nonlinear.com/redirect/outbound?p=hmdb&param=HMDB0037373
http://nonlinear.com/redirect/outbound?p=hmdb&param=HMDB0128696
http://nonlinear.com/redirect/outbound?p=hmdb&param=HMDB0060139
http://nonlinear.com/redirect/outbound?p=hmdb&param=HMDB0060574
http://nonlinear.com/redirect/outbound?p=hmdb&param=HMDB0031112
http://nonlinear.com/redirect/outbound?p=hmdb&param=HMDB0034788
http://nonlinear.com/redirect/outbound?p=hmdb&param=HMDB0040769
http://nonlinear.com/redirect/outbound?p=hmdb&param=HMDB0040769
http://nonlinear.com/redirect/outbound?p=hmdb&param=HMDB0040387
http://nonlinear.com/redirect/outbound?p=hmdb&param=HMDB0000191
http://nonlinear.com/redirect/outbound?p=hmdb&param=HMDB0006483
http://nonlinear.com/redirect/outbound?p=hmdb&param=HMDB0011753
http://nonlinear.com/redirect/outbound?p=hmdb&param=HMDB0062186
http://nonlinear.com/redirect/outbound?p=hmdb&param=HMDB0062501
http://nonlinear.com/redirect/outbound?p=hmdb&param=HMDB0040387
http://nonlinear.com/redirect/outbound?p=hmdb&param=HMDB0015481
http://nonlinear.com/redirect/outbound?p=hmdb&param=HMDB0000197
http://nonlinear.com/redirect/outbound?p=hmdb&param=HMDB0004073
http://nonlinear.com/redirect/outbound?p=hmdb&param=HMDB0038628
http://nonlinear.com/redirect/outbound?p=hmdb&param=HMDB0040972
http://nonlinear.com/redirect/outbound?p=hmdb&param=HMDB0015481
http://nonlinear.com/redirect/outbound?p=hmdb&param=HMDB0000197
http://nonlinear.com/redirect/outbound?p=hmdb&param=HMDB0004073
http://nonlinear.com/redirect/outbound?p=hmdb&param=HMDB0038628
http://nonlinear.com/redirect/outbound?p=hmdb&param=HMDB0040972
http://nonlinear.com/redirect/outbound?p=hmdb&param=HMDB0034773
http://nonlinear.com/redirect/outbound?p=hmdb&param=HMDB0034774
http://nonlinear.com/redirect/outbound?p=hmdb&param=HMDB0030112
http://nonlinear.com/redirect/outbound?p=hmdb&param=HMDB0035964

http://nonlinear.com/redirect/outbound?p=hmdb&param=HMDB0126375
http://nonlinear.com/redirect/outbound?p=hmdb&param=HMDB0134078
http://nonlinear.com/redirect/outbound?p=hmdb&param=HMDB0034773
http://nonlinear.com/redirect/outbound?p=hmdb&param=HMDB0034774
http://nonlinear.com/redirect/outbound?p=hmdb&param=HMDB0030112
http://nonlinear.com/redirect/outbound?p=hmdb&param=HMDB0035964
http://nonlinear.com/redirect/outbound?p=hmdb&param=HMDB0126375
http://nonlinear.com/redirect/outbound?p=hmdb&param=HMDB0134078
http://nonlinear.com/redirect/outbound?p=hmdb&param=HMDB0030627
http://nonlinear.com/redirect/outbound?p=hmdb&param=HMDB0034281
http://nonlinear.com/redirect/outbound?p=hmdb&param=HMDB0135503
http://nonlinear.com/redirect/outbound?p=hmdb&param=HMDB0135505
http://nonlinear.com/redirect/outbound?p=hmdb&param=HMDB0135507
http://nonlinear.com/redirect/outbound?p=hmdb&param=HMDB0041431
http://nonlinear.com/redirect/outbound?p=hmdb&param=HMDB0062189
http://nonlinear.com/redirect/outbound?p=hmdb&param=HMDB0030627
http://nonlinear.com/redirect/outbound?p=hmdb&param=HMDB0034281
http://nonlinear.com/redirect/outbound?p=hmdb&param=HMDB0135503
http://nonlinear.com/redirect/outbound?p=hmdb&param=HMDB0135505
http://nonlinear.com/redirect/outbound?p=hmdb&param=HMDB0135507
http://nonlinear.com/redirect/outbound?p=hmdb&param=HMDB0041431
http://nonlinear.com/redirect/outbound?p=hmdb&param=HMDB0062189
http://nonlinear.com/redirect/outbound?p=hmdb&param=HMDB0129415
http://nonlinear.com/redirect/outbound?p=hmdb&param=HMDB0129416
http://nonlinear.com/redirect/outbound?p=hmdb&param=HMDB0129417
http://nonlinear.com/redirect/outbound?p=hmdb&param=HMDB0132767
http://nonlinear.com/redirect/outbound?p=hmdb&param=HMDB0132768
http://nonlinear.com/redirect/outbound?p=hmdb&param=HMDB0132770
http://nonlinear.com/redirect/outbound?p=hmdb&param=HMDB0132771
http://nonlinear.com/redirect/outbound?p=hmdb&param=HMDB0132772
http://nonlinear.com/redirect/outbound?p=hmdb&param=HMDB0132774
http://nonlinear.com/redirect/outbound?p=hmdb&param=HMDB0132775
http://nonlinear.com/redirect/outbound?p=hmdb&param=HMDB0132776
http://nonlinear.com/redirect/outbound?p=hmdb&param=HMDB0132777
http://nonlinear.com/redirect/outbound?p=hmdb&param=HMDB0132778
http://nonlinear.com/redirect/outbound?p=hmdb&param=HMDB0132779
http://nonlinear.com/redirect/outbound?p=hmdb&param=HMDB0132780
http://nonlinear.com/redirect/outbound?p=hmdb&param=HMDB0132782
http://nonlinear.com/redirect/outbound?p=hmdb&param=HMDB0132783
http://nonlinear.com/redirect/outbound?p=hmdb&param=HMDB0132784
http://nonlinear.com/redirect/outbound?p=hmdb&param=HMDB0132786
http://nonlinear.com/redirect/outbound?p=hmdb&param=HMDB0132787
http://nonlinear.com/redirect/outbound?p=hmdb&param=HMDB0132788
http://nonlinear.com/redirect/outbound?p=hmdb&param=HMDB0132789
http://nonlinear.com/redirect/outbound?p=hmdb&param=HMDB0132791
http://nonlinear.com/redirect/outbound?p=hmdb&param=HMDB0132792
http://nonlinear.com/redirect/outbound?p=hmdb&param=HMDB0132793
http://nonlinear.com/redirect/outbound?p=hmdb&param=HMDB0132811
http://nonlinear.com/redirect/outbound?p=hmdb&param=HMDB0132812
http://nonlinear.com/redirect/outbound?p=hmdb&param=HMDB0132813

http://nonlinear.com/redirect/outbound?p=hmdb&param=HMDB0132815
http://nonlinear.com/redirect/outbound?p=hmdb&param=HMDB0132816
http://nonlinear.com/redirect/outbound?p=hmdb&param=HMDB0132817
http://nonlinear.com/redirect/outbound?p=hmdb&param=HMDB0132819
http://nonlinear.com/redirect/outbound?p=hmdb&param=HMDB0132820
http://nonlinear.com/redirect/outbound?p=hmdb&param=HMDB0132821
http://nonlinear.com/redirect/outbound?p=hmdb&param=HMDB0132822
http://nonlinear.com/redirect/outbound?p=hmdb&param=HMDB0132823
http://nonlinear.com/redirect/outbound?p=hmdb&param=HMDB0132824
http://nonlinear.com/redirect/outbound?p=hmdb&param=HMDB0132825
http://nonlinear.com/redirect/outbound?p=hmdb&param=HMDB0132828
http://nonlinear.com/redirect/outbound?p=hmdb&param=HMDB0132831
http://nonlinear.com/redirect/outbound?p=hmdb&param=HMDB0132832
http://nonlinear.com/redirect/outbound?p=hmdb&param=HMDB0132833
http://nonlinear.com/redirect/outbound?p=hmdb&param=HMDB0132835
http://nonlinear.com/redirect/outbound?p=hmdb&param=HMDB0132836
http://nonlinear.com/redirect/outbound?p=hmdb&param=HMDB0132837
http://nonlinear.com/redirect/outbound?p=hmdb&param=HMDB0132838
http://nonlinear.com/redirect/outbound?p=hmdb&param=HMDB0132839
http://nonlinear.com/redirect/outbound?p=hmdb&param=HMDB0132840
http://nonlinear.com/redirect/outbound?p=hmdb&param=HMDB0132841
http://nonlinear.com/redirect/outbound?p=hmdb&param=HMDB0132842
http://nonlinear.com/redirect/outbound?p=hmdb&param=HMDB0132843
http://nonlinear.com/redirect/outbound?p=hmdb&param=HMDB0132844
http://nonlinear.com/redirect/outbound?p=hmdb&param=HMDB0132845
http://nonlinear.com/redirect/outbound?p=hmdb&param=HMDB0132846
http://nonlinear.com/redirect/outbound?p=hmdb&param=HMDB0132847
http://nonlinear.com/redirect/outbound?p=hmdb&param=HMDB0132848
http://nonlinear.com/redirect/outbound?p=hmdb&param=HMDB0031149
http://nonlinear.com/redirect/outbound?p=hmdb&param=HMDB0030627
http://nonlinear.com/redirect/outbound?p=hmdb&param=HMDB0034281
http://nonlinear.com/redirect/outbound?p=hmdb&param=HMDB0030627
http://nonlinear.com/redirect/outbound?p=hmdb&param=HMDB0034281
http://nonlinear.com/redirect/outbound?p=hmdb&param=HMDB0001337
http://nonlinear.com/redirect/outbound?p=hmdb&param=HMDB0005081
http://nonlinear.com/redirect/outbound?p=hmdb&param=HMDB0006027
http://nonlinear.com/redirect/outbound?p=hmdb&param=HMDB0010202
http://nonlinear.com/redirect/outbound?p=hmdb&param=HMDB0010205
http://nonlinear.com/redirect/outbound?p=hmdb&param=HMDB0010209
http://nonlinear.com/redirect/outbound?p=hmdb&param=HMDB0010210
http://nonlinear.com/redirect/outbound?p=hmdb&param=HMDB0010212
http://nonlinear.com/redirect/outbound?p=hmdb&param=HMDB0010217
http://nonlinear.com/redirect/outbound?p=hmdb&param=HMDB0012534
http://nonlinear.com/redirect/outbound?p=hmdb&param=HMDB0012611
http://nonlinear.com/redirect/outbound?p=hmdb&param=HMDB0013633
http://nonlinear.com/redirect/outbound?p=hmdb&param=HMDB0030101
http://nonlinear.com/redirect/outbound?p=hmdb&param=HMDB0035116
http://nonlinear.com/redirect/outbound?p=hmdb&param=HMDB0035311
http://nonlinear.com/redirect/outbound?p=hmdb&param=HMDB0036698
http://nonlinear.com/redirect/outbound?p=hmdb&param=HMDB0036706

http://nonlinear.com/redirect/outbound?p=hmdb&param=HMDB0036712
http://nonlinear.com/redirect/outbound?p=hmdb&param=HMDB0036713
http://nonlinear.com/redirect/outbound?p=hmdb&param=HMDB0036724
http://nonlinear.com/redirect/outbound?p=hmdb&param=HMDB0036752
http://nonlinear.com/redirect/outbound?p=hmdb&param=HMDB0036776
http://nonlinear.com/redirect/outbound?p=hmdb&param=HMDB0036806
http://nonlinear.com/redirect/outbound?p=hmdb&param=HMDB0036839
http://nonlinear.com/redirect/outbound?p=hmdb&param=HMDB0036930
http://nonlinear.com/redirect/outbound?p=hmdb&param=HMDB0037523
http://nonlinear.com/redirect/outbound?p=hmdb&param=HMDB0038521
http://nonlinear.com/redirect/outbound?p=hmdb&param=HMDB0039012
http://nonlinear.com/redirect/outbound?p=hmdb&param=HMDB0039716
http://nonlinear.com/redirect/outbound?p=hmdb&param=HMDB0041058
http://nonlinear.com/redirect/outbound?p=hmdb&param=HMDB0060053
http://nonlinear.com/redirect/outbound?p=hmdb&param=HMDB0061114
http://nonlinear.com/redirect/outbound?p=hmdb&param=HMDB0062222
http://nonlinear.com/redirect/outbound?p=hmdb&param=HMDB0062293
http://nonlinear.com/redirect/outbound?p=hmdb&param=HMDB0062296
http://nonlinear.com/redirect/outbound?p=hmdb&param=HMDB0062408
http://nonlinear.com/redirect/outbound?p=hmdb&param=HMDB0137419
http://nonlinear.com/redirect/outbound?p=hmdb&param=HMDB0129415
http://nonlinear.com/redirect/outbound?p=hmdb&param=HMDB0129416
http://nonlinear.com/redirect/outbound?p=hmdb&param=HMDB0129417
http://nonlinear.com/redirect/outbound?p=hmdb&param=HMDB0132767
http://nonlinear.com/redirect/outbound?p=hmdb&param=HMDB0132768
http://nonlinear.com/redirect/outbound?p=hmdb&param=HMDB0132770
http://nonlinear.com/redirect/outbound?p=hmdb&param=HMDB0132771
http://nonlinear.com/redirect/outbound?p=hmdb&param=HMDB0132772
http://nonlinear.com/redirect/outbound?p=hmdb&param=HMDB0132774
http://nonlinear.com/redirect/outbound?p=hmdb&param=HMDB0132775
http://nonlinear.com/redirect/outbound?p=hmdb&param=HMDB0132776
http://nonlinear.com/redirect/outbound?p=hmdb&param=HMDB0132777
http://nonlinear.com/redirect/outbound?p=hmdb&param=HMDB0132778
http://nonlinear.com/redirect/outbound?p=hmdb&param=HMDB0132779
http://nonlinear.com/redirect/outbound?p=hmdb&param=HMDB0132780
http://nonlinear.com/redirect/outbound?p=hmdb&param=HMDB0132782
http://nonlinear.com/redirect/outbound?p=hmdb&param=HMDB0132783
http://nonlinear.com/redirect/outbound?p=hmdb&param=HMDB0132784
http://nonlinear.com/redirect/outbound?p=hmdb&param=HMDB0132786
http://nonlinear.com/redirect/outbound?p=hmdb&param=HMDB0132787
http://nonlinear.com/redirect/outbound?p=hmdb&param=HMDB0132788
http://nonlinear.com/redirect/outbound?p=hmdb&param=HMDB0132789
http://nonlinear.com/redirect/outbound?p=hmdb&param=HMDB0132791
http://nonlinear.com/redirect/outbound?p=hmdb&param=HMDB0132792
http://nonlinear.com/redirect/outbound?p=hmdb&param=HMDB0132793
http://nonlinear.com/redirect/outbound?p=hmdb&param=HMDB0132811
http://nonlinear.com/redirect/outbound?p=hmdb&param=HMDB0132812
http://nonlinear.com/redirect/outbound?p=hmdb&param=HMDB0132813
http://nonlinear.com/redirect/outbound?p=hmdb&param=HMDB0132815
http://nonlinear.com/redirect/outbound?p=hmdb&param=HMDB0132816

http://nonlinear.com/redirect/outbound?p=hmdb&param=HMDB0132817
http://nonlinear.com/redirect/outbound?p=hmdb&param=HMDB0132819
http://nonlinear.com/redirect/outbound?p=hmdb&param=HMDB0132820
http://nonlinear.com/redirect/outbound?p=hmdb&param=HMDB0132821
http://nonlinear.com/redirect/outbound?p=hmdb&param=HMDB0132822
http://nonlinear.com/redirect/outbound?p=hmdb&param=HMDB0132823
http://nonlinear.com/redirect/outbound?p=hmdb&param=HMDB0132824
http://nonlinear.com/redirect/outbound?p=hmdb&param=HMDB0132825
http://nonlinear.com/redirect/outbound?p=hmdb&param=HMDB0132828
http://nonlinear.com/redirect/outbound?p=hmdb&param=HMDB0132831
http://nonlinear.com/redirect/outbound?p=hmdb&param=HMDB0132832
http://nonlinear.com/redirect/outbound?p=hmdb&param=HMDB0132833
http://nonlinear.com/redirect/outbound?p=hmdb&param=HMDB0132835
http://nonlinear.com/redirect/outbound?p=hmdb&param=HMDB0132836
http://nonlinear.com/redirect/outbound?p=hmdb&param=HMDB0132837
http://nonlinear.com/redirect/outbound?p=hmdb&param=HMDB0132838
http://nonlinear.com/redirect/outbound?p=hmdb&param=HMDB0132839
http://nonlinear.com/redirect/outbound?p=hmdb&param=HMDB0132840
http://nonlinear.com/redirect/outbound?p=hmdb&param=HMDB0132841
http://nonlinear.com/redirect/outbound?p=hmdb&param=HMDB0132842
http://nonlinear.com/redirect/outbound?p=hmdb&param=HMDB0132843
http://nonlinear.com/redirect/outbound?p=hmdb&param=HMDB0132844
http://nonlinear.com/redirect/outbound?p=hmdb&param=HMDB0132845
http://nonlinear.com/redirect/outbound?p=hmdb&param=HMDB0132846
http://nonlinear.com/redirect/outbound?p=hmdb&param=HMDB0132847
http://nonlinear.com/redirect/outbound?p=hmdb&param=HMDB0132848
http://nonlinear.com/redirect/outbound?p=hmdb&param=HMDB0031149
http://nonlinear.com/redirect/outbound?p=hmdb&param=HMDB0030627
http://nonlinear.com/redirect/outbound?p=hmdb&param=HMDB0034281
http://nonlinear.com/redirect/outbound?p=hmdb&param=HMDB0030627
http://nonlinear.com/redirect/outbound?p=hmdb&param=HMDB0034281
http://nonlinear.com/redirect/outbound?p=hmdb&param=HMDB0001337
http://nonlinear.com/redirect/outbound?p=hmdb&param=HMDB0005081
http://nonlinear.com/redirect/outbound?p=hmdb&param=HMDB0006027
http://nonlinear.com/redirect/outbound?p=hmdb&param=HMDB0010202
http://nonlinear.com/redirect/outbound?p=hmdb&param=HMDB0010205
http://nonlinear.com/redirect/outbound?p=hmdb&param=HMDB0010209
http://nonlinear.com/redirect/outbound?p=hmdb&param=HMDB0010210
http://nonlinear.com/redirect/outbound?p=hmdb&param=HMDB0010212
http://nonlinear.com/redirect/outbound?p=hmdb&param=HMDB0010217
http://nonlinear.com/redirect/outbound?p=hmdb&param=HMDB0012534
http://nonlinear.com/redirect/outbound?p=hmdb&param=HMDB0012611
http://nonlinear.com/redirect/outbound?p=hmdb&param=HMDB0013633
http://nonlinear.com/redirect/outbound?p=hmdb&param=HMDB0030101
http://nonlinear.com/redirect/outbound?p=hmdb&param=HMDB0035116
http://nonlinear.com/redirect/outbound?p=hmdb&param=HMDB0035311
http://nonlinear.com/redirect/outbound?p=hmdb&param=HMDB0036698
http://nonlinear.com/redirect/outbound?p=hmdb&param=HMDB0036706
http://nonlinear.com/redirect/outbound?p=hmdb&param=HMDB0036712
http://nonlinear.com/redirect/outbound?p=hmdb&param=HMDB0036713

http://nonlinear.com/redirect/outbound?p=hmdb&param=HMDB0036724
http://nonlinear.com/redirect/outbound?p=hmdb&param=HMDB0036752
http://nonlinear.com/redirect/outbound?p=hmdb&param=HMDB0036776
http://nonlinear.com/redirect/outbound?p=hmdb&param=HMDB0036806
http://nonlinear.com/redirect/outbound?p=hmdb&param=HMDB0036839
http://nonlinear.com/redirect/outbound?p=hmdb&param=HMDB0036930
http://nonlinear.com/redirect/outbound?p=hmdb&param=HMDB0037523
http://nonlinear.com/redirect/outbound?p=hmdb&param=HMDB0038521
http://nonlinear.com/redirect/outbound?p=hmdb&param=HMDB0039012
http://nonlinear.com/redirect/outbound?p=hmdb&param=HMDB0039716
http://nonlinear.com/redirect/outbound?p=hmdb&param=HMDB0041058
http://nonlinear.com/redirect/outbound?p=hmdb&param=HMDB0060053
http://nonlinear.com/redirect/outbound?p=hmdb&param=HMDB0061114
http://nonlinear.com/redirect/outbound?p=hmdb&param=HMDB0062222
http://nonlinear.com/redirect/outbound?p=hmdb&param=HMDB0062293
http://nonlinear.com/redirect/outbound?p=hmdb&param=HMDB0062296
http://nonlinear.com/redirect/outbound?p=hmdb&param=HMDB0062408
http://nonlinear.com/redirect/outbound?p=hmdb&param=HMDB0137419
http://nonlinear.com/redirect/outbound?p=hmdb&param=HMDB0000825
http://nonlinear.com/redirect/outbound?p=hmdb&param=HMDB0006569
http://nonlinear.com/redirect/outbound?p=hmdb&param=HMDB0129415
http://nonlinear.com/redirect/outbound?p=hmdb&param=HMDB0129416
http://nonlinear.com/redirect/outbound?p=hmdb&param=HMDB0129417
http://nonlinear.com/redirect/outbound?p=hmdb&param=HMDB0132767
http://nonlinear.com/redirect/outbound?p=hmdb&param=HMDB0132768
http://nonlinear.com/redirect/outbound?p=hmdb&param=HMDB0132770
http://nonlinear.com/redirect/outbound?p=hmdb&param=HMDB0132771
http://nonlinear.com/redirect/outbound?p=hmdb&param=HMDB0132772
http://nonlinear.com/redirect/outbound?p=hmdb&param=HMDB0132774
http://nonlinear.com/redirect/outbound?p=hmdb&param=HMDB0132775
http://nonlinear.com/redirect/outbound?p=hmdb&param=HMDB0132776
http://nonlinear.com/redirect/outbound?p=hmdb&param=HMDB0132777
http://nonlinear.com/redirect/outbound?p=hmdb&param=HMDB0132778
http://nonlinear.com/redirect/outbound?p=hmdb&param=HMDB0132779
http://nonlinear.com/redirect/outbound?p=hmdb&param=HMDB0132780
http://nonlinear.com/redirect/outbound?p=hmdb&param=HMDB0132782
http://nonlinear.com/redirect/outbound?p=hmdb&param=HMDB0132783
http://nonlinear.com/redirect/outbound?p=hmdb&param=HMDB0132784
http://nonlinear.com/redirect/outbound?p=hmdb&param=HMDB0132786
http://nonlinear.com/redirect/outbound?p=hmdb&param=HMDB0132787
http://nonlinear.com/redirect/outbound?p=hmdb&param=HMDB0132788
http://nonlinear.com/redirect/outbound?p=hmdb&param=HMDB0132789
http://nonlinear.com/redirect/outbound?p=hmdb&param=HMDB0132791
http://nonlinear.com/redirect/outbound?p=hmdb&param=HMDB0132792
http://nonlinear.com/redirect/outbound?p=hmdb&param=HMDB0132793
http://nonlinear.com/redirect/outbound?p=hmdb&param=HMDB0132811
http://nonlinear.com/redirect/outbound?p=hmdb&param=HMDB0132812
http://nonlinear.com/redirect/outbound?p=hmdb&param=HMDB0132813
http://nonlinear.com/redirect/outbound?p=hmdb&param=HMDB0132815
http://nonlinear.com/redirect/outbound?p=hmdb&param=HMDB0132816

http://nonlinear.com/redirect/outbound?p=hmdb&param=HMDB0132817
http://nonlinear.com/redirect/outbound?p=hmdb&param=HMDB0132819
http://nonlinear.com/redirect/outbound?p=hmdb&param=HMDB0132820
http://nonlinear.com/redirect/outbound?p=hmdb&param=HMDB0132821
http://nonlinear.com/redirect/outbound?p=hmdb&param=HMDB0132822
http://nonlinear.com/redirect/outbound?p=hmdb&param=HMDB0132823
http://nonlinear.com/redirect/outbound?p=hmdb&param=HMDB0132824
http://nonlinear.com/redirect/outbound?p=hmdb&param=HMDB0132825
http://nonlinear.com/redirect/outbound?p=hmdb&param=HMDB0132828
http://nonlinear.com/redirect/outbound?p=hmdb&param=HMDB0132831
http://nonlinear.com/redirect/outbound?p=hmdb&param=HMDB0132832
http://nonlinear.com/redirect/outbound?p=hmdb&param=HMDB0132833
http://nonlinear.com/redirect/outbound?p=hmdb&param=HMDB0132835
http://nonlinear.com/redirect/outbound?p=hmdb&param=HMDB0132836
http://nonlinear.com/redirect/outbound?p=hmdb&param=HMDB0132837
http://nonlinear.com/redirect/outbound?p=hmdb&param=HMDB0132838
http://nonlinear.com/redirect/outbound?p=hmdb&param=HMDB0132839
http://nonlinear.com/redirect/outbound?p=hmdb&param=HMDB0132840
http://nonlinear.com/redirect/outbound?p=hmdb&param=HMDB0132841
http://nonlinear.com/redirect/outbound?p=hmdb&param=HMDB0132842
http://nonlinear.com/redirect/outbound?p=hmdb&param=HMDB0132843
http://nonlinear.com/redirect/outbound?p=hmdb&param=HMDB0132844
http://nonlinear.com/redirect/outbound?p=hmdb&param=HMDB0132845
http://nonlinear.com/redirect/outbound?p=hmdb&param=HMDB0132846
http://nonlinear.com/redirect/outbound?p=hmdb&param=HMDB0132847
http://nonlinear.com/redirect/outbound?p=hmdb&param=HMDB0132848
http://nonlinear.com/redirect/outbound?p=hmdb&param=HMDB0129415
http://nonlinear.com/redirect/outbound?p=hmdb&param=HMDB0129416
http://nonlinear.com/redirect/outbound?p=hmdb&param=HMDB0129417
http://nonlinear.com/redirect/outbound?p=hmdb&param=HMDB0132767
http://nonlinear.com/redirect/outbound?p=hmdb&param=HMDB0132768
http://nonlinear.com/redirect/outbound?p=hmdb&param=HMDB0132770
http://nonlinear.com/redirect/outbound?p=hmdb&param=HMDB0132771
http://nonlinear.com/redirect/outbound?p=hmdb&param=HMDB0132772
http://nonlinear.com/redirect/outbound?p=hmdb&param=HMDB0132774
http://nonlinear.com/redirect/outbound?p=hmdb&param=HMDB0132775
http://nonlinear.com/redirect/outbound?p=hmdb&param=HMDB0132776
http://nonlinear.com/redirect/outbound?p=hmdb&param=HMDB0132777
http://nonlinear.com/redirect/outbound?p=hmdb&param=HMDB0132778
http://nonlinear.com/redirect/outbound?p=hmdb&param=HMDB0132779
http://nonlinear.com/redirect/outbound?p=hmdb&param=HMDB0132780
http://nonlinear.com/redirect/outbound?p=hmdb&param=HMDB0132782
http://nonlinear.com/redirect/outbound?p=hmdb&param=HMDB0132783
http://nonlinear.com/redirect/outbound?p=hmdb&param=HMDB0132784
http://nonlinear.com/redirect/outbound?p=hmdb&param=HMDB0132786
http://nonlinear.com/redirect/outbound?p=hmdb&param=HMDB0132787
http://nonlinear.com/redirect/outbound?p=hmdb&param=HMDB0132788
http://nonlinear.com/redirect/outbound?p=hmdb&param=HMDB0132789
http://nonlinear.com/redirect/outbound?p=hmdb&param=HMDB0132791
http://nonlinear.com/redirect/outbound?p=hmdb&param=HMDB0132792

http://nonlinear.com/redirect/outbound?p=hmdb&param=HMDB0132793
http://nonlinear.com/redirect/outbound?p=hmdb&param=HMDB0132811
http://nonlinear.com/redirect/outbound?p=hmdb&param=HMDB0132812
http://nonlinear.com/redirect/outbound?p=hmdb&param=HMDB0132813
http://nonlinear.com/redirect/outbound?p=hmdb&param=HMDB0132815
http://nonlinear.com/redirect/outbound?p=hmdb&param=HMDB0132816
http://nonlinear.com/redirect/outbound?p=hmdb&param=HMDB0132817
http://nonlinear.com/redirect/outbound?p=hmdb&param=HMDB0132819
http://nonlinear.com/redirect/outbound?p=hmdb&param=HMDB0132820
http://nonlinear.com/redirect/outbound?p=hmdb&param=HMDB0132821
http://nonlinear.com/redirect/outbound?p=hmdb&param=HMDB0132822
http://nonlinear.com/redirect/outbound?p=hmdb&param=HMDB0132823
http://nonlinear.com/redirect/outbound?p=hmdb&param=HMDB0132824
http://nonlinear.com/redirect/outbound?p=hmdb&param=HMDB0132825
http://nonlinear.com/redirect/outbound?p=hmdb&param=HMDB0132828
http://nonlinear.com/redirect/outbound?p=hmdb&param=HMDB0132831
http://nonlinear.com/redirect/outbound?p=hmdb&param=HMDB0132832
http://nonlinear.com/redirect/outbound?p=hmdb&param=HMDB0132833
http://nonlinear.com/redirect/outbound?p=hmdb&param=HMDB0132835
http://nonlinear.com/redirect/outbound?p=hmdb&param=HMDB0132836
http://nonlinear.com/redirect/outbound?p=hmdb&param=HMDB0132837
http://nonlinear.com/redirect/outbound?p=hmdb&param=HMDB0132838
http://nonlinear.com/redirect/outbound?p=hmdb&param=HMDB0132839
http://nonlinear.com/redirect/outbound?p=hmdb&param=HMDB0132840
http://nonlinear.com/redirect/outbound?p=hmdb&param=HMDB0132841
http://nonlinear.com/redirect/outbound?p=hmdb&param=HMDB0132842
http://nonlinear.com/redirect/outbound?p=hmdb&param=HMDB0132843
http://nonlinear.com/redirect/outbound?p=hmdb&param=HMDB0132844
http://nonlinear.com/redirect/outbound?p=hmdb&param=HMDB0132845
http://nonlinear.com/redirect/outbound?p=hmdb&param=HMDB0132846
http://nonlinear.com/redirect/outbound?p=hmdb&param=HMDB0132847
http://nonlinear.com/redirect/outbound?p=hmdb&param=HMDB0132848
http://nonlinear.com/redirect/outbound?p=hmdb&param=HMDB0014722
http://nonlinear.com/redirect/outbound?p=hmdb&param=HMDB0015036
http://nonlinear.com/redirect/outbound?p=hmdb&param=HMDB0014624
http://nonlinear.com/redirect/outbound?p=hmdb&param=HMDB0002916
http://nonlinear.com/redirect/outbound?p=hmdb&param=HMDB0006200
http://nonlinear.com/redirect/outbound?p=hmdb&param=HMDB0062694
http://nonlinear.com/redirect/outbound?p=hmdb&param=HMDB0000825
http://nonlinear.com/redirect/outbound?p=hmdb&param=HMDB0006569
http://nonlinear.com/redirect/outbound?p=hmdb&param=HMDB0129415
http://nonlinear.com/redirect/outbound?p=hmdb&param=HMDB0129416
http://nonlinear.com/redirect/outbound?p=hmdb&param=HMDB0129417
http://nonlinear.com/redirect/outbound?p=hmdb&param=HMDB0132767
http://nonlinear.com/redirect/outbound?p=hmdb&param=HMDB0132768
http://nonlinear.com/redirect/outbound?p=hmdb&param=HMDB0132770
http://nonlinear.com/redirect/outbound?p=hmdb&param=HMDB0132771
http://nonlinear.com/redirect/outbound?p=hmdb&param=HMDB0132772
http://nonlinear.com/redirect/outbound?p=hmdb&param=HMDB0132774
http://nonlinear.com/redirect/outbound?p=hmdb&param=HMDB0132775

http://nonlinear.com/redirect/outbound?p=hmdb&param=HMDB0132776
http://nonlinear.com/redirect/outbound?p=hmdb&param=HMDB0132777
http://nonlinear.com/redirect/outbound?p=hmdb&param=HMDB0132778
http://nonlinear.com/redirect/outbound?p=hmdb&param=HMDB0132779
http://nonlinear.com/redirect/outbound?p=hmdb&param=HMDB0132780
http://nonlinear.com/redirect/outbound?p=hmdb&param=HMDB0132782
http://nonlinear.com/redirect/outbound?p=hmdb&param=HMDB0132783
http://nonlinear.com/redirect/outbound?p=hmdb&param=HMDB0132784
http://nonlinear.com/redirect/outbound?p=hmdb&param=HMDB0132786
http://nonlinear.com/redirect/outbound?p=hmdb&param=HMDB0132787
http://nonlinear.com/redirect/outbound?p=hmdb&param=HMDB0132788
http://nonlinear.com/redirect/outbound?p=hmdb&param=HMDB0132789
http://nonlinear.com/redirect/outbound?p=hmdb&param=HMDB0132791
http://nonlinear.com/redirect/outbound?p=hmdb&param=HMDB0132792
http://nonlinear.com/redirect/outbound?p=hmdb&param=HMDB0132793
http://nonlinear.com/redirect/outbound?p=hmdb&param=HMDB0132811
http://nonlinear.com/redirect/outbound?p=hmdb&param=HMDB0132812
http://nonlinear.com/redirect/outbound?p=hmdb&param=HMDB0132813
http://nonlinear.com/redirect/outbound?p=hmdb&param=HMDB0132815
http://nonlinear.com/redirect/outbound?p=hmdb&param=HMDB0132816
http://nonlinear.com/redirect/outbound?p=hmdb&param=HMDB0132817
http://nonlinear.com/redirect/outbound?p=hmdb&param=HMDB0132819
http://nonlinear.com/redirect/outbound?p=hmdb&param=HMDB0132820
http://nonlinear.com/redirect/outbound?p=hmdb&param=HMDB0132821
http://nonlinear.com/redirect/outbound?p=hmdb&param=HMDB0132822
http://nonlinear.com/redirect/outbound?p=hmdb&param=HMDB0132823
http://nonlinear.com/redirect/outbound?p=hmdb&param=HMDB0132824
http://nonlinear.com/redirect/outbound?p=hmdb&param=HMDB0132825
http://nonlinear.com/redirect/outbound?p=hmdb&param=HMDB0132828
http://nonlinear.com/redirect/outbound?p=hmdb&param=HMDB0132831
http://nonlinear.com/redirect/outbound?p=hmdb&param=HMDB0132832
http://nonlinear.com/redirect/outbound?p=hmdb&param=HMDB0132833
http://nonlinear.com/redirect/outbound?p=hmdb&param=HMDB0132835
http://nonlinear.com/redirect/outbound?p=hmdb&param=HMDB0132836
http://nonlinear.com/redirect/outbound?p=hmdb&param=HMDB0132837
http://nonlinear.com/redirect/outbound?p=hmdb&param=HMDB0132838
http://nonlinear.com/redirect/outbound?p=hmdb&param=HMDB0132839
http://nonlinear.com/redirect/outbound?p=hmdb&param=HMDB0132840
http://nonlinear.com/redirect/outbound?p=hmdb&param=HMDB0132841
http://nonlinear.com/redirect/outbound?p=hmdb&param=HMDB0132842
http://nonlinear.com/redirect/outbound?p=hmdb&param=HMDB0132843
http://nonlinear.com/redirect/outbound?p=hmdb&param=HMDB0132844
http://nonlinear.com/redirect/outbound?p=hmdb&param=HMDB0132845
http://nonlinear.com/redirect/outbound?p=hmdb&param=HMDB0132846
http://nonlinear.com/redirect/outbound?p=hmdb&param=HMDB0132847
http://nonlinear.com/redirect/outbound?p=hmdb&param=HMDB0132848
http://nonlinear.com/redirect/outbound?p=hmdb&param=HMDB0129415
http://nonlinear.com/redirect/outbound?p=hmdb&param=HMDB0129416
http://nonlinear.com/redirect/outbound?p=hmdb&param=HMDB0129417
http://nonlinear.com/redirect/outbound?p=hmdb&param=HMDB0132767

http://nonlinear.com/redirect/outbound?p=hmdb&param=HMDB0132768
http://nonlinear.com/redirect/outbound?p=hmdb&param=HMDB0132770
http://nonlinear.com/redirect/outbound?p=hmdb&param=HMDB0132771
http://nonlinear.com/redirect/outbound?p=hmdb&param=HMDB0132772
http://nonlinear.com/redirect/outbound?p=hmdb&param=HMDB0132774
http://nonlinear.com/redirect/outbound?p=hmdb&param=HMDB0132775
http://nonlinear.com/redirect/outbound?p=hmdb&param=HMDB0132776
http://nonlinear.com/redirect/outbound?p=hmdb&param=HMDB0132777
http://nonlinear.com/redirect/outbound?p=hmdb&param=HMDB0132778
http://nonlinear.com/redirect/outbound?p=hmdb&param=HMDB0132779
http://nonlinear.com/redirect/outbound?p=hmdb&param=HMDB0132780
http://nonlinear.com/redirect/outbound?p=hmdb&param=HMDB0132782
http://nonlinear.com/redirect/outbound?p=hmdb&param=HMDB0132783
http://nonlinear.com/redirect/outbound?p=hmdb&param=HMDB0132784
http://nonlinear.com/redirect/outbound?p=hmdb&param=HMDB0132786
http://nonlinear.com/redirect/outbound?p=hmdb&param=HMDB0132787
http://nonlinear.com/redirect/outbound?p=hmdb&param=HMDB0132788
http://nonlinear.com/redirect/outbound?p=hmdb&param=HMDB0132789
http://nonlinear.com/redirect/outbound?p=hmdb&param=HMDB0132791
http://nonlinear.com/redirect/outbound?p=hmdb&param=HMDB0132792
http://nonlinear.com/redirect/outbound?p=hmdb&param=HMDB0132793
http://nonlinear.com/redirect/outbound?p=hmdb&param=HMDB0132811
http://nonlinear.com/redirect/outbound?p=hmdb&param=HMDB0132812
http://nonlinear.com/redirect/outbound?p=hmdb&param=HMDB0132813
http://nonlinear.com/redirect/outbound?p=hmdb&param=HMDB0132815
http://nonlinear.com/redirect/outbound?p=hmdb&param=HMDB0132816
http://nonlinear.com/redirect/outbound?p=hmdb&param=HMDB0132817
http://nonlinear.com/redirect/outbound?p=hmdb&param=HMDB0132819
http://nonlinear.com/redirect/outbound?p=hmdb&param=HMDB0132820
http://nonlinear.com/redirect/outbound?p=hmdb&param=HMDB0132821
http://nonlinear.com/redirect/outbound?p=hmdb&param=HMDB0132822
http://nonlinear.com/redirect/outbound?p=hmdb&param=HMDB0132823
http://nonlinear.com/redirect/outbound?p=hmdb&param=HMDB0132824
http://nonlinear.com/redirect/outbound?p=hmdb&param=HMDB0132825
http://nonlinear.com/redirect/outbound?p=hmdb&param=HMDB0132828
http://nonlinear.com/redirect/outbound?p=hmdb&param=HMDB0132831
http://nonlinear.com/redirect/outbound?p=hmdb&param=HMDB0132832
http://nonlinear.com/redirect/outbound?p=hmdb&param=HMDB0132833
http://nonlinear.com/redirect/outbound?p=hmdb&param=HMDB0132835
http://nonlinear.com/redirect/outbound?p=hmdb&param=HMDB0132836
http://nonlinear.com/redirect/outbound?p=hmdb&param=HMDB0132837
http://nonlinear.com/redirect/outbound?p=hmdb&param=HMDB0132838
http://nonlinear.com/redirect/outbound?p=hmdb&param=HMDB0132839
http://nonlinear.com/redirect/outbound?p=hmdb&param=HMDB0132840
http://nonlinear.com/redirect/outbound?p=hmdb&param=HMDB0132841
http://nonlinear.com/redirect/outbound?p=hmdb&param=HMDB0132842
http://nonlinear.com/redirect/outbound?p=hmdb&param=HMDB0132843
http://nonlinear.com/redirect/outbound?p=hmdb&param=HMDB0132844
http://nonlinear.com/redirect/outbound?p=hmdb&param=HMDB0132845
http://nonlinear.com/redirect/outbound?p=hmdb&param=HMDB0132846

http://nonlinear.com/redirect/outbound?p=hmdb&param=HMDB0132847
http://nonlinear.com/redirect/outbound?p=hmdb&param=HMDB0132848
http://nonlinear.com/redirect/outbound?p=hmdb&param=HMDB0014722
http://nonlinear.com/redirect/outbound?p=hmdb&param=HMDB0015036
http://nonlinear.com/redirect/outbound?p=hmdb&param=HMDB0014624

Description
Dide-O-methyl-4-O-alpha-D-glucopyranosylsimmondsin
Dide-O-methyl-4-O-alpha-D-glucopyranosylsimmondsin
Lacto-N-triaose
Lacto-N-triose I
N-Acetylgalactosaminyl lactose
Lacto-N-triaose
Lacto-N-triose I
N-Acetylgalactosaminyl lactose
6-{[6-(2-carboxyeth-1-en-1-yl)-4-methoxy-2H-1,3-benzodioxol-5-yl]oxy}-3,4,5-trihydroxyoxane-2-carboxylic acid
3,4,5-trihydroxy-6-{[3-(6-hydroxy-7-methoxy-2H-1,3-benzodioxol-5-yl)prop-2-enoyl]oxy}oxane-2-carboxylic acid
6-{[6-(2-carboxyeth-1-en-1-yl)-5-methoxy-2H-1,3-benzodioxol-4-yl]oxy}-3,4,5-trihydroxyoxane-2-carboxylic acid
3,4,5-trihydroxy-6-{[3-(7-hydroxy-6-methoxy-2H-1,3-benzodioxol-5-yl)prop-2-enoyl]oxy}oxane-2-carboxylic acid
6-{[6-(2-carboxyeth-1-en-1-yl)-4-methoxy-2H-1,3-benzodioxol-5-yl]oxy}-3,4,5-trihydroxyoxane-2-carboxylic acid
3,4,5-trihydroxy-6-{[3-(6-hydroxy-7-methoxy-2H-1,3-benzodioxol-5-yl)prop-2-enoyl]oxy}oxane-2-carboxylic acid
6-{[6-(2-carboxyeth-1-en-1-yl)-5-methoxy-2H-1,3-benzodioxol-4-yl]oxy}-3,4,5-trihydroxyoxane-2-carboxylic acid
3,4,5-trihydroxy-6-{[3-(7-hydroxy-6-methoxy-2H-1,3-benzodioxol-5-yl)prop-2-enoyl]oxy}oxane-2-carboxylic acid
SAICAR
SAICAR
Kuwanon J
Artonin C
3-(2,4-dihydroxyphenyl)-1-{3-[5-(2,4-dihydroxyphenyl)-6-(5-hydroxy-2,2-dimethyl-2H-chromene-6-carbonyl)-3-meth
Methantheline
Mepenzolate
Kuwanon J
Artonin C
3-(2,4-dihydroxyphenyl)-1-{3-[5-(2,4-dihydroxyphenyl)-6-(5-hydroxy-2,2-dimethyl-2H-chromene-6-carbonyl)-3-meth
Methantheline
Mepenzolate
3'-Sialyllactosamine
6'-Sialyllactosamine
3'-Sialyllactosamine
6'-Sialyllactosamine
Pentamidine
Pentamidine
Glycopyrrolate
Glycopyrrolate
Betaine
L-Valine
Vaporole
N-Methyl-a-aminoisobutyric acid
5-Aminopentanoic acid
Norvaline
Amyl Nitrite
Betaine
L-Valine
Vaporole
5-Methyltetrahydrofolic acid
Daunorubicin

Doxorubicinol
Doxorubicinol aglycone
LysoPE(0:0/22:6(4Z,7Z,10Z,13Z,16Z,19Z))
LysoPE(22:6(4Z,7Z,10Z,13Z,16Z,19Z)/0:0)
LysoPE(0:0/22:6(4Z,7Z,10Z,13Z,16Z,19Z))
LysoPE(22:6(4Z,7Z,10Z,13Z,16Z,19Z)/0:0)
6-{[6,15-dihydroxy-19,19-dimethyl-22-(3-methylbut-2-en-1-yl)-11-(2-methylprop-1-en-1-yl)-13-oxo-2,10,20-trioxape
6-{[7,15-dihydroxy-19,19-dimethyl-22-(3-methylbut-2-en-1-yl)-11-(2-methylprop-1-en-1-yl)-13-oxo-2,10,20-trioxape
6-{[6,7-dihydroxy-19,19-dimethyl-22-(3-methylbut-2-en-1-yl)-11-(2-methylprop-1-en-1-yl)-13-oxo-2,10,20-trioxaper
Dhurrin 6'-glucoside
6-{[6,15-dihydroxy-19,19-dimethyl-22-(3-methylbut-2-en-1-yl)-11-(2-methylprop-1-en-1-yl)-13-oxo-2,10,20-trioxape
6-{[7,15-dihydroxy-19,19-dimethyl-22-(3-methylbut-2-en-1-yl)-11-(2-methylprop-1-en-1-yl)-13-oxo-2,10,20-trioxape
6-{[6,7-dihydroxy-19,19-dimethyl-22-(3-methylbut-2-en-1-yl)-11-(2-methylprop-1-en-1-yl)-13-oxo-2,10,20-trioxaper
Dhurrin 6'-glucoside
2-amino-4-({1-[(carboxymethyl)-C-hydroxycarbonimidoyl]-2-{[1-hydroxy-1-(4-methoxyphenyl)-4-methyl-3-oxopenta
2-amino-4-({1-[(carboxymethyl)-C-hydroxycarbonimidoyl]-2-{[2-hydroxy-1-(4-methoxyphenyl)-4-methyl-3-oxopenty
2-amino-4-({1-[(carboxymethyl)-C-hydroxycarbonimidoyl]-2-{[1-hydroxy-1-(4-methoxyphenyl)-4-methyl-3-oxopenta
2-amino-4-({1-[(carboxymethyl)-C-hydroxycarbonimidoyl]-2-{[2-hydroxy-1-(4-methoxyphenyl)-4-methyl-3-oxopenty
Rosuvastatin
L-Citronellol glucoside
Menthyl pyrrolidone carboxylate
FADH
Bis(5-hydroxynoracronycine)
3'-Sialyllactosamine
6'-Sialyllactosamine
6-(4-{5,7-dihydroxy-4-oxo-6-[3,4,5-trihydroxy-6-(hydroxymethyl)oxan-2-yl]-8-(3,4,5-trihydroxyoxan-2-yl)-4H-chrome
6-(5-{5,7-dihydroxy-4-oxo-6-[3,4,5-trihydroxy-6-(hydroxymethyl)oxan-2-yl]-8-(3,4,5-trihydroxyoxan-2-yl)-4H-chrome
6-{[2-(3,4-dihydroxyphenyl)-7-hydroxy-4-oxo-6-[3,4,5-trihydroxy-6-(hydroxymethyl)oxan-2-yl]-8-(3,4,5-trihydroxyox
6-{[2-(3,4-dihydroxyphenyl)-5-hydroxy-4-oxo-6-[3,4,5-trihydroxy-6-(hydroxymethyl)oxan-2-yl]-8-(3,4,5-trihydroxyox
6-(4-{3-[(3-{[3,4-dihydroxy-4-(hydroxymethyl)oxolan-2-yl]oxy}-4,5-dihydroxy-6-(hydroxymethyl)oxan-2-yl)oxy]-5,7-d
6-({3-[(3-{[3,4-dihydroxy-4-(hydroxymethyl)oxolan-2-yl]oxy}-4,5-dihydroxy-6-(hydroxymethyl)oxan-2-yl)oxy]-7-hydro
6-(4-{5,7-dihydroxy-4-oxo-6-[3,4,5-trihydroxy-6-(hydroxymethyl)oxan-2-yl]-8-(3,4,5-trihydroxyoxan-2-yl)-4H-chrome
6-(5-{5,7-dihydroxy-4-oxo-6-[3,4,5-trihydroxy-6-(hydroxymethyl)oxan-2-yl]-8-(3,4,5-trihydroxyoxan-2-yl)-4H-chrome
6-{[2-(3,4-dihydroxyphenyl)-7-hydroxy-4-oxo-6-[3,4,5-trihydroxy-6-(hydroxymethyl)oxan-2-yl]-8-(3,4,5-trihydroxyox
6-{[2-(3,4-dihydroxyphenyl)-5-hydroxy-4-oxo-6-[3,4,5-trihydroxy-6-(hydroxymethyl)oxan-2-yl]-8-(3,4,5-trihydroxyox
6-(4-{3-[(3-{[3,4-dihydroxy-4-(hydroxymethyl)oxolan-2-yl]oxy}-4,5-dihydroxy-6-(hydroxymethyl)oxan-2-yl)oxy]-5,7-d
6-({3-[(3-{[3,4-dihydroxy-4-(hydroxymethyl)oxolan-2-yl]oxy}-4,5-dihydroxy-6-(hydroxymethyl)oxan-2-yl)oxy]-7-hydro
6-({3-[(3-{[3,4-dihydroxy-4-(hydroxymethyl)oxolan-2-yl]oxy}-4,5-dihydroxy-6-(hydroxymethyl)oxan-2-yl)oxy]-5-hydro
(7R)-7-(5-Carboxy-5-oxopentanoyl)aminocephalosporinate
6-{4-[3-(3,7-dimethylocta-2,6-dien-1-yl)-7-hydroxy-8-(3-methylbut-2-en-1-yl)-4-oxo-4H-chromen-2-yl]-3-hydroxyphe
6-{2-[3-(3,7-dimethylocta-2,6-dien-1-yl)-7-hydroxy-8-(3-methylbut-2-en-1-yl)-4-oxo-4H-chromen-2-yl]-5-hydroxyphe
6-{[2-(2,4-dihydroxyphenyl)-3-(3,7-dimethylocta-2,6-dien-1-yl)-8-(3-methylbut-2-en-1-yl)-4-oxo-4H-chromen-7-yl]ox
(7R)-7-(5-Carboxy-5-oxopentanoyl)aminocephalosporinate
6-{4-[3-(3,7-dimethylocta-2,6-dien-1-yl)-7-hydroxy-8-(3-methylbut-2-en-1-yl)-4-oxo-4H-chromen-2-yl]-3-hydroxyphe
6-{2-[3-(3,7-dimethylocta-2,6-dien-1-yl)-7-hydroxy-8-(3-methylbut-2-en-1-yl)-4-oxo-4H-chromen-2-yl]-5-hydroxyphe
6-{[2-(2,4-dihydroxyphenyl)-3-(3,7-dimethylocta-2,6-dien-1-yl)-8-(3-methylbut-2-en-1-yl)-4-oxo-4H-chromen-7-yl]ox
Alpha-Trisaccharide
Bis(5-hydroxynoracronycine)
3'-Prenylapigenin 7-[rhamnosyl-(1->6)-glucoside]
({6-[(2-{4-[(6-carboxy-3,4,5-trihydroxyoxan-2-yl)oxy]-3-hydroxyphenyl}-7-hydroxy-5-oxo-5H-chromen-3-yl)oxy]-3,4,5

({6-[(2-{3-[(6-carboxy-3,4,5-trihydroxyoxan-2-yl)oxy]-4-hydroxyphenyl}-7-hydroxy-5-oxo-5H-chromen-3-yl)oxy]-3,4,5
3-{[6-({[(2E)-3-{4-[(6-carboxy-3,4,5-trihydroxyoxan-2-yl)oxy]-3-hydroxyphenyl}prop-2-enoyl]oxy}methyl)-3,4,5-trihyd
(3-{3-[(6-carboxy-3,4,5-trihydroxyoxan-2-yl)oxy]-4-hydroxyphenyl}-1-hydroxyprop-2-en-1-ylidene)[(6-{[2-(3,4-dihydr
{[6-({7-[(6-carboxy-3,4,5-trihydroxyoxan-2-yl)oxy]-2-(3,4-dihydroxyphenyl)-5-oxo-5H-chromen-3-yl}oxy)-3,4,5-trihyd
5-[(6-carboxy-3,4,5-trihydroxyoxan-2-yl)oxy]-2-(3,4-dihydroxyphenyl)-3-{[6-({[3-(3,4-dihydroxyphenyl)prop-2-enoyl]
({6-[(2-{4-[(6-carboxy-3,4,5-trihydroxyoxan-2-yl)oxy]-3-hydroxyphenyl}-7-hydroxy-5-oxo-5H-chromen-3-yl)oxy]-3,4,5
({6-[(2-{3-[(6-carboxy-3,4,5-trihydroxyoxan-2-yl)oxy]-4-hydroxyphenyl}-7-hydroxy-5-oxo-5H-chromen-3-yl)oxy]-3,4,5
3-{[6-({[(2E)-3-{4-[(6-carboxy-3,4,5-trihydroxyoxan-2-yl)oxy]-3-hydroxyphenyl}prop-2-enoyl]oxy}methyl)-3,4,5-trihyd
(3-{3-[(6-carboxy-3,4,5-trihydroxyoxan-2-yl)oxy]-4-hydroxyphenyl}-1-hydroxyprop-2-en-1-ylidene)[(6-{[2-(3,4-dihydr
{[6-({7-[(6-carboxy-3,4,5-trihydroxyoxan-2-yl)oxy]-2-(3,4-dihydroxyphenyl)-5-oxo-5H-chromen-3-yl}oxy)-3,4,5-trihyd
5-[(6-carboxy-3,4,5-trihydroxyoxan-2-yl)oxy]-2-(3,4-dihydroxyphenyl)-3-{[6-({[3-(3,4-dihydroxyphenyl)prop-2-enoyl]
3'-Prenylapigenin 7-[rhamnosyl-(1->6)-glucoside]
Fluorometholone
Desoximetasone
Polyvidone
3'-Prenylapigenin 7-[rhamnosyl-(1->6)-glucoside]
(-)-Epigallocatechin 3-gallate 7-glucoside 4''-glucuronide
10-Formyltetrahydrofolate
Folinic acid
Pteroyl-D-glutamic acid
(6S)-5-formyltetrahydrofolate(2-)
Quercetin 3-(3'',6''-di-p-coumarylglucoside)
2-amino-4-({1-[(carboxymethyl)-C-hydroxycarbonimidoyl]-2-{[1-(4,5-dihydroxy-2,3-dimethoxyphenyl)-3-oxopropyl]s
4-Hydroxy-17beta-estradiol-2-S-glutathione
Perindoprilat
Neoacrimarine F
Neoacrimarine I
Dioxinoacrimarine A
Dioxinoacrimarine A
Acrimarine I
L-Aspartic acid
D-Aspartic acid
Iminodiacetic acid
1-deoxy-1-(N6-lysino)-D-fructose
N-acetyl-seryl-aspartate
Acrimarine I
Pranlukast
Indoleacetic acid
5-Hydroxyindoleacetaldehyde
Hydroxymethyl indol-3-yl ketone
1-Methoxy-1H-indole-3-carboxaldehyde
Pranlukast
Indoleacetic acid
5-Hydroxyindoleacetaldehyde
Hydroxymethyl indol-3-yl ketone
1-Methoxy-1H-indole-3-carboxaldehyde
4-Demethylsimmondsin 2'-(E)-ferulate
3-Demethylsimmondsin 2'-(Z)-ferulate
Kuwanon J
Artonin C

3-(2,4-dihydroxyphenyl)-1-{3-[5-(2,4-dihydroxyphenyl)-6-(5-hydroxy-2,2-dimethyl-2H-chromene-6-carbonyl)-3-meth
2-amino-4-({1-[(carboxymethyl)-C-hydroxycarbonimidoyl]-2-{[1,3-dihydroxy-1-(2-hydroxyphenyl)propan-2-yl]sulfany
4-Demethylsimmondsin 2'-(E)-ferulate
3-Demethylsimmondsin 2'-(Z)-ferulate
Kuwanon J
Artonin C
3-(2,4-dihydroxyphenyl)-1-{3-[5-(2,4-dihydroxyphenyl)-6-(5-hydroxy-2,2-dimethyl-2H-chromene-6-carbonyl)-3-meth
2-amino-4-({1-[(carboxymethyl)-C-hydroxycarbonimidoyl]-2-{[1,3-dihydroxy-1-(2-hydroxyphenyl)propan-2-yl]sulfany
5-Hydroxy-7,3',4'-trimethoxy-8-methylisoflavone 5-neohesperidoside
Egonol gentiobioside
6-[1-(3,4-dimethoxyphenyl)-2-{[2-(3,4-dimethoxyphenyl)ethyl]-C-hydroxycarbonimidoyl}ethoxy]-3,4,5-trihydroxyoxa
6-[1-(3,4-dimethoxyphenyl)-2-{[3-(3,4-dimethoxyphenyl)-1-hydroxypropylidene]amino}ethoxy]-3,4,5-trihydroxyoxan
6-[2-(3,4-dimethoxyphenyl)-1-{[2-(3,4-dimethoxyphenyl)ethyl]-C-hydroxycarbonimidoyl}ethoxy]-3,4,5-trihydroxyoxa
(E,E)-4,4''-Bi(N-4-hydroxycinnamoylserotonin)
1-nitrosonaphthalene
5-Hydroxy-7,3',4'-trimethoxy-8-methylisoflavone 5-neohesperidoside
Egonol gentiobioside
6-[1-(3,4-dimethoxyphenyl)-2-{[2-(3,4-dimethoxyphenyl)ethyl]-C-hydroxycarbonimidoyl}ethoxy]-3,4,5-trihydroxyoxa
6-[1-(3,4-dimethoxyphenyl)-2-{[3-(3,4-dimethoxyphenyl)-1-hydroxypropylidene]amino}ethoxy]-3,4,5-trihydroxyoxan
6-[2-(3,4-dimethoxyphenyl)-1-{[2-(3,4-dimethoxyphenyl)ethyl]-C-hydroxycarbonimidoyl}ethoxy]-3,4,5-trihydroxyoxa
(E,E)-4,4''-Bi(N-4-hydroxycinnamoylserotonin)
1-nitrosonaphthalene
3,4,5-trihydroxy-6-{3-hydroxy-4-[5-hydroxy-3-(3-hydroxy-3-methylbutyl)-8,8-dimethyl-4-oxo-4H,8H-pyrano[2,3-f]chr
3,4,5-trihydroxy-6-{5-hydroxy-2-[5-hydroxy-3-(3-hydroxy-3-methylbutyl)-8,8-dimethyl-4-oxo-4H,8H-pyrano[2,3-f]chr
6-{[2-(2,4-dihydroxyphenyl)-3-(3-hydroxy-3-methylbutyl)-8,8-dimethyl-4-oxo-4H,8H-pyrano[2,3-f]chromen-5-yl]oxy}
3,4,5-trihydroxy-6-({5-hydroxy-2-[5-hydroxy-9-(hydroxymethyl)-13,13-dimethyl-8-oxatetracyclo[7.4.1.0Â²,â�0Â¹Â²,Â
3,4,5-trihydroxy-6-({7-hydroxy-2-[5-hydroxy-9-(hydroxymethyl)-13,13-dimethyl-8-oxatetracyclo[7.4.1.0Â²,â�0Â¹Â²,Â
6-{[4-(5,7-dihydroxy-4-oxo-3,4-dihydro-2H-1-benzopyran-2-yl)-13-(hydroxymethyl)-9,13-dimethyl-8-oxatetracyclo[7
3,4,5-trihydroxy-6-({5-hydroxy-2-[5-hydroxy-13-(hydroxymethyl)-9,13-dimethyl-8-oxatetracyclo[7.4.1.0Â²,â�0Â¹Â²,Â
3,4,5-trihydroxy-6-({7-hydroxy-2-[5-hydroxy-13-(hydroxymethyl)-9,13-dimethyl-8-oxatetracyclo[7.4.1.0Â²,â�0Â¹Â²,Â
6-[(5,7-dihydroxy-2-{5-hydroxy-9,13,13-trimethyl-8-oxatetracyclo[7.4.1.0Â²,â�0Â¹Â²,Â¹â�]tetradeca-2(7),3,5-trien-4-
3,4,5-trihydroxy-6-{[9,13,13-trimethyl-4-(5,6,7-trihydroxy-4-oxo-3,4-dihydro-2H-1-benzopyran-2-yl)-8-oxatetracyclo
6-[(5,6-dihydroxy-2-{5-hydroxy-9,13,13-trimethyl-8-oxatetracyclo[7.4.1.0Â²,â�0Â¹Â²,Â¹â�]tetradeca-2(7),3,5-trien-4-
6-[(6,7-dihydroxy-2-{5-hydroxy-9,13,13-trimethyl-8-oxatetracyclo[7.4.1.0Â²,â�0Â¹Â²,Â¹â�]tetradeca-2(7),3,5-trien-4-
6-{[4-(5,7-dihydroxy-4-oxo-3,4-dihydro-2H-1-benzopyran-2-yl)-10-hydroxy-9,13,13-trimethyl-8-oxatetracyclo[7.4.1.0
6-[(2-{5,10-dihydroxy-9,13,13-trimethyl-8-oxatetracyclo[7.4.1.0Â²,â�0Â¹Â²,Â¹â�]tetradeca-2(7),3,5-trien-4-yl}-5-hydr
6-[(2-{5,10-dihydroxy-9,13,13-trimethyl-8-oxatetracyclo[7.4.1.0Â²,â�0Â¹Â²,Â¹â�]tetradeca-2(7),3,5-trien-4-yl}-7-hydr
6-{[4-(5,7-dihydroxy-4-oxo-3,4-dihydro-2H-1-benzopyran-2-yl)-11-hydroxy-9,13,13-trimethyl-8-oxatetracyclo[7.4.1.0
6-[(2-{5,11-dihydroxy-9,13,13-trimethyl-8-oxatetracyclo[7.4.1.0Â²,â�0Â¹Â²,Â¹â�]tetradeca-2(7),3,5-trien-4-yl}-5-hydr
6-[(2-{5,11-dihydroxy-9,13,13-trimethyl-8-oxatetracyclo[7.4.1.0Â²,â�0Â¹Â²,Â¹â�]tetradeca-2(7),3,5-trien-4-yl}-7-hydr
6-{[4-(5,7-dihydroxy-4-oxo-3,4-dihydro-2H-1-benzopyran-2-yl)-5-hydroxy-9,13,13-trimethyl-8-oxatetracyclo[7.4.1.0Â
6-{[4-(5,7-dihydroxy-4-oxo-3,4-dihydro-2H-1-benzopyran-2-yl)-6-hydroxy-9,13,13-trimethyl-8-oxatetracyclo[7.4.1.0Â
6-[(2-{5,6-dihydroxy-9,13,13-trimethyl-8-oxatetracyclo[7.4.1.0Â²,â�0Â¹Â²,Â¹â�]tetradeca-2(7),3,5-trien-4-yl}-5-hydro
6-[(2-{5,6-dihydroxy-9,13,13-trimethyl-8-oxatetracyclo[7.4.1.0Â²,â�0Â¹Â²,Â¹â�]tetradeca-2(7),3,5-trien-4-yl}-7-hydro
6-{[4-(5,7-dihydroxy-4-oxo-3,4-dihydro-2H-1-benzopyran-2-yl)-3-hydroxy-9,13,13-trimethyl-8-oxatetracyclo[7.4.1.0Â
6-[(2-{3,5-dihydroxy-9,13,13-trimethyl-8-oxatetracyclo[7.4.1.0Â²,â�0Â¹Â²,Â¹â�]tetradeca-2(7),3,5-trien-4-yl}-5-hydro
6-[(2-{3,5-dihydroxy-9,13,13-trimethyl-8-oxatetracyclo[7.4.1.0Â²,â�0Â¹Â²,Â¹â�]tetradeca-2(7),3,5-trien-4-yl}-7-hydro
6-{[6-(5,7-dihydroxy-4-oxo-3,4-dihydro-2H-1-benzopyran-2-yl)-2-(hydroxymethyl)-2-(4-methylpent-3-en-1-yl)-2H-ch
3,4,5-trihydroxy-6-({5-hydroxy-2-[7-hydroxy-2-(hydroxymethyl)-2-(4-methylpent-3-en-1-yl)-2H-chromen-6-yl]-4-oxo
3,4,5-trihydroxy-6-({7-hydroxy-2-[7-hydroxy-2-(hydroxymethyl)-2-(4-methylpent-3-en-1-yl)-2H-chromen-6-yl]-4-oxo

6-{[6-(5,7-dihydroxy-4-oxo-3,4-dihydro-2H-1-benzopyran-2-yl)-2-(1-hydroxy-4-methylpent-3-en-1-yl)-2-methyl-2H-c
3,4,5-trihydroxy-6-({5-hydroxy-2-[7-hydroxy-2-(1-hydroxy-4-methylpent-3-en-1-yl)-2-methyl-2H-chromen-6-yl]-4-ox
3,4,5-trihydroxy-6-({7-hydroxy-2-[7-hydroxy-2-(1-hydroxy-4-methylpent-3-en-1-yl)-2-methyl-2H-chromen-6-yl]-4-ox
6-({5,7-dihydroxy-2-[7-hydroxy-2-methyl-2-(4-methylpent-3-en-1-yl)-2H-chromen-6-yl]-4-oxo-3,4-dihydro-2H-1-ben
3,4,5-trihydroxy-6-{[2-methyl-2-(4-methylpent-3-en-1-yl)-6-(5,6,7-trihydroxy-4-oxo-3,4-dihydro-2H-1-benzopyran-2-
6-({5,6-dihydroxy-2-[7-hydroxy-2-methyl-2-(4-methylpent-3-en-1-yl)-2H-chromen-6-yl]-4-oxo-3,4-dihydro-2H-1-ben
6-({6,7-dihydroxy-2-[7-hydroxy-2-methyl-2-(4-methylpent-3-en-1-yl)-2H-chromen-6-yl]-4-oxo-3,4-dihydro-2H-1-ben
6-{[6-(5,7-dihydroxy-4-oxo-3,4-dihydro-2H-1-benzopyran-2-yl)-2-(5-hydroxy-4-methylpent-3-en-1-yl)-2-methyl-2H-c
3,4,5-trihydroxy-6-({5-hydroxy-2-[7-hydroxy-2-(5-hydroxy-4-methylpent-3-en-1-yl)-2-methyl-2H-chromen-6-yl]-4-ox
3,4,5-trihydroxy-6-({7-hydroxy-2-[7-hydroxy-2-(5-hydroxy-4-methylpent-3-en-1-yl)-2-methyl-2H-chromen-6-yl]-4-ox
3,4,5-trihydroxy-6-[(5-hydroxy-2-{7-hydroxy-2-[(3E)-5-hydroxy-4-methylpent-3-en-1-yl]-2-methyl-2H-chromen-6-yl}-
6-{[6-(5,7-dihydroxy-4-oxo-3,4-dihydro-2H-1-benzopyran-2-yl)-2-(2-hydroxy-4-methylpent-3-en-1-yl)-2-methyl-2H-c
3,4,5-trihydroxy-6-({5-hydroxy-2-[7-hydroxy-2-(2-hydroxy-4-methylpent-3-en-1-yl)-2-methyl-2H-chromen-6-yl]-4-ox
3,4,5-trihydroxy-6-({7-hydroxy-2-[7-hydroxy-2-(2-hydroxy-4-methylpent-3-en-1-yl)-2-methyl-2H-chromen-6-yl]-4-ox
6-{[6-(5,7-dihydroxy-4-oxo-3,4-dihydro-2H-1-benzopyran-2-yl)-2-[2-(3,3-dimethyloxiran-2-yl)ethyl]-2-methyl-2H-chr
6-[(2-{2-[2-(3,3-dimethyloxiran-2-yl)ethyl]-7-hydroxy-2-methyl-2H-chromen-6-yl}-5-hydroxy-4-oxo-3,4-dihydro-2H-1
6-[(2-{2-[2-(3,3-dimethyloxiran-2-yl)ethyl]-7-hydroxy-2-methyl-2H-chromen-6-yl}-7-hydroxy-4-oxo-3,4-dihydro-2H-1
6-{[10-(5,7-dihydroxy-4-oxo-3,4-dihydro-2H-1-benzopyran-2-yl)-5-methyl-5-(4-methylpent-3-en-1-yl)-3,6-dioxatricyc
3,4,5-trihydroxy-6-({5-hydroxy-2-[9-hydroxy-5-methyl-5-(4-methylpent-3-en-1-yl)-3,6-dioxatricyclo[5.4.0.0Â²,â�und
3,4,5-trihydroxy-6-({7-hydroxy-2-[9-hydroxy-5-methyl-5-(4-methylpent-3-en-1-yl)-3,6-dioxatricyclo[5.4.0.0Â²,â�und
6-{[6-(5,7-dihydroxy-4-oxo-3,4-dihydro-2H-1-benzopyran-2-yl)-7-hydroxy-2-methyl-2-(4-methylpent-3-en-1-yl)-2H-c
6-{[6-(5,7-dihydroxy-4-oxo-3,4-dihydro-2H-1-benzopyran-2-yl)-8-hydroxy-2-methyl-2-(4-methylpent-3-en-1-yl)-2H-c
6-({2-[7,8-dihydroxy-2-methyl-2-(4-methylpent-3-en-1-yl)-2H-chromen-6-yl]-5-hydroxy-4-oxo-3,4-dihydro-2H-1-ben
6-({2-[7,8-dihydroxy-2-methyl-2-(4-methylpent-3-en-1-yl)-2H-chromen-6-yl]-7-hydroxy-4-oxo-3,4-dihydro-2H-1-ben
6-{[6-(5,7-dihydroxy-4-oxo-3,4-dihydro-2H-1-benzopyran-2-yl)-7-hydroxy-2-methyl-2-(4-methylpent-3-en-1-yl)-2H-c
6-{[6-(5,7-dihydroxy-4-oxo-3,4-dihydro-2H-1-benzopyran-2-yl)-5-hydroxy-2-methyl-2-(4-methylpent-3-en-1-yl)-2H-c
6-({2-[5,7-dihydroxy-2-methyl-2-(4-methylpent-3-en-1-yl)-2H-chromen-6-yl]-5-hydroxy-4-oxo-3,4-dihydro-2H-1-ben
6-({2-[5,7-dihydroxy-2-methyl-2-(4-methylpent-3-en-1-yl)-2H-chromen-6-yl]-7-hydroxy-4-oxo-3,4-dihydro-2H-1-ben
Phaeophorbide b
5-Hydroxy-7,3',4'-trimethoxy-8-methylisoflavone 5-neohesperidoside
Egonol gentiobioside
5-Hydroxy-7,3',4'-trimethoxy-8-methylisoflavone 5-neohesperidoside
Egonol gentiobioside
Leukotriene A4
5-HEPE
Oxymesterone
12-HEPE
14,15-EpETE
15-HEPE
15-KETE
17,18-EpETE
5-KETE
11R-HEPE
18R-HEPE
12-KETE
Methyl [8]-Shogaol
(ent-7alpha)-7-Hydroxy-8(14),15-pimaradien-19-oic acid
Galanal A
Ineketone
Steviol

Yucalexin B20
Yucalexin B22
ent-17-Hydroxy-15-kauren-19-oic acid
Yucalexin P17
Yucalexin A19
Aframodial
(ent-15beta)-15-Hydroxy-19-trachylobanoic acid
Grandiflorolic acid
Galanolactone
2-Hydroxy-6-(8-tridecenyl)benzoic acid
(ent-15beta)-15,19-Dihydroxy-7-trachylobanone
12alpha-12-Hydroxy-7,13-abietadien-18-oic acid
Phytocassane C
9-HEPE
Atractyligenin
18R-hydroxy-5Z,8Z,11Z,14Z,16E-eicosapentaenoic acid
15R-hydroxy-5Z,8Z,11Z,13E,17Z-eicosapentaenoic acid
15S-hydroxy-5Z,8Z,11Z,13E,17Z-eicosapentaenoic acid
5S-hydroxy-6E,8Z,11Z,14Z,17Z-eicosapentaenoic acid
(4E)-1-(3,4-dihydroxyphenyl)tetradec-4-en-3-one
3,4,5-trihydroxy-6-{3-hydroxy-4-[5-hydroxy-3-(3-hydroxy-3-methylbutyl)-8,8-dimethyl-4-oxo-4H,8H-pyrano[2,3-f]chr
3,4,5-trihydroxy-6-{5-hydroxy-2-[5-hydroxy-3-(3-hydroxy-3-methylbutyl)-8,8-dimethyl-4-oxo-4H,8H-pyrano[2,3-f]chr
6-{[2-(2,4-dihydroxyphenyl)-3-(3-hydroxy-3-methylbutyl)-8,8-dimethyl-4-oxo-4H,8H-pyrano[2,3-f]chromen-5-yl]oxy}
3,4,5-trihydroxy-6-({5-hydroxy-2-[5-hydroxy-9-(hydroxymethyl)-13,13-dimethyl-8-oxatetracyclo[7.4.1.0Â²,â�0Â¹Â²,Â
3,4,5-trihydroxy-6-({7-hydroxy-2-[5-hydroxy-9-(hydroxymethyl)-13,13-dimethyl-8-oxatetracyclo[7.4.1.0Â²,â�0Â¹Â²,Â
6-{[4-(5,7-dihydroxy-4-oxo-3,4-dihydro-2H-1-benzopyran-2-yl)-13-(hydroxymethyl)-9,13-dimethyl-8-oxatetracyclo[7
3,4,5-trihydroxy-6-({5-hydroxy-2-[5-hydroxy-13-(hydroxymethyl)-9,13-dimethyl-8-oxatetracyclo[7.4.1.0Â²,â�0Â¹Â²,Â
3,4,5-trihydroxy-6-({7-hydroxy-2-[5-hydroxy-13-(hydroxymethyl)-9,13-dimethyl-8-oxatetracyclo[7.4.1.0Â²,â�0Â¹Â²,Â
6-[(5,7-dihydroxy-2-{5-hydroxy-9,13,13-trimethyl-8-oxatetracyclo[7.4.1.0Â²,â�0Â¹Â²,Â¹â�tetradeca-2(7),3,5-trien-4-
3,4,5-trihydroxy-6-{[9,13,13-trimethyl-4-(5,6,7-trihydroxy-4-oxo-3,4-dihydro-2H-1-benzopyran-2-yl)-8-oxatetracyclo
6-[(5,6-dihydroxy-2-{5-hydroxy-9,13,13-trimethyl-8-oxatetracyclo[7.4.1.0Â²,â�0Â¹Â²,Â¹â�tetradeca-2(7),3,5-trien-4-
6-[(6,7-dihydroxy-2-{5-hydroxy-9,13,13-trimethyl-8-oxatetracyclo[7.4.1.0Â²,â�0Â¹Â²,Â¹â�tetradeca-2(7),3,5-trien-4-
6-{[4-(5,7-dihydroxy-4-oxo-3,4-dihydro-2H-1-benzopyran-2-yl)-10-hydroxy-9,13,13-trimethyl-8-oxatetracyclo[7.4.1.0
6-[(2-{5,10-dihydroxy-9,13,13-trimethyl-8-oxatetracyclo[7.4.1.0Â²,â�0Â¹Â²,Â¹â�tetradeca-2(7),3,5-trien-4-yl}-5-hydr
6-[(2-{5,10-dihydroxy-9,13,13-trimethyl-8-oxatetracyclo[7.4.1.0Â²,â�0Â¹Â²,Â¹â�tetradeca-2(7),3,5-trien-4-yl}-7-hydr
6-{[4-(5,7-dihydroxy-4-oxo-3,4-dihydro-2H-1-benzopyran-2-yl)-11-hydroxy-9,13,13-trimethyl-8-oxatetracyclo[7.4.1.0
6-[(2-{5,11-dihydroxy-9,13,13-trimethyl-8-oxatetracyclo[7.4.1.0Â²,â�0Â¹Â²,Â¹â�tetradeca-2(7),3,5-trien-4-yl}-5-hydr
6-[(2-{5,11-dihydroxy-9,13,13-trimethyl-8-oxatetracyclo[7.4.1.0Â²,â�0Â¹Â²,Â¹â�tetradeca-2(7),3,5-trien-4-yl}-7-hydr
6-{[4-(5,7-dihydroxy-4-oxo-3,4-dihydro-2H-1-benzopyran-2-yl)-5-hydroxy-9,13,13-trimethyl-8-oxatetracyclo[7.4.1.0
6-{[4-(5,7-dihydroxy-4-oxo-3,4-dihydro-2H-1-benzopyran-2-yl)-6-hydroxy-9,13,13-trimethyl-8-oxatetracyclo[7.4.1.0
6-[(2-{5,6-dihydroxy-9,13,13-trimethyl-8-oxatetracyclo[7.4.1.0Â²,â�0Â¹Â²,Â¹â�tetradeca-2(7),3,5-trien-4-yl}-5-hydro
6-[(2-{5,6-dihydroxy-9,13,13-trimethyl-8-oxatetracyclo[7.4.1.0Â²,â�0Â¹Â²,Â¹â�tetradeca-2(7),3,5-trien-4-yl}-7-hydro
6-{[4-(5,7-dihydroxy-4-oxo-3,4-dihydro-2H-1-benzopyran-2-yl)-3-hydroxy-9,13,13-trimethyl-8-oxatetracyclo[7.4.1.0
6-[(2-{3,5-dihydroxy-9,13,13-trimethyl-8-oxatetracyclo[7.4.1.0Â²,â�0Â¹Â²,Â¹â�tetradeca-2(7),3,5-trien-4-yl}-5-hydro
6-[(2-{3,5-dihydroxy-9,13,13-trimethyl-8-oxatetracyclo[7.4.1.0Â²,â�0Â¹Â²,Â¹â�tetradeca-2(7),3,5-trien-4-yl}-7-hydro
6-{[6-(5,7-dihydroxy-4-oxo-3,4-dihydro-2H-1-benzopyran-2-yl)-2-(hydroxymethyl)-2-(4-methylpent-3-en-1-yl)-2H-ch
3,4,5-trihydroxy-6-({5-hydroxy-2-[7-hydroxy-2-(hydroxymethyl)-2-(4-methylpent-3-en-1-yl)-2H-chromen-6-yl]-4-oxo
3,4,5-trihydroxy-6-({7-hydroxy-2-[7-hydroxy-2-(hydroxymethyl)-2-(4-methylpent-3-en-1-yl)-2H-chromen-6-yl]-4-oxo
6-{[6-(5,7-dihydroxy-4-oxo-3,4-dihydro-2H-1-benzopyran-2-yl)-2-(1-hydroxy-4-methylpent-3-en-1-yl)-2-methyl-2H-c
3,4,5-trihydroxy-6-({5-hydroxy-2-[7-hydroxy-2-(1-hydroxy-4-methylpent-3-en-1-yl)-2-methyl-2H-chromen-6-yl]-4-ox

3,4,5-trihydroxy-6-({7-hydroxy-2-[7-hydroxy-2-(1-hydroxy-4-methylpent-3-en-1-yl)-2-methyl-2H-chromen-6-yl]-4-ox
6-({5,7-dihydroxy-2-[7-hydroxy-2-methyl-2-(4-methylpent-3-en-1-yl)-2H-chromen-6-yl]-4-oxo-3,4-dihydro-2H-1-ben
3,4,5-trihydroxy-6-{[2-methyl-2-(4-methylpent-3-en-1-yl)-6-(5,6,7-trihydroxy-4-oxo-3,4-dihydro-2H-1-benzopyran-2-
6-({5,6-dihydroxy-2-[7-hydroxy-2-methyl-2-(4-methylpent-3-en-1-yl)-2H-chromen-6-yl]-4-oxo-3,4-dihydro-2H-1-ben
6-({6,7-dihydroxy-2-[7-hydroxy-2-methyl-2-(4-methylpent-3-en-1-yl)-2H-chromen-6-yl]-4-oxo-3,4-dihydro-2H-1-ben
6-{[6-(5,7-dihydroxy-4-oxo-3,4-dihydro-2H-1-benzopyran-2-yl)-2-(5-hydroxy-4-methylpent-3-en-1-yl)-2-methyl-2H-c
3,4,5-trihydroxy-6-({5-hydroxy-2-[7-hydroxy-2-(5-hydroxy-4-methylpent-3-en-1-yl)-2-methyl-2H-chromen-6-yl]-4-ox
3,4,5-trihydroxy-6-({7-hydroxy-2-[7-hydroxy-2-(5-hydroxy-4-methylpent-3-en-1-yl)-2-methyl-2H-chromen-6-yl]-4-ox
3,4,5-trihydroxy-6-[(5-hydroxy-2-{7-hydroxy-2-[(3E)-5-hydroxy-4-methylpent-3-en-1-yl]-2-methyl-2H-chromen-6-yl}-
6-{[6-(5,7-dihydroxy-4-oxo-3,4-dihydro-2H-1-benzopyran-2-yl)-2-(2-hydroxy-4-methylpent-3-en-1-yl)-2-methyl-2H-c
3,4,5-trihydroxy-6-({5-hydroxy-2-[7-hydroxy-2-(2-hydroxy-4-methylpent-3-en-1-yl)-2-methyl-2H-chromen-6-yl]-4-ox
3,4,5-trihydroxy-6-({7-hydroxy-2-[7-hydroxy-2-(2-hydroxy-4-methylpent-3-en-1-yl)-2-methyl-2H-chromen-6-yl]-4-ox
6-{[6-(5,7-dihydroxy-4-oxo-3,4-dihydro-2H-1-benzopyran-2-yl)-2-[2-(3,3-dimethyloxiran-2-yl)ethyl]-2-methyl-2H-chr
6-[(2-{2-[2-(3,3-dimethyloxiran-2-yl)ethyl]-7-hydroxy-2-methyl-2H-chromen-6-yl}-5-hydroxy-4-oxo-3,4-dihydro-2H-1
6-[(2-{2-[2-(3,3-dimethyloxiran-2-yl)ethyl]-7-hydroxy-2-methyl-2H-chromen-6-yl}-7-hydroxy-4-oxo-3,4-dihydro-2H-1
6-{[10-(5,7-dihydroxy-4-oxo-3,4-dihydro-2H-1-benzopyran-2-yl)-5-methyl-5-(4-methylpent-3-en-1-yl)-3,6-dioxatricyc
3,4,5-trihydroxy-6-({5-hydroxy-2-[9-hydroxy-5-methyl-5-(4-methylpent-3-en-1-yl)-3,6-dioxatricyclo[5.4.0.0Â²,â�und
3,4,5-trihydroxy-6-({7-hydroxy-2-[9-hydroxy-5-methyl-5-(4-methylpent-3-en-1-yl)-3,6-dioxatricyclo[5.4.0.0Â²,â�und
6-{[6-(5,7-dihydroxy-4-oxo-3,4-dihydro-2H-1-benzopyran-2-yl)-7-hydroxy-2-methyl-2-(4-methylpent-3-en-1-yl)-2H-c
6-{[6-(5,7-dihydroxy-4-oxo-3,4-dihydro-2H-1-benzopyran-2-yl)-8-hydroxy-2-methyl-2-(4-methylpent-3-en-1-yl)-2H-c
6-({2-[7,8-dihydroxy-2-methyl-2-(4-methylpent-3-en-1-yl)-2H-chromen-6-yl]-5-hydroxy-4-oxo-3,4-dihydro-2H-1-ben
6-({2-[7,8-dihydroxy-2-methyl-2-(4-methylpent-3-en-1-yl)-2H-chromen-6-yl]-7-hydroxy-4-oxo-3,4-dihydro-2H-1-ben
6-{[6-(5,7-dihydroxy-4-oxo-3,4-dihydro-2H-1-benzopyran-2-yl)-7-hydroxy-2-methyl-2-(4-methylpent-3-en-1-yl)-2H-c
6-{[6-(5,7-dihydroxy-4-oxo-3,4-dihydro-2H-1-benzopyran-2-yl)-5-hydroxy-2-methyl-2-(4-methylpent-3-en-1-yl)-2H-c
6-({2-[5,7-dihydroxy-2-methyl-2-(4-methylpent-3-en-1-yl)-2H-chromen-6-yl]-5-hydroxy-4-oxo-3,4-dihydro-2H-1-ben
6-({2-[5,7-dihydroxy-2-methyl-2-(4-methylpent-3-en-1-yl)-2H-chromen-6-yl]-7-hydroxy-4-oxo-3,4-dihydro-2H-1-ben
Phaeophorbide b
5-Hydroxy-7,3',4'-trimethoxy-8-methylisoflavone 5-neohesperidoside
Egonol gentiobioside
5-Hydroxy-7,3',4'-trimethoxy-8-methylisoflavone 5-neohesperidoside
Egonol gentiobioside
Leukotriene A4
5-HEPE
Oxymesterone
12-HEPE
14,15-EpETE
15-HEPE
15-KETE
17,18-EpETE
5-KETE
11R-HEPE
18R-HEPE
12-KETE
Methyl [8]-Shogaol
(ent-7alpha)-7-Hydroxy-8(14),15-pimaradien-19-oic acid
Galanal A
Ineketone
Steviol
Yucalexin B20
Yucalexin B22

ent-17-Hydroxy-15-kauren-19-oic acid
Yucalexin P17
Yucalexin A19
Aframodial
(ent-15beta)-15-Hydroxy-19-trachylobanoic acid
Grandiflorolic acid
Galanolactone
2-Hydroxy-6-(8-tridecenyl)benzoic acid
(ent-15beta)-15,19-Dihydroxy-7-trachylobanone
12alpha-12-Hydroxy-7,13-abietadien-18-oic acid
Phytocassane C
9-HEPE
Atractyligenin
18R-hydroxy-5Z,8Z,11Z,14Z,16E-eicosapentaenoic acid
15R-hydroxy-5Z,8Z,11Z,13E,17Z-eicosapentaenoic acid
15S-hydroxy-5Z,8Z,11Z,13E,17Z-eicosapentaenoic acid
5S-hydroxy-6E,8Z,11Z,14Z,17Z-eicosapentaenoic acid
(4E)-1-(3,4-dihydroxyphenyl)tetradec-4-en-3-one
3'-Sialyllactose
6'-Sialyllactose
3,4,5-trihydroxy-6-{3-hydroxy-4-[5-hydroxy-3-(3-hydroxy-3-methylbutyl)-8,8-dimethyl-4-oxo-4H,8H-pyrano[2,3-f]ch
3,4,5-trihydroxy-6-{5-hydroxy-2-[5-hydroxy-3-(3-hydroxy-3-methylbutyl)-8,8-dimethyl-4-oxo-4H,8H-pyrano[2,3-f]ch
6-{[2-(2,4-dihydroxyphenyl)-3-(3-hydroxy-3-methylbutyl)-8,8-dimethyl-4-oxo-4H,8H-pyrano[2,3-f]chromen-5-yl]oxy}
3,4,5-trihydroxy-6-({5-hydroxy-2-[5-hydroxy-9-(hydroxymethyl)-13,13-dimethyl-8-oxatetracyclo[7.4.1.0Â²,â0Â¹Â²,
3,4,5-trihydroxy-6-({7-hydroxy-2-[5-hydroxy-9-(hydroxymethyl)-13,13-dimethyl-8-oxatetracyclo[7.4.1.0Â²,â0Â¹Â²,
6-{[4-(5,7-dihydroxy-4-oxo-3,4-dihydro-2H-1-benzopyran-2-yl)-13-(hydroxymethyl)-9,13-dimethyl-8-oxatetracyclo[7
3,4,5-trihydroxy-6-({5-hydroxy-2-[5-hydroxy-13-(hydroxymethyl)-9,13-dimethyl-8-oxatetracyclo[7.4.1.0Â²,â0Â¹Â²,
3,4,5-trihydroxy-6-({7-hydroxy-2-[5-hydroxy-13-(hydroxymethyl)-9,13-dimethyl-8-oxatetracyclo[7.4.1.0Â²,â0Â¹Â²,
6-[(5,7-dihydroxy-2-{5-hydroxy-9,13,13-trimethyl-8-oxatetracyclo[7.4.1.0Â²,â0Â¹Â²,Â¹â]tetradeca-2(7),3,5-trien-4-
3,4,5-trihydroxy-6-{[9,13,13-trimethyl-4-(5,6,7-trihydroxy-4-oxo-3,4-dihydro-2H-1-benzopyran-2-yl)-8-oxatetracyclo
6-[(5,6-dihydroxy-2-{5-hydroxy-9,13,13-trimethyl-8-oxatetracyclo[7.4.1.0Â²,â0Â¹Â²,Â¹â]tetradeca-2(7),3,5-trien-4-
6-[(6,7-dihydroxy-2-{5-hydroxy-9,13,13-trimethyl-8-oxatetracyclo[7.4.1.0Â²,â0Â¹Â²,Â¹â]tetradeca-2(7),3,5-trien-4-
6-{[4-(5,7-dihydroxy-4-oxo-3,4-dihydro-2H-1-benzopyran-2-yl)-10-hydroxy-9,13,13-trimethyl-8-oxatetracyclo[7.4.1.
6-[(2-{5,10-dihydroxy-9,13,13-trimethyl-8-oxatetracyclo[7.4.1.0Â²,â0Â¹Â²,Â¹â]tetradeca-2(7),3,5-trien-4-yl}-5-hyd
6-[(2-{5,10-dihydroxy-9,13,13-trimethyl-8-oxatetracyclo[7.4.1.0Â²,â0Â¹Â²,Â¹â]tetradeca-2(7),3,5-trien-4-yl}-7-hyd
6-{[4-(5,7-dihydroxy-4-oxo-3,4-dihydro-2H-1-benzopyran-2-yl)-11-hydroxy-9,13,13-trimethyl-8-oxatetracyclo[7.4.1.
6-[(2-{5,11-dihydroxy-9,13,13-trimethyl-8-oxatetracyclo[7.4.1.0Â²,â0Â¹Â²,Â¹â]tetradeca-2(7),3,5-trien-4-yl}-5-hyd
6-[(2-{5,11-dihydroxy-9,13,13-trimethyl-8-oxatetracyclo[7.4.1.0Â²,â0Â¹Â²,Â¹â]tetradeca-2(7),3,5-trien-4-yl}-7-hyd
6-{[4-(5,7-dihydroxy-4-oxo-3,4-dihydro-2H-1-benzopyran-2-yl)-5-hydroxy-9,13,13-trimethyl-8-oxatetracyclo[7.4.1.0
6-{[4-(5,7-dihydroxy-4-oxo-3,4-dihydro-2H-1-benzopyran-2-yl)-6-hydroxy-9,13,13-trimethyl-8-oxatetracyclo[7.4.1.0
6-[(2-{5,6-dihydroxy-9,13,13-trimethyl-8-oxatetracyclo[7.4.1.0Â²,â0Â¹Â²,Â¹â]tetradeca-2(7),3,5-trien-4-yl}-5-hydro
6-[(2-{5,6-dihydroxy-9,13,13-trimethyl-8-oxatetracyclo[7.4.1.0Â²,â0Â¹Â²,Â¹â]tetradeca-2(7),3,5-trien-4-yl}-7-hydro
6-{[4-(5,7-dihydroxy-4-oxo-3,4-dihydro-2H-1-benzopyran-2-yl)-3-hydroxy-9,13,13-trimethyl-8-oxatetracyclo[7.4.1.0
6-[(2-{3,5-dihydroxy-9,13,13-trimethyl-8-oxatetracyclo[7.4.1.0Â²,â0Â¹Â²,Â¹â]tetradeca-2(7),3,5-trien-4-yl}-5-hydro
6-[(2-{3,5-dihydroxy-9,13,13-trimethyl-8-oxatetracyclo[7.4.1.0Â²,â0Â¹Â²,Â¹â]tetradeca-2(7),3,5-trien-4-yl}-7-hydro
6-{[6-(5,7-dihydroxy-4-oxo-3,4-dihydro-2H-1-benzopyran-2-yl)-2-(hydroxymethyl)-2-(4-methylpent-3-en-1-yl)-2H-ch
3,4,5-trihydroxy-6-({5-hydroxy-2-[7-hydroxy-2-(hydroxymethyl)-2-(4-methylpent-3-en-1-yl)-2H-chromen-6-yl]-4-oxo
3,4,5-trihydroxy-6-({7-hydroxy-2-[7-hydroxy-2-(hydroxymethyl)-2-(4-methylpent-3-en-1-yl)-2H-chromen-6-yl]-4-oxo
6-{[6-(5,7-dihydroxy-4-oxo-3,4-dihydro-2H-1-benzopyran-2-yl)-2-(1-hydroxy-4-methylpent-3-en-1-yl)-2-methyl-2H-c
3,4,5-trihydroxy-6-({5-hydroxy-2-[7-hydroxy-2-(1-hydroxy-4-methylpent-3-en-1-yl)-2-methyl-2H-chromen-6-yl]-4-ox

3,4,5-trihydroxy-6-({7-hydroxy-2-[7-hydroxy-2-(1-hydroxy-4-methylpent-3-en-1-yl)-2-methyl-2H-chromen-6-yl]-4-ox
6-({5,7-dihydroxy-2-[7-hydroxy-2-methyl-2-(4-methylpent-3-en-1-yl)-2H-chromen-6-yl]-4-oxo-3,4-dihydro-2H-1-ben
3,4,5-trihydroxy-6-{[2-methyl-2-(4-methylpent-3-en-1-yl)-6-(5,6,7-trihydroxy-4-oxo-3,4-dihydro-2H-1-benzopyran-2-
6-({5,6-dihydroxy-2-[7-hydroxy-2-methyl-2-(4-methylpent-3-en-1-yl)-2H-chromen-6-yl]-4-oxo-3,4-dihydro-2H-1-ben
6-({6,7-dihydroxy-2-[7-hydroxy-2-methyl-2-(4-methylpent-3-en-1-yl)-2H-chromen-6-yl]-4-oxo-3,4-dihydro-2H-1-ben
6-{[6-(5,7-dihydroxy-4-oxo-3,4-dihydro-2H-1-benzopyran-2-yl)-2-(5-hydroxy-4-methylpent-3-en-1-yl)-2-methyl-2H-c
3,4,5-trihydroxy-6-({5-hydroxy-2-[7-hydroxy-2-(5-hydroxy-4-methylpent-3-en-1-yl)-2-methyl-2H-chromen-6-yl]-4-ox
3,4,5-trihydroxy-6-({7-hydroxy-2-[7-hydroxy-2-(5-hydroxy-4-methylpent-3-en-1-yl)-2-methyl-2H-chromen-6-yl]-4-ox
3,4,5-trihydroxy-6-[(5-hydroxy-2-{7-hydroxy-2-[(3E)-5-hydroxy-4-methylpent-3-en-1-yl]-2-methyl-2H-chromen-6-yl}-
6-{[6-(5,7-dihydroxy-4-oxo-3,4-dihydro-2H-1-benzopyran-2-yl)-2-(2-hydroxy-4-methylpent-3-en-1-yl)-2-methyl-2H-c
3,4,5-trihydroxy-6-({5-hydroxy-2-[7-hydroxy-2-(2-hydroxy-4-methylpent-3-en-1-yl)-2-methyl-2H-chromen-6-yl]-4-ox
3,4,5-trihydroxy-6-({7-hydroxy-2-[7-hydroxy-2-(2-hydroxy-4-methylpent-3-en-1-yl)-2-methyl-2H-chromen-6-yl]-4-ox
6-{[6-(5,7-dihydroxy-4-oxo-3,4-dihydro-2H-1-benzopyran-2-yl)-2-[2-(3,3-dimethyloxiran-2-yl)ethyl]-2-methyl-2H-chr
6-[(2-{2-[2-(3,3-dimethyloxiran-2-yl)ethyl]-7-hydroxy-2-methyl-2H-chromen-6-yl}-5-hydroxy-4-oxo-3,4-dihydro-2H-1
6-[(2-{2-[2-(3,3-dimethyloxiran-2-yl)ethyl]-7-hydroxy-2-methyl-2H-chromen-6-yl}-7-hydroxy-4-oxo-3,4-dihydro-2H-1
6-{[10-(5,7-dihydroxy-4-oxo-3,4-dihydro-2H-1-benzopyran-2-yl)-5-methyl-5-(4-methylpent-3-en-1-yl)-3,6-dioxatricyc
3,4,5-trihydroxy-6-({5-hydroxy-2-[9-hydroxy-5-methyl-5-(4-methylpent-3-en-1-yl)-3,6-dioxatricyclo[5.4.0.0Â²,â�und
3,4,5-trihydroxy-6-({7-hydroxy-2-[9-hydroxy-5-methyl-5-(4-methylpent-3-en-1-yl)-3,6-dioxatricyclo[5.4.0.0Â²,â�und
6-{[6-(5,7-dihydroxy-4-oxo-3,4-dihydro-2H-1-benzopyran-2-yl)-7-hydroxy-2-methyl-2-(4-methylpent-3-en-1-yl)-2H-c
6-{[6-(5,7-dihydroxy-4-oxo-3,4-dihydro-2H-1-benzopyran-2-yl)-8-hydroxy-2-methyl-2-(4-methylpent-3-en-1-yl)-2H-c
6-({2-[7,8-dihydroxy-2-methyl-2-(4-methylpent-3-en-1-yl)-2H-chromen-6-yl]-5-hydroxy-4-oxo-3,4-dihydro-2H-1-ben
6-({2-[7,8-dihydroxy-2-methyl-2-(4-methylpent-3-en-1-yl)-2H-chromen-6-yl]-7-hydroxy-4-oxo-3,4-dihydro-2H-1-ben
6-{[6-(5,7-dihydroxy-4-oxo-3,4-dihydro-2H-1-benzopyran-2-yl)-7-hydroxy-2-methyl-2-(4-methylpent-3-en-1-yl)-2H-c
6-{[6-(5,7-dihydroxy-4-oxo-3,4-dihydro-2H-1-benzopyran-2-yl)-5-hydroxy-2-methyl-2-(4-methylpent-3-en-1-yl)-2H-c
6-({2-[5,7-dihydroxy-2-methyl-2-(4-methylpent-3-en-1-yl)-2H-chromen-6-yl]-5-hydroxy-4-oxo-3,4-dihydro-2H-1-ben
6-({2-[5,7-dihydroxy-2-methyl-2-(4-methylpent-3-en-1-yl)-2H-chromen-6-yl]-7-hydroxy-4-oxo-3,4-dihydro-2H-1-ben
3,4,5-trihydroxy-6-{3-hydroxy-4-[5-hydroxy-3-(3-hydroxy-3-methylbutyl)-8,8-dimethyl-4-oxo-4H,8H-pyrano[2,3-f]chr
3,4,5-trihydroxy-6-{5-hydroxy-2-[5-hydroxy-3-(3-hydroxy-3-methylbutyl)-8,8-dimethyl-4-oxo-4H,8H-pyrano[2,3-f]chr
6-{[2-(2,4-dihydroxyphenyl)-3-(3-hydroxy-3-methylbutyl)-8,8-dimethyl-4-oxo-4H,8H-pyrano[2,3-f]chromen-5-yl]oxy}
3,4,5-trihydroxy-6-({5-hydroxy-2-[5-hydroxy-9-(hydroxymethyl)-13,13-dimethyl-8-oxatetracyclo[7.4.1.0Â²,â�0Â¹Â²,Â
3,4,5-trihydroxy-6-({7-hydroxy-2-[5-hydroxy-9-(hydroxymethyl)-13,13-dimethyl-8-oxatetracyclo[7.4.1.0Â²,â�0Â¹Â²,Â
6-{[4-(5,7-dihydroxy-4-oxo-3,4-dihydro-2H-1-benzopyran-2-yl)-13-(hydroxymethyl)-9,13-dimethyl-8-oxatetracyclo[7
3,4,5-trihydroxy-6-({5-hydroxy-2-[5-hydroxy-13-(hydroxymethyl)-9,13-dimethyl-8-oxatetracyclo[7.4.1.0Â²,â�0Â¹Â²,Â
3,4,5-trihydroxy-6-({7-hydroxy-2-[5-hydroxy-13-(hydroxymethyl)-9,13-dimethyl-8-oxatetracyclo[7.4.1.0Â²,â�0Â¹Â²,Â
6-[(5,7-dihydroxy-2-{5-hydroxy-9,13,13-trimethyl-8-oxatetracyclo[7.4.1.0Â²,â�0Â¹Â²,Â¹â�tetradeca-2(7),3,5-trien-4-
3,4,5-trihydroxy-6-{[9,13,13-trimethyl-4-(5,6,7-trihydroxy-4-oxo-3,4-dihydro-2H-1-benzopyran-2-yl)-8-oxatetracyclo
6-[(5,6-dihydroxy-2-{5-hydroxy-9,13,13-trimethyl-8-oxatetracyclo[7.4.1.0Â²,â�0Â¹Â²,Â¹â�tetradeca-2(7),3,5-trien-4-
6-[(6,7-dihydroxy-2-{5-hydroxy-9,13,13-trimethyl-8-oxatetracyclo[7.4.1.0Â²,â�0Â¹Â²,Â¹â�tetradeca-2(7),3,5-trien-4-
6-{[4-(5,7-dihydroxy-4-oxo-3,4-dihydro-2H-1-benzopyran-2-yl)-10-hydroxy-9,13,13-trimethyl-8-oxatetracyclo[7.4.1.
6-[(2-{5,10-dihydroxy-9,13,13-trimethyl-8-oxatetracyclo[7.4.1.0Â²,â�0Â¹Â²,Â¹â�tetradeca-2(7),3,5-trien-4-yl}-5-hydr
6-[(2-{5,10-dihydroxy-9,13,13-trimethyl-8-oxatetracyclo[7.4.1.0Â²,â�0Â¹Â²,Â¹â�tetradeca-2(7),3,5-trien-4-yl}-7-hydr
6-{[4-(5,7-dihydroxy-4-oxo-3,4-dihydro-2H-1-benzopyran-2-yl)-11-hydroxy-9,13,13-trimethyl-8-oxatetracyclo[7.4.1.
6-[(2-{5,11-dihydroxy-9,13,13-trimethyl-8-oxatetracyclo[7.4.1.0Â²,â�0Â¹Â²,Â¹â�tetradeca-2(7),3,5-trien-4-yl}-5-hydr
6-[(2-{5,11-dihydroxy-9,13,13-trimethyl-8-oxatetracyclo[7.4.1.0Â²,â�0Â¹Â²,Â¹â�tetradeca-2(7),3,5-trien-4-yl}-7-hydr
6-{[4-(5,7-dihydroxy-4-oxo-3,4-dihydro-2H-1-benzopyran-2-yl)-5-hydroxy-9,13,13-trimethyl-8-oxatetracyclo[7.4.1.0
6-{[4-(5,7-dihydroxy-4-oxo-3,4-dihydro-2H-1-benzopyran-2-yl)-6-hydroxy-9,13,13-trimethyl-8-oxatetracyclo[7.4.1.0
6-[(2-{5,6-dihydroxy-9,13,13-trimethyl-8-oxatetracyclo[7.4.1.0Â²,â�0Â¹Â²,Â¹â�tetradeca-2(7),3,5-trien-4-yl}-5-hydro
6-[(2-{5,6-dihydroxy-9,13,13-trimethyl-8-oxatetracyclo[7.4.1.0Â²,â�0Â¹Â²,Â¹â�tetradeca-2(7),3,5-trien-4-yl}-7-hydro
6-{[4-(5,7-dihydroxy-4-oxo-3,4-dihydro-2H-1-benzopyran-2-yl)-3-hydroxy-9,13,13-trimethyl-8-oxatetracyclo[7.4.1.0
6-[(2-{3,5-dihydroxy-9,13,13-trimethyl-8-oxatetracyclo[7.4.1.0Â²,â�0Â¹Â²,Â¹â�tetradeca-2(7),3,5-trien-4-yl}-5-hydro

6-[(2-{3,5-dihydroxy-9,13,13-trimethyl-8-oxatetracyclo[7.4.1.0Â²,â�0Â¹Â²,Â¹â�]tetradeca-2(7),3,5-trien-4-yl}-7-hydro
6-{[6-(5,7-dihydroxy-4-oxo-3,4-dihydro-2H-1-benzopyran-2-yl)-2-(hydroxymethyl)-2-(4-methylpent-3-en-1-yl)-2H-ch
3,4,5-trihydroxy-6-({5-hydroxy-2-[7-hydroxy-2-(hydroxymethyl)-2-(4-methylpent-3-en-1-yl)-2H-chromen-6-yl]-4-oxo
3,4,5-trihydroxy-6-({7-hydroxy-2-[7-hydroxy-2-(hydroxymethyl)-2-(4-methylpent-3-en-1-yl)-2H-chromen-6-yl]-4-oxo
6-{[6-(5,7-dihydroxy-4-oxo-3,4-dihydro-2H-1-benzopyran-2-yl)-2-(1-hydroxy-4-methylpent-3-en-1-yl)-2-methyl-2H-c
3,4,5-trihydroxy-6-({5-hydroxy-2-[7-hydroxy-2-(1-hydroxy-4-methylpent-3-en-1-yl)-2-methyl-2H-chromen-6-yl]-4-ox
3,4,5-trihydroxy-6-({7-hydroxy-2-[7-hydroxy-2-(1-hydroxy-4-methylpent-3-en-1-yl)-2-methyl-2H-chromen-6-yl]-4-ox
6-({5,7-dihydroxy-2-[7-hydroxy-2-methyl-2-(4-methylpent-3-en-1-yl)-2H-chromen-6-yl]-4-oxo-3,4-dihydro-2H-1-ben
3,4,5-trihydroxy-6-{[2-methyl-2-(4-methylpent-3-en-1-yl)-6-(5,6,7-trihydroxy-4-oxo-3,4-dihydro-2H-1-benzopyran-2-
6-({5,6-dihydroxy-2-[7-hydroxy-2-methyl-2-(4-methylpent-3-en-1-yl)-2H-chromen-6-yl]-4-oxo-3,4-dihydro-2H-1-ben
6-({6,7-dihydroxy-2-[7-hydroxy-2-methyl-2-(4-methylpent-3-en-1-yl)-2H-chromen-6-yl]-4-oxo-3,4-dihydro-2H-1-ben
6-{[6-(5,7-dihydroxy-4-oxo-3,4-dihydro-2H-1-benzopyran-2-yl)-2-(5-hydroxy-4-methylpent-3-en-1-yl)-2-methyl-2H-c
3,4,5-trihydroxy-6-({5-hydroxy-2-[7-hydroxy-2-(5-hydroxy-4-methylpent-3-en-1-yl)-2-methyl-2H-chromen-6-yl]-4-ox
3,4,5-trihydroxy-6-({7-hydroxy-2-[7-hydroxy-2-(5-hydroxy-4-methylpent-3-en-1-yl)-2-methyl-2H-chromen-6-yl]-4-ox
3,4,5-trihydroxy-6-[(5-hydroxy-2-{7-hydroxy-2-[(3E)-5-hydroxy-4-methylpent-3-en-1-yl]-2-methyl-2H-chromen-6-yl}-
6-{[6-(5,7-dihydroxy-4-oxo-3,4-dihydro-2H-1-benzopyran-2-yl)-2-(2-hydroxy-4-methylpent-3-en-1-yl)-2-methyl-2H-c
3,4,5-trihydroxy-6-({5-hydroxy-2-[7-hydroxy-2-(2-hydroxy-4-methylpent-3-en-1-yl)-2-methyl-2H-chromen-6-yl]-4-ox
3,4,5-trihydroxy-6-({7-hydroxy-2-[7-hydroxy-2-(2-hydroxy-4-methylpent-3-en-1-yl)-2-methyl-2H-chromen-6-yl]-4-ox
6-{[6-(5,7-dihydroxy-4-oxo-3,4-dihydro-2H-1-benzopyran-2-yl)-2-[2-(3,3-dimethyloxiran-2-yl)ethyl]-2-methyl-2H-chr
6-[(2-{2-[2-(3,3-dimethyloxiran-2-yl)ethyl]-7-hydroxy-2-methyl-2H-chromen-6-yl}-5-hydroxy-4-oxo-3,4-dihydro-2H-1
6-[(2-{2-[2-(3,3-dimethyloxiran-2-yl)ethyl]-7-hydroxy-2-methyl-2H-chromen-6-yl}-7-hydroxy-4-oxo-3,4-dihydro-2H-1
6-{[10-(5,7-dihydroxy-4-oxo-3,4-dihydro-2H-1-benzopyran-2-yl)-5-methyl-5-(4-methylpent-3-en-1-yl)-3,6-dioxatricyc
3,4,5-trihydroxy-6-({5-hydroxy-2-[9-hydroxy-5-methyl-5-(4-methylpent-3-en-1-yl)-3,6-dioxatricyclo[5.4.0.0Â²,â�]und
3,4,5-trihydroxy-6-({7-hydroxy-2-[9-hydroxy-5-methyl-5-(4-methylpent-3-en-1-yl)-3,6-dioxatricyclo[5.4.0.0Â²,â�]und
6-{[6-(5,7-dihydroxy-4-oxo-3,4-dihydro-2H-1-benzopyran-2-yl)-7-hydroxy-2-methyl-2-(4-methylpent-3-en-1-yl)-2H-c
6-{[6-(5,7-dihydroxy-4-oxo-3,4-dihydro-2H-1-benzopyran-2-yl)-8-hydroxy-2-methyl-2-(4-methylpent-3-en-1-yl)-2H-c
6-({2-[7,8-dihydroxy-2-methyl-2-(4-methylpent-3-en-1-yl)-2H-chromen-6-yl]-5-hydroxy-4-oxo-3,4-dihydro-2H-1-ben
6-({2-[7,8-dihydroxy-2-methyl-2-(4-methylpent-3-en-1-yl)-2H-chromen-6-yl]-7-hydroxy-4-oxo-3,4-dihydro-2H-1-ben
6-{[6-(5,7-dihydroxy-4-oxo-3,4-dihydro-2H-1-benzopyran-2-yl)-7-hydroxy-2-methyl-2-(4-methylpent-3-en-1-yl)-2H-c
6-{[6-(5,7-dihydroxy-4-oxo-3,4-dihydro-2H-1-benzopyran-2-yl)-5-hydroxy-2-methyl-2-(4-methylpent-3-en-1-yl)-2H-c
6-({2-[5,7-dihydroxy-2-methyl-2-(4-methylpent-3-en-1-yl)-2H-chromen-6-yl]-5-hydroxy-4-oxo-3,4-dihydro-2H-1-ben
6-({2-[5,7-dihydroxy-2-methyl-2-(4-methylpent-3-en-1-yl)-2H-chromen-6-yl]-7-hydroxy-4-oxo-3,4-dihydro-2H-1-ben
Enalapril
Remifentanil
Raloxifene
4-Nitrocatechol
2,4-Dihydroxy-nitrophenol
2-hydroxy-4-nitrophenolate
3'-Sialyllactose
6'-Sialyllactose
3,4,5-trihydroxy-6-{3-hydroxy-4-[5-hydroxy-3-(3-hydroxy-3-methylbutyl)-8,8-dimethyl-4-oxo-4H,8H-pyrano[2,3-f]chr
3,4,5-trihydroxy-6-{5-hydroxy-2-[5-hydroxy-3-(3-hydroxy-3-methylbutyl)-8,8-dimethyl-4-oxo-4H,8H-pyrano[2,3-f]chr
6-{[2-(2,4-dihydroxyphenyl)-3-(3-hydroxy-3-methylbutyl)-8,8-dimethyl-4-oxo-4H,8H-pyrano[2,3-f]chromen-5-yl]oxy}
3,4,5-trihydroxy-6-({5-hydroxy-2-[5-hydroxy-9-(hydroxymethyl)-13,13-dimethyl-8-oxatetracyclo[7.4.1.0Â²,â�0Â¹Â²,Â
3,4,5-trihydroxy-6-({7-hydroxy-2-[5-hydroxy-9-(hydroxymethyl)-13,13-dimethyl-8-oxatetracyclo[7.4.1.0Â²,â�0Â¹Â²,Â
6-{[4-(5,7-dihydroxy-4-oxo-3,4-dihydro-2H-1-benzopyran-2-yl)-13-(hydroxymethyl)-9,13-dimethyl-8-oxatetracyclo[7
3,4,5-trihydroxy-6-({5-hydroxy-2-[5-hydroxy-13-(hydroxymethyl)-9,13-dimethyl-8-oxatetracyclo[7.4.1.0Â²,â�0Â¹Â²,Â
3,4,5-trihydroxy-6-({7-hydroxy-2-[5-hydroxy-13-(hydroxymethyl)-9,13-dimethyl-8-oxatetracyclo[7.4.1.0Â²,â�0Â¹Â²,Â
6-[(5,7-dihydroxy-2-{5-hydroxy-9,13,13-trimethyl-8-oxatetracyclo[7.4.1.0Â²,â�0Â¹Â²,Â¹â�]tetradeca-2(7),3,5-trien-4-
3,4,5-trihydroxy-6-{[9,13,13-trimethyl-4-(5,6,7-trihydroxy-4-oxo-3,4-dihydro-2H-1-benzopyran-2-yl)-8-oxatetracyclo

6-[(5,6-dihydroxy-2-{5-hydroxy-9,13,13-trimethyl-8-oxatetracyclo[7.4.1.0Â²,â0Â¹Â²,Â¹â]tetradeca-2(7),3,5-trien-4-
6-[(6,7-dihydroxy-2-{5-hydroxy-9,13,13-trimethyl-8-oxatetracyclo[7.4.1.0Â²,â0Â¹Â²,Â¹â]tetradeca-2(7),3,5-trien-4-
6-{[4-(5,7-dihydroxy-4-oxo-3,4-dihydro-2H-1-benzopyran-2-yl)-10-hydroxy-9,13,13-trimethyl-8-oxatetracyclo[7.4.1.
6-[(2-{5,10-dihydroxy-9,13,13-trimethyl-8-oxatetracyclo[7.4.1.0Â²,â0Â¹Â²,Â¹â]tetradeca-2(7),3,5-trien-4-yl}-5-hydr
6-[(2-{5,10-dihydroxy-9,13,13-trimethyl-8-oxatetracyclo[7.4.1.0Â²,â0Â¹Â²,Â¹â]tetradeca-2(7),3,5-trien-4-yl}-7-hydr
6-{[4-(5,7-dihydroxy-4-oxo-3,4-dihydro-2H-1-benzopyran-2-yl)-11-hydroxy-9,13,13-trimethyl-8-oxatetracyclo[7.4.1.
6-[(2-{5,11-dihydroxy-9,13,13-trimethyl-8-oxatetracyclo[7.4.1.0Â²,â0Â¹Â²,Â¹â]tetradeca-2(7),3,5-trien-4-yl}-5-hydr
6-[(2-{5,11-dihydroxy-9,13,13-trimethyl-8-oxatetracyclo[7.4.1.0Â²,â0Â¹Â²,Â¹â]tetradeca-2(7),3,5-trien-4-yl}-7-hydr
6-{[4-(5,7-dihydroxy-4-oxo-3,4-dihydro-2H-1-benzopyran-2-yl)-5-hydroxy-9,13,13-trimethyl-8-oxatetracyclo[7.4.1.0
6-{[4-(5,7-dihydroxy-4-oxo-3,4-dihydro-2H-1-benzopyran-2-yl)-6-hydroxy-9,13,13-trimethyl-8-oxatetracyclo[7.4.1.0
6-[(2-{5,6-dihydroxy-9,13,13-trimethyl-8-oxatetracyclo[7.4.1.0Â²,â0Â¹Â²,Â¹â]tetradeca-2(7),3,5-trien-4-yl}-5-hydro
6-[(2-{5,6-dihydroxy-9,13,13-trimethyl-8-oxatetracyclo[7.4.1.0Â²,â0Â¹Â²,Â¹â]tetradeca-2(7),3,5-trien-4-yl}-7-hydro
6-{[4-(5,7-dihydroxy-4-oxo-3,4-dihydro-2H-1-benzopyran-2-yl)-3-hydroxy-9,13,13-trimethyl-8-oxatetracyclo[7.4.1.0
6-[(2-{3,5-dihydroxy-9,13,13-trimethyl-8-oxatetracyclo[7.4.1.0Â²,â0Â¹Â²,Â¹â]tetradeca-2(7),3,5-trien-4-yl}-5-hydro
6-[(2-{3,5-dihydroxy-9,13,13-trimethyl-8-oxatetracyclo[7.4.1.0Â²,â0Â¹Â²,Â¹â]tetradeca-2(7),3,5-trien-4-yl}-7-hydro
6-{[6-(5,7-dihydroxy-4-oxo-3,4-dihydro-2H-1-benzopyran-2-yl)-2-(hydroxymethyl)-2-(4-methylpent-3-en-1-yl)-2H-ch
3,4,5-trihydroxy-6-({5-hydroxy-2-[7-hydroxy-2-(hydroxymethyl)-2-(4-methylpent-3-en-1-yl)-2H-chromen-6-yl]-4-oxo
3,4,5-trihydroxy-6-({7-hydroxy-2-[7-hydroxy-2-(hydroxymethyl)-2-(4-methylpent-3-en-1-yl)-2H-chromen-6-yl]-4-oxo
6-{[6-(5,7-dihydroxy-4-oxo-3,4-dihydro-2H-1-benzopyran-2-yl)-2-(1-hydroxy-4-methylpent-3-en-1-yl)-2-methyl-2H-c
3,4,5-trihydroxy-6-({5-hydroxy-2-[7-hydroxy-2-(1-hydroxy-4-methylpent-3-en-1-yl)-2-methyl-2H-chromen-6-yl]-4-ox
3,4,5-trihydroxy-6-({7-hydroxy-2-[7-hydroxy-2-(1-hydroxy-4-methylpent-3-en-1-yl)-2-methyl-2H-chromen-6-yl]-4-ox
6-({5,7-dihydroxy-2-[7-hydroxy-2-methyl-2-(4-methylpent-3-en-1-yl)-2H-chromen-6-yl]-4-oxo-3,4-dihydro-2H-1-ben
3,4,5-trihydroxy-6-{[2-methyl-2-(4-methylpent-3-en-1-yl)-6-(5,6,7-trihydroxy-4-oxo-3,4-dihydro-2H-1-benzopyran-2-
6-({5,6-dihydroxy-2-[7-hydroxy-2-methyl-2-(4-methylpent-3-en-1-yl)-2H-chromen-6-yl]-4-oxo-3,4-dihydro-2H-1-ben
6-({6,7-dihydroxy-2-[7-hydroxy-2-methyl-2-(4-methylpent-3-en-1-yl)-2H-chromen-6-yl]-4-oxo-3,4-dihydro-2H-1-ben
6-{[6-(5,7-dihydroxy-4-oxo-3,4-dihydro-2H-1-benzopyran-2-yl)-2-(5-hydroxy-4-methylpent-3-en-1-yl)-2-methyl-2H-c
3,4,5-trihydroxy-6-({5-hydroxy-2-[7-hydroxy-2-(5-hydroxy-4-methylpent-3-en-1-yl)-2-methyl-2H-chromen-6-yl]-4-ox
3,4,5-trihydroxy-6-({7-hydroxy-2-[7-hydroxy-2-(5-hydroxy-4-methylpent-3-en-1-yl)-2-methyl-2H-chromen-6-yl]-4-ox
3,4,5-trihydroxy-6-[(5-hydroxy-2-{7-hydroxy-2-[(3E)-5-hydroxy-4-methylpent-3-en-1-yl]-2-methyl-2H-chromen-6-yl}-
6-{[6-(5,7-dihydroxy-4-oxo-3,4-dihydro-2H-1-benzopyran-2-yl)-2-(2-hydroxy-4-methylpent-3-en-1-yl)-2-methyl-2H-c
3,4,5-trihydroxy-6-({5-hydroxy-2-[7-hydroxy-2-(2-hydroxy-4-methylpent-3-en-1-yl)-2-methyl-2H-chromen-6-yl]-4-ox
3,4,5-trihydroxy-6-({7-hydroxy-2-[7-hydroxy-2-(2-hydroxy-4-methylpent-3-en-1-yl)-2-methyl-2H-chromen-6-yl]-4-ox
6-{[6-(5,7-dihydroxy-4-oxo-3,4-dihydro-2H-1-benzopyran-2-yl)-2-[2-(3,3-dimethyloxiran-2-yl)ethyl]-2-methyl-2H-chr
6-[(2-{2-[2-(3,3-dimethyloxiran-2-yl)ethyl]-7-hydroxy-2-methyl-2H-chromen-6-yl}-5-hydroxy-4-oxo-3,4-dihydro-2H-1
6-[(2-{2-[2-(3,3-dimethyloxiran-2-yl)ethyl]-7-hydroxy-2-methyl-2H-chromen-6-yl}-7-hydroxy-4-oxo-3,4-dihydro-2H-1
6-{[10-(5,7-dihydroxy-4-oxo-3,4-dihydro-2H-1-benzopyran-2-yl)-5-methyl-5-(4-methylpent-3-en-1-yl)-3,6-dioxatricy
3,4,5-trihydroxy-6-({5-hydroxy-2-[9-hydroxy-5-methyl-5-(4-methylpent-3-en-1-yl)-3,6-dioxatricyclo[5.4.0.0Â²,â]und
3,4,5-trihydroxy-6-({7-hydroxy-2-[9-hydroxy-5-methyl-5-(4-methylpent-3-en-1-yl)-3,6-dioxatricyclo[5.4.0.0Â²,â]und
6-{[6-(5,7-dihydroxy-4-oxo-3,4-dihydro-2H-1-benzopyran-2-yl)-7-hydroxy-2-methyl-2-(4-methylpent-3-en-1-yl)-2H-c
6-{[6-(5,7-dihydroxy-4-oxo-3,4-dihydro-2H-1-benzopyran-2-yl)-8-hydroxy-2-methyl-2-(4-methylpent-3-en-1-yl)-2H-c
6-({2-[7,8-dihydroxy-2-methyl-2-(4-methylpent-3-en-1-yl)-2H-chromen-6-yl]-5-hydroxy-4-oxo-3,4-dihydro-2H-1-ben
6-({2-[7,8-dihydroxy-2-methyl-2-(4-methylpent-3-en-1-yl)-2H-chromen-6-yl]-7-hydroxy-4-oxo-3,4-dihydro-2H-1-ben
6-{[6-(5,7-dihydroxy-4-oxo-3,4-dihydro-2H-1-benzopyran-2-yl)-7-hydroxy-2-methyl-2-(4-methylpent-3-en-1-yl)-2H-c
6-{[6-(5,7-dihydroxy-4-oxo-3,4-dihydro-2H-1-benzopyran-2-yl)-5-hydroxy-2-methyl-2-(4-methylpent-3-en-1-yl)-2H-c
6-({2-[5,7-dihydroxy-2-methyl-2-(4-methylpent-3-en-1-yl)-2H-chromen-6-yl]-5-hydroxy-4-oxo-3,4-dihydro-2H-1-ben
6-({2-[5,7-dihydroxy-2-methyl-2-(4-methylpent-3-en-1-yl)-2H-chromen-6-yl]-7-hydroxy-4-oxo-3,4-dihydro-2H-1-ben
3,4,5-trihydroxy-6-{3-hydroxy-4-[5-hydroxy-3-(3-hydroxy-3-methylbutyl)-8,8-dimethyl-4-oxo-4H,8H-pyrano[2,3-f]chr
3,4,5-trihydroxy-6-{5-hydroxy-2-[5-hydroxy-3-(3-hydroxy-3-methylbutyl)-8,8-dimethyl-4-oxo-4H,8H-pyrano[2,3-f]chr
6-{[2-(2,4-dihydroxyphenyl)-3-(3-hydroxy-3-methylbutyl)-8,8-dimethyl-4-oxo-4H,8H-pyrano[2,3-f]chromen-5-yl]oxy}
3,4,5-trihydroxy-6-({5-hydroxy-2-[5-hydroxy-9-(hydroxymethyl)-13,13-dimethyl-8-oxatetracyclo[7.4.1.0Â²,â0Â¹Â²,Â

3,4,5-trihydroxy-6-({7-hydroxy-2-[5-hydroxy-9-(hydroxymethyl)-13,13-dimethyl-8-oxatetracyclo[7.4.1.0Â²,â�0Â¹Â²,Â
6-{[4-(5,7-dihydroxy-4-oxo-3,4-dihydro-2H-1-benzopyran-2-yl)-13-(hydroxymethyl)-9,13-dimethyl-8-oxatetracyclo[7
3,4,5-trihydroxy-6-({5-hydroxy-2-[5-hydroxy-13-(hydroxymethyl)-9,13-dimethyl-8-oxatetracyclo[7.4.1.0Â²,â�0Â¹Â²,Â
3,4,5-trihydroxy-6-({7-hydroxy-2-[5-hydroxy-13-(hydroxymethyl)-9,13-dimethyl-8-oxatetracyclo[7.4.1.0Â²,â�0Â¹Â²,Â
6-[(5,7-dihydroxy-2-{5-hydroxy-9,13,13-trimethyl-8-oxatetracyclo[7.4.1.0Â²,â�0Â¹Â²,Â¹â�]tetradeca-2(7),3,5-trien-4-
3,4,5-trihydroxy-6-{[9,13,13-trimethyl-4-(5,6,7-trihydroxy-4-oxo-3,4-dihydro-2H-1-benzopyran-2-yl)-8-oxatetracyclo
6-[(5,6-dihydroxy-2-{5-hydroxy-9,13,13-trimethyl-8-oxatetracyclo[7.4.1.0Â²,â�0Â¹Â²,Â¹â�]tetradeca-2(7),3,5-trien-4-
6-[(6,7-dihydroxy-2-{5-hydroxy-9,13,13-trimethyl-8-oxatetracyclo[7.4.1.0Â²,â�0Â¹Â²,Â¹â�]tetradeca-2(7),3,5-trien-4-
6-{[4-(5,7-dihydroxy-4-oxo-3,4-dihydro-2H-1-benzopyran-2-yl)-10-hydroxy-9,13,13-trimethyl-8-oxatetracyclo[7.4.1.0
6-[(2-{5,10-dihydroxy-9,13,13-trimethyl-8-oxatetracyclo[7.4.1.0Â²,â�0Â¹Â²,Â¹â�]tetradeca-2(7),3,5-trien-4-yl}-5-hydr
6-[(2-{5,10-dihydroxy-9,13,13-trimethyl-8-oxatetracyclo[7.4.1.0Â²,â�0Â¹Â²,Â¹â�]tetradeca-2(7),3,5-trien-4-yl}-7-hydr
6-{[4-(5,7-dihydroxy-4-oxo-3,4-dihydro-2H-1-benzopyran-2-yl)-11-hydroxy-9,13,13-trimethyl-8-oxatetracyclo[7.4.1.0
6-[(2-{5,11-dihydroxy-9,13,13-trimethyl-8-oxatetracyclo[7.4.1.0Â²,â�0Â¹Â²,Â¹â�]tetradeca-2(7),3,5-trien-4-yl}-5-hydr
6-[(2-{5,11-dihydroxy-9,13,13-trimethyl-8-oxatetracyclo[7.4.1.0Â²,â�0Â¹Â²,Â¹â�]tetradeca-2(7),3,5-trien-4-yl}-7-hydr
6-{[4-(5,7-dihydroxy-4-oxo-3,4-dihydro-2H-1-benzopyran-2-yl)-5-hydroxy-9,13,13-trimethyl-8-oxatetracyclo[7.4.1.0
6-{[4-(5,7-dihydroxy-4-oxo-3,4-dihydro-2H-1-benzopyran-2-yl)-6-hydroxy-9,13,13-trimethyl-8-oxatetracyclo[7.4.1.0
6-[(2-{5,6-dihydroxy-9,13,13-trimethyl-8-oxatetracyclo[7.4.1.0Â²,â�0Â¹Â²,Â¹â�]tetradeca-2(7),3,5-trien-4-yl}-5-hydro
6-[(2-{5,6-dihydroxy-9,13,13-trimethyl-8-oxatetracyclo[7.4.1.0Â²,â�0Â¹Â²,Â¹â�]tetradeca-2(7),3,5-trien-4-yl}-7-hydro
6-{[4-(5,7-dihydroxy-4-oxo-3,4-dihydro-2H-1-benzopyran-2-yl)-3-hydroxy-9,13,13-trimethyl-8-oxatetracyclo[7.4.1.0
6-[(2-{3,5-dihydroxy-9,13,13-trimethyl-8-oxatetracyclo[7.4.1.0Â²,â�0Â¹Â²,Â¹â�]tetradeca-2(7),3,5-trien-4-yl}-5-hydro
6-[(2-{3,5-dihydroxy-9,13,13-trimethyl-8-oxatetracyclo[7.4.1.0Â²,â�0Â¹Â²,Â¹â�]tetradeca-2(7),3,5-trien-4-yl}-7-hydro
6-{[6-(5,7-dihydroxy-4-oxo-3,4-dihydro-2H-1-benzopyran-2-yl)-2-(hydroxymethyl)-2-(4-methylpent-3-en-1-yl)-2H-ch
3,4,5-trihydroxy-6-({5-hydroxy-2-[7-hydroxy-2-(hydroxymethyl)-2-(4-methylpent-3-en-1-yl)-2H-chromen-6-yl]-4-oxo
3,4,5-trihydroxy-6-({7-hydroxy-2-[7-hydroxy-2-(hydroxymethyl)-2-(4-methylpent-3-en-1-yl)-2H-chromen-6-yl]-4-oxo
6-{[6-(5,7-dihydroxy-4-oxo-3,4-dihydro-2H-1-benzopyran-2-yl)-2-(1-hydroxy-4-methylpent-3-en-1-yl)-2-methyl-2H-c
3,4,5-trihydroxy-6-({5-hydroxy-2-[7-hydroxy-2-(1-hydroxy-4-methylpent-3-en-1-yl)-2-methyl-2H-chromen-6-yl]-4-ox
3,4,5-trihydroxy-6-({7-hydroxy-2-[7-hydroxy-2-(1-hydroxy-4-methylpent-3-en-1-yl)-2-methyl-2H-chromen-6-yl]-4-ox
6-({5,7-dihydroxy-2-[7-hydroxy-2-methyl-2-(4-methylpent-3-en-1-yl)-2H-chromen-6-yl]-4-oxo-3,4-dihydro-2H-1-ben
3,4,5-trihydroxy-6-{[2-methyl-2-(4-methylpent-3-en-1-yl)-6-(5,6,7-trihydroxy-4-oxo-3,4-dihydro-2H-1-benzopyran-2-
6-({5,6-dihydroxy-2-[7-hydroxy-2-methyl-2-(4-methylpent-3-en-1-yl)-2H-chromen-6-yl]-4-oxo-3,4-dihydro-2H-1-ben
6-({6,7-dihydroxy-2-[7-hydroxy-2-methyl-2-(4-methylpent-3-en-1-yl)-2H-chromen-6-yl]-4-oxo-3,4-dihydro-2H-1-ben
6-{[6-(5,7-dihydroxy-4-oxo-3,4-dihydro-2H-1-benzopyran-2-yl)-2-(5-hydroxy-4-methylpent-3-en-1-yl)-2-methyl-2H-c
3,4,5-trihydroxy-6-({5-hydroxy-2-[7-hydroxy-2-(5-hydroxy-4-methylpent-3-en-1-yl)-2-methyl-2H-chromen-6-yl]-4-ox
3,4,5-trihydroxy-6-({7-hydroxy-2-[7-hydroxy-2-(5-hydroxy-4-methylpent-3-en-1-yl)-2-methyl-2H-chromen-6-yl]-4-ox
3,4,5-trihydroxy-6-[(5-hydroxy-2-{7-hydroxy-2-[(3E)-5-hydroxy-4-methylpent-3-en-1-yl]-2-methyl-2H-chromen-6-yl}-
6-{[6-(5,7-dihydroxy-4-oxo-3,4-dihydro-2H-1-benzopyran-2-yl)-2-(2-hydroxy-4-methylpent-3-en-1-yl)-2-methyl-2H-c
3,4,5-trihydroxy-6-({5-hydroxy-2-[7-hydroxy-2-(2-hydroxy-4-methylpent-3-en-1-yl)-2-methyl-2H-chromen-6-yl]-4-ox
3,4,5-trihydroxy-6-({7-hydroxy-2-[7-hydroxy-2-(2-hydroxy-4-methylpent-3-en-1-yl)-2-methyl-2H-chromen-6-yl]-4-ox
6-{[6-(5,7-dihydroxy-4-oxo-3,4-dihydro-2H-1-benzopyran-2-yl)-2-[2-(3,3-dimethyloxiran-2-yl)ethyl]-2-methyl-2H-chr
6-[(2-{2-[2-(3,3-dimethyloxiran-2-yl)ethyl]-7-hydroxy-2-methyl-2H-chromen-6-yl}-5-hydroxy-4-oxo-3,4-dihydro-2H-1
6-[(2-{2-[2-(3,3-dimethyloxiran-2-yl)ethyl]-7-hydroxy-2-methyl-2H-chromen-6-yl}-7-hydroxy-4-oxo-3,4-dihydro-2H-1
6-{[10-(5,7-dihydroxy-4-oxo-3,4-dihydro-2H-1-benzopyran-2-yl)-5-methyl-5-(4-methylpent-3-en-1-yl)-3,6-dioxatricyc
3,4,5-trihydroxy-6-({5-hydroxy-2-[9-hydroxy-5-methyl-5-(4-methylpent-3-en-1-yl)-3,6-dioxatricyclo[5.4.0.0Â²,â�und
3,4,5-trihydroxy-6-({7-hydroxy-2-[9-hydroxy-5-methyl-5-(4-methylpent-3-en-1-yl)-3,6-dioxatricyclo[5.4.0.0Â²,â�und
6-{[6-(5,7-dihydroxy-4-oxo-3,4-dihydro-2H-1-benzopyran-2-yl)-7-hydroxy-2-methyl-2-(4-methylpent-3-en-1-yl)-2H-c
6-{[6-(5,7-dihydroxy-4-oxo-3,4-dihydro-2H-1-benzopyran-2-yl)-8-hydroxy-2-methyl-2-(4-methylpent-3-en-1-yl)-2H-c
6-({2-[7,8-dihydroxy-2-methyl-2-(4-methylpent-3-en-1-yl)-2H-chromen-6-yl]-5-hydroxy-4-oxo-3,4-dihydro-2H-1-ben
6-({2-[7,8-dihydroxy-2-methyl-2-(4-methylpent-3-en-1-yl)-2H-chromen-6-yl]-7-hydroxy-4-oxo-3,4-dihydro-2H-1-ben
6-{[6-(5,7-dihydroxy-4-oxo-3,4-dihydro-2H-1-benzopyran-2-yl)-7-hydroxy-2-methyl-2-(4-methylpent-3-en-1-yl)-2H-c
6-{[6-(5,7-dihydroxy-4-oxo-3,4-dihydro-2H-1-benzopyran-2-yl)-5-hydroxy-2-methyl-2-(4-methylpent-3-en-1-yl)-2H-c

6-({2-[5,7-dihydroxy-2-methyl-2-(4-methylpent-3-en-1-yl)-2H-chromen-6-yl]-5-hydroxy-4-oxo-3,4-dihydro-2H-1-ben[illegible]
6-({2-[5,7-dihydroxy-2-methyl-2-(4-methylpent-3-en-1-yl)-2H-chromen-6-yl]-7-hydroxy-4-oxo-3,4-dihydro-2H-1-ben[illegible]
Enalapril
Remifentanil
Raloxifene

m/z	Retention time (min)
5.101.826.561	3.181.826.667
5.101.826.561	3.181.826.667
5.101.826.561	3.181.826.667
5.101.826.561	3.181.826.667
5.101.826.561	3.181.826.667
5.101.826.561	3.181.826.667
5.101.826.561	3.181.826.667
5.101.826.561	3.181.826.667
4.370.683.986	7.829.883.333
4.370.683.986	7.829.883.333
4.370.683.986	7.829.883.333
4.370.683.986	7.829.883.333
4.370.683.986	7.829.883.333
4.370.683.986	7.829.883.333
4.370.683.986	7.829.883.333
4.370.683.986	7.829.883.333
4.370.683.986	7.829.883.333
4.370.683.986	7.829.883.333
643.229.098	4.278.953.333
643.229.098	4.278.953.333
643.229.098	4.278.953.333
3.411.981.874	3.803.535
3.411.981.874	3.803.535
643.229.098	4.278.953.333
643.229.098	4.278.953.333
643.229.098	4.278.953.333
3.411.981.874	3.803.535
3.411.981.874	3.803.535
6.152.230.062	3.899.876.667
6.152.230.062	3.899.876.667
6.152.230.062	3.899.876.667
6.152.230.062	3.899.876.667
3.411.981.874	3.803.535
3.411.981.874	3.803.535
3.411.981.874	3.803.535
3.411.981.874	3.803.535
1.180.890.398	232.965
1.180.890.398	232.965
1.180.890.398	232.965
1.180.890.398	232.965
1.180.890.398	232.965
1.180.890.398	232.965
1.180.890.398	232.965
1.180.890.398	232.965
1.180.890.398	232.965
1.180.890.398	232.965
4.821.732.969	2.838.971.667
5.101.826.561	3.181.826.667

5.101.826.561	3.181.826.667
5.101.826.561	3.181.826.667
4.902.726.351	3.903.501.667
4.902.726.351	3.903.501.667
4.902.726.351	3.903.501.667
4.902.726.351	3.903.501.667
643.229.098	4.278.953.333
643.229.098	4.278.953.333
643.229.098	4.278.953.333
4.961.463.417	275.346
643.229.098	4.278.953.333
643.229.098	4.278.953.333
643.229.098	4.278.953.333
4.961.463.417	275.346
5.101.826.561	3.181.826.667
5.101.826.561	3.181.826.667
5.101.826.561	3.181.826.667
5.101.826.561	3.181.826.667
4.821.732.969	2.838.971.667
3.411.981.874	3.803.535
3.411.981.874	3.803.535
752.160.809	4.371.788.333
6.292.281.511	4.074.456.667
6.152.185.516	389.353
6.152.185.516	389.353
7.791.615.171	4.444.833.333
7.791.615.171	4.444.833.333
7.791.615.171	4.444.833.333
7.791.615.171	4.444.833.333
7.791.615.171	4.444.833.333
7.791.615.171	4.444.833.333
7.791.615.171	4.444.833.333
7.791.615.171	4.444.833.333
7.791.615.171	4.444.833.333
7.791.615.171	4.444.833.333
7.791.615.171	4.444.833.333
7.791.615.171	4.444.833.333
7.791.615.171	4.444.833.333
4.370.683.986	7.829.883.333
6.152.606.563	3.904.408.333
6.152.606.563	3.904.408.333
6.152.606.563	3.904.408.333
4.370.683.986	7.829.883.333
6.152.606.563	3.904.408.333
6.152.606.563	3.904.408.333
6.152.606.563	3.904.408.333
5.382.029.403	3.538.141.667
6.292.269.961	4.066.476.667
6.292.269.961	4.066.476.667
752.160.809	4.371.788.333

752.160.809	4.371.788.333
752.160.809	4.371.788.333
752.160.809	4.371.788.333
752.160.809	4.371.788.333
752.160.809	4.371.788.333
752.160.809	4.371.788.333
752.160.809	4.371.788.333
752.160.809	4.371.788.333
752.160.809	4.371.788.333
752.160.809	4.371.788.333
752.160.809	4.371.788.333
6.292.281.511	4.074.456.667
3.411.981.874	3.803.535
3.411.981.874	3.803.535
1.560.198.923	0.286783333
6.292.281.511	4.074.456.667
7.791.615.171	4.444.833.333
4.961.463.417	275.346
4.961.463.417	275.346
4.961.463.417	275.346
4.961.463.417	275.346
7.791.615.171	4.444.833.333
4.961.463.417	275.346
6.162.240.845	3.898.061.667
3.411.981.874	3.803.535
4.961.463.417	275.346
4.961.463.417	275.346
4.961.463.417	275.346
4.961.463.417	275.346
566.211.328	392.778
1.560.198.923	0.286783333
1.560.198.923	0.286783333
1.560.198.923	0.286783333
1.560.198.923	0.286783333
1.560.198.923	0.286783333
566.211.328	392.778
4.821.732.969	2.838.971.667
1.400.572.152	0.277583333
1.400.572.152	0.277583333
1.400.572.152	0.277583333
1.400.572.152	0.277583333
4.821.732.969	2.838.971.667
1.400.572.152	0.277583333
1.400.572.152	0.277583333
1.400.572.152	0.277583333
1.400.572.152	0.277583333
5.382.029.403	3.538.141.667
5.382.029.403	3.538.141.667
6.432.386.512	4.300.743.333
6.432.386.512	4.300.743.333

6.432.386.512	4.300.743.333
4.961.463.417	275.346
5.382.029.403	3.538.141.667
5.382.029.403	3.538.141.667
6.432.386.512	4.300.743.333
6.432.386.512	4.300.743.333
6.432.386.512	4.300.743.333
4.961.463.417	275.346
6.152.182.546	387.286
6.152.182.546	387.286
566.211.328	392.778
566.211.328	392.778
566.211.328	392.778
643.229.098	4.278.953.333
1.400.572.152	0.277583333
6.152.182.546	387.286
6.152.182.546	387.286
566.211.328	392.778
566.211.328	392.778
566.211.328	392.778
643.229.098	4.278.953.333
1.400.572.152	0.277583333
6.152.182.546	387.286
6.152.182.546	387.286
6.152.182.546	387.286
6.152.182.546	387.286
6.152.182.546	387.286
6.152.182.546	387.286
6.152.182.546	387.286
6.152.182.546	387.286
6.152.182.546	387.286
6.152.182.546	387.286
6.152.182.546	387.286
6.152.182.546	387.286
6.152.182.546	387.286
6.152.182.546	387.286
6.152.182.546	387.286
6.152.182.546	387.286
6.152.182.546	387.286
6.152.182.546	387.286
6.152.182.546	387.286
6.152.182.546	387.286
6.152.182.546	387.286
6.152.182.546	387.286
6.152.182.546	387.286
6.152.182.546	387.286
6.152.182.546	387.286
6.152.182.546	387.286
6.152.182.546	387.286
6.152.182.546	387.286
6.152.182.546	387.286

6.152.182.546	387.286
6.152.182.546	387.286
6.152.182.546	387.286
6.152.182.546	387.286
6.152.182.546	387.286
6.152.182.546	387.286
6.152.182.546	387.286
6.152.182.546	387.286
6.152.182.546	387.286
6.152.182.546	387.286
6.152.182.546	387.286
6.152.182.546	387.286
6.152.182.546	387.286
6.152.182.546	387.286
6.152.182.546	387.286
6.152.182.546	387.286
6.152.182.546	387.286
6.152.182.546	387.286
6.152.182.546	387.286
6.152.182.546	387.286
6.152.182.546	387.286
6.152.182.546	387.286
6.152.182.546	387.286
6.152.182.546	387.286
6.152.182.546	387.286
6.152.182.546	387.286
6.152.182.546	387.286
6.152.182.546	387.286
6.292.281.511	4.074.456.667
6.152.230.062	3.899.876.667
6.152.230.062	3.899.876.667
6.152.185.516	389.353
6.152.185.516	389.353
3.411.981.874	3.803.535
3.411.981.874	3.803.535
3.411.981.874	3.803.535
3.411.981.874	3.803.535
3.411.981.874	3.803.535
3.411.981.874	3.803.535
3.411.981.874	3.803.535
3.411.981.874	3.803.535
3.411.981.874	3.803.535
3.411.981.874	3.803.535
3.411.981.874	3.803.535
3.411.981.874	3.803.535
3.411.981.874	3.803.535
3.411.981.874	3.803.535
3.411.981.874	3.803.535
3.411.981.874	3.803.535
3.411.981.874	3.803.535

3.411.981.874	3.803.535
3.411.981.874	3.803.535
3.411.981.874	3.803.535
3.411.981.874	3.803.535
3.411.981.874	3.803.535
3.411.981.874	3.803.535
3.411.981.874	3.803.535
3.411.981.874	3.803.535
3.411.981.874	3.803.535
3.411.981.874	3.803.535
3.411.981.874	3.803.535
3.411.981.874	3.803.535
3.411.981.874	3.803.535
3.411.981.874	3.803.535
3.411.981.874	3.803.535
3.411.981.874	3.803.535
3.411.981.874	3.803.535
3.411.981.874	3.803.535
3.411.981.874	3.803.535
3.411.981.874	3.803.535
6.152.182.546	387.286
6.152.182.546	387.286
6.152.182.546	387.286
6.152.182.546	387.286
6.152.182.546	387.286
6.152.182.546	387.286
6.152.182.546	387.286
6.152.182.546	387.286
6.152.182.546	387.286
6.152.182.546	387.286
6.152.182.546	387.286
6.152.182.546	387.286
6.152.182.546	387.286
6.152.182.546	387.286
6.152.182.546	387.286
6.152.182.546	387.286
6.152.182.546	387.286
6.152.182.546	387.286
6.152.182.546	387.286
6.152.182.546	387.286
6.152.182.546	387.286
6.152.182.546	387.286
6.152.182.546	387.286
6.152.182.546	387.286
6.152.182.546	387.286
6.152.182.546	387.286
6.152.182.546	387.286
6.152.182.546	387.286
6.152.182.546	387.286
6.152.182.546	387.286

6.152.182.546	387.286
6.152.182.546	387.286
6.152.182.546	387.286
6.152.182.546	387.286
6.152.182.546	387.286
6.152.182.546	387.286
6.152.182.546	387.286
6.152.182.546	387.286
6.152.182.546	387.286
6.152.182.546	387.286
6.152.182.546	387.286
6.152.182.546	387.286
6.152.182.546	387.286
6.152.182.546	387.286
6.152.182.546	387.286
6.152.182.546	387.286
6.152.182.546	387.286
6.152.182.546	387.286
6.152.182.546	387.286
6.152.182.546	387.286
6.152.182.546	387.286
6.152.182.546	387.286
6.152.182.546	387.286
6.152.182.546	387.286
6.152.182.546	387.286
6.152.182.546	387.286
6.292.281.511	4.074.456.667
6.152.230.062	3.899.876.667
6.152.230.062	3.899.876.667
6.152.185.516	389.353
6.152.185.516	389.353
3.411.981.874	3.803.535
3.411.981.874	3.803.535
3.411.981.874	3.803.535
3.411.981.874	3.803.535
3.411.981.874	3.803.535
3.411.981.874	3.803.535
3.411.981.874	3.803.535
3.411.981.874	3.803.535
3.411.981.874	3.803.535
3.411.981.874	3.803.535
3.411.981.874	3.803.535
3.411.981.874	3.803.535
3.411.981.874	3.803.535
3.411.981.874	3.803.535
3.411.981.874	3.803.535
3.411.981.874	3.803.535
3.411.981.874	3.803.535
3.411.981.874	3.803.535
3.411.981.874	3.803.535

3.411.981.874	3.803.535
3.411.981.874	3.803.535
3.411.981.874	3.803.535
3.411.981.874	3.803.535
3.411.981.874	3.803.535
3.411.981.874	3.803.535
3.411.981.874	3.803.535
3.411.981.874	3.803.535
3.411.981.874	3.803.535
3.411.981.874	3.803.535
3.411.981.874	3.803.535
3.411.981.874	3.803.535
3.411.981.874	3.803.535
3.411.981.874	3.803.535
3.411.981.874	3.803.535
3.411.981.874	3.803.535
3.411.981.874	3.803.535
3.411.981.874	3.803.535
6.162.240.845	3.898.061.667
6.162.240.845	3.898.061.667
6.152.230.062	3.899.876.667
6.152.230.062	3.899.876.667
6.152.230.062	3.899.876.667
6.152.230.062	3.899.876.667
6.152.230.062	3.899.876.667
6.152.230.062	3.899.876.667
6.152.230.062	3.899.876.667
6.152.230.062	3.899.876.667
6.152.230.062	3.899.876.667
6.152.230.062	3.899.876.667
6.152.230.062	3.899.876.667
6.152.230.062	3.899.876.667
6.152.230.062	3.899.876.667
6.152.230.062	3.899.876.667
6.152.230.062	3.899.876.667
6.152.230.062	3.899.876.667
6.152.230.062	3.899.876.667
6.152.230.062	3.899.876.667
6.152.230.062	3.899.876.667
6.152.230.062	3.899.876.667
6.152.230.062	3.899.876.667
6.152.230.062	3.899.876.667
6.152.230.062	3.899.876.667
6.152.230.062	3.899.876.667
6.152.230.062	3.899.876.667
6.152.230.062	3.899.876.667
6.152.230.062	3.899.876.667
6.152.230.062	3.899.876.667
6.152.230.062	3.899.876.667
6.152.230.062	3.899.876.667

6.152.230.062	3.899.876.667
6.152.230.062	3.899.876.667
6.152.230.062	3.899.876.667
6.152.230.062	3.899.876.667
6.152.230.062	3.899.876.667
6.152.230.062	3.899.876.667
6.152.230.062	3.899.876.667
6.152.230.062	3.899.876.667
6.152.230.062	3.899.876.667
6.152.230.062	3.899.876.667
6.152.230.062	3.899.876.667
6.152.230.062	3.899.876.667
6.152.230.062	3.899.876.667
6.152.230.062	3.899.876.667
6.152.230.062	3.899.876.667
6.152.230.062	3.899.876.667
6.152.230.062	3.899.876.667
6.152.230.062	3.899.876.667
6.152.230.062	3.899.876.667
6.152.230.062	3.899.876.667
6.152.230.062	3.899.876.667
6.152.230.062	3.899.876.667
6.152.230.062	3.899.876.667
6.152.230.062	3.899.876.667
6.152.230.062	3.899.876.667
6.152.230.062	3.899.876.667
6.152.185.516	389.353
6.152.185.516	389.353
6.152.185.516	389.353
6.152.185.516	389.353
6.152.185.516	389.353
6.152.185.516	389.353
6.152.185.516	389.353
6.152.185.516	389.353
6.152.185.516	389.353
6.152.185.516	389.353
6.152.185.516	389.353
6.152.185.516	389.353
6.152.185.516	389.353
6.152.185.516	389.353
6.152.185.516	389.353
6.152.185.516	389.353
6.152.185.516	389.353
6.152.185.516	389.353
6.152.185.516	389.353
6.152.185.516	389.353
6.152.185.516	389.353
6.152.185.516	389.353
6.152.185.516	389.353
6.152.185.516	389.353

6.152.185.516	389.353
6.152.185.516	389.353
6.152.185.516	389.353
6.152.185.516	389.353
6.152.185.516	389.353
6.152.185.516	389.353
6.152.185.516	389.353
6.152.185.516	389.353
6.152.185.516	389.353
6.152.185.516	389.353
6.152.185.516	389.353
6.152.185.516	389.353
6.152.185.516	389.353
6.152.185.516	389.353
6.152.185.516	389.353
6.152.185.516	389.353
6.152.185.516	389.353
6.152.185.516	389.353
6.152.185.516	389.353
6.152.185.516	389.353
6.152.185.516	389.353
6.152.185.516	389.353
6.152.185.516	389.353
6.152.185.516	389.353
6.152.185.516	389.353
6.152.185.516	389.353
6.152.185.516	389.353
6.152.185.516	389.353
6.152.185.516	389.353
6.152.185.516	389.353
6.152.185.516	389.353
6.152.185.516	389.353
3.411.981.874	3.803.535
3.411.981.874	3.803.535
4.961.463.417	275.346
1.560.198.923	0.286783333
1.560.198.923	0.286783333
1.560.198.923	0.286783333
6.162.240.845	3.898.061.667
6.162.240.845	3.898.061.667
6.152.230.062	3.899.876.667
6.152.230.062	3.899.876.667
6.152.230.062	3.899.876.667
6.152.230.062	3.899.876.667
6.152.230.062	3.899.876.667
6.152.230.062	3.899.876.667
6.152.230.062	3.899.876.667
6.152.230.062	3.899.876.667
6.152.230.062	3.899.876.667
6.152.230.062	3.899.876.667

6.152.230.062	3.899.876.667
6.152.230.062	3.899.876.667
6.152.230.062	3.899.876.667
6.152.230.062	3.899.876.667
6.152.230.062	3.899.876.667
6.152.230.062	3.899.876.667
6.152.230.062	3.899.876.667
6.152.230.062	3.899.876.667
6.152.230.062	3.899.876.667
6.152.230.062	3.899.876.667
6.152.230.062	3.899.876.667
6.152.230.062	3.899.876.667
6.152.230.062	3.899.876.667
6.152.230.062	3.899.876.667
6.152.230.062	3.899.876.667
6.152.230.062	3.899.876.667
6.152.230.062	3.899.876.667
6.152.230.062	3.899.876.667
6.152.230.062	3.899.876.667
6.152.230.062	3.899.876.667
6.152.230.062	3.899.876.667
6.152.230.062	3.899.876.667
6.152.230.062	3.899.876.667
6.152.230.062	3.899.876.667
6.152.230.062	3.899.876.667
6.152.230.062	3.899.876.667
6.152.230.062	3.899.876.667
6.152.230.062	3.899.876.667
6.152.230.062	3.899.876.667
6.152.230.062	3.899.876.667
6.152.230.062	3.899.876.667
6.152.230.062	3.899.876.667
6.152.230.062	3.899.876.667
6.152.230.062	3.899.876.667
6.152.230.062	3.899.876.667
6.152.230.062	3.899.876.667
6.152.230.062	3.899.876.667
6.152.230.062	3.899.876.667
6.152.230.062	3.899.876.667
6.152.230.062	3.899.876.667
6.152.230.062	3.899.876.667
6.152.230.062	3.899.876.667
6.152.230.062	3.899.876.667
6.152.230.062	3.899.876.667
6.152.230.062	3.899.876.667
6.152.230.062	3.899.876.667
6.152.185.516	389.353
6.152.185.516	389.353
6.152.185.516	389.353
6.152.185.516	389.353

6.152.185.516	389.353
6.152.185.516	389.353
6.152.185.516	389.353
6.152.185.516	389.353
6.152.185.516	389.353
6.152.185.516	389.353
6.152.185.516	389.353
6.152.185.516	389.353
6.152.185.516	389.353
6.152.185.516	389.353
6.152.185.516	389.353
6.152.185.516	389.353
6.152.185.516	389.353
6.152.185.516	389.353
6.152.185.516	389.353
6.152.185.516	389.353
6.152.185.516	389.353
6.152.185.516	389.353
6.152.185.516	389.353
6.152.185.516	389.353
6.152.185.516	389.353
6.152.185.516	389.353
6.152.185.516	389.353
6.152.185.516	389.353
6.152.185.516	389.353
6.152.185.516	389.353
6.152.185.516	389.353
6.152.185.516	389.353
6.152.185.516	389.353
6.152.185.516	389.353
6.152.185.516	389.353
6.152.185.516	389.353
6.152.185.516	389.353
6.152.185.516	389.353
6.152.185.516	389.353
6.152.185.516	389.353
6.152.185.516	389.353
6.152.185.516	389.353
6.152.185.516	389.353
6.152.185.516	389.353
6.152.185.516	389.353
6.152.185.516	389.353
6.152.185.516	389.353
6.152.185.516	389.353
6.152.185.516	389.353
6.152.185.516	389.353
6.152.185.516	389.353
6.152.185.516	389.353
6.152.185.516	389.353
6.152.185.516	389.353

6.152.185.516	389.353
6.152.185.516	389.353
3.411.981.874	3.803.535
3.411.981.874	3.803.535
4.961.463.417	275.346
